# Distinct Pathogenesis and Host Responses during Infection of *C. elegans* by *P. aeruginosa* and *S. aureus*

**DOI:** 10.1371/journal.ppat.1000982

**Published:** 2010-07-01

**Authors:** Javier E. Irazoqui, Emily R. Troemel, Rhonda L. Feinbaum, Lyly G. Luhachack, Brent O. Cezairliyan, Frederick M. Ausubel

**Affiliations:** 1 Program of Developmental Immunology, Department of Pediatrics, Massachusetts General Hospital, Department of Pediatrics, Harvard Medical School, Boston, Massachusetts, United States of America; 2 Section of Cell and Developmental Biology, University of California, San Diego, La Jolla, California, United States of America; 3 Department of Molecular Biology, Massachusetts General Hospital, Department of Genetics, Harvard Medical School, Boston, Massachusetts, United States of America; University of Toronto, Canada

## Abstract

The genetically tractable model host *Caenorhabditis elegans* provides a valuable tool to dissect host-microbe interactions *in vivo*. *Pseudomonas aeruginosa* and *Staphylococcus aureus* utilize virulence factors involved in human disease to infect and kill *C. elegans*. Despite much progress, virtually nothing is known regarding the cytopathology of infection and the proximate causes of nematode death. Using light and electron microscopy, we found that *P. aeruginosa* infection entails intestinal distention, accumulation of an unidentified extracellular matrix and *P. aeruginosa*-synthesized outer membrane vesicles in the gut lumen and on the apical surface of intestinal cells, the appearance of abnormal autophagosomes inside intestinal cells, and *P. aeruginosa* intracellular invasion of *C. elegans*. Importantly, heat-killed *P. aeruginosa* fails to elicit a significant host response, suggesting that the *C. elegans* response to *P. aeruginosa* is activated either by heat-labile signals or pathogen-induced damage. In contrast, *S. aureus* infection causes enterocyte effacement, intestinal epithelium destruction, and complete degradation of internal organs. *S. aureus* activates a strong transcriptional response in *C. elegans* intestinal epithelial cells, which aids host survival during infection and shares elements with human innate responses. The *C. elegans* genes induced in response to *S. aureus* are mostly distinct from those induced by *P. aeruginosa*. In contrast to *P. aeruginosa*, heat-killed *S. aureus* activates a similar response as live *S. aureus*, which appears to be independent of the single *C. elegans* Toll-Like Receptor (TLR) protein. These data suggest that the host response to *S. aureus* is possibly mediated by pathogen-associated molecular patterns (PAMPs). Because our data suggest that neither the *P. aeruginosa* nor the *S. aureus*–triggered response requires canonical TLR signaling, they imply the existence of unidentified mechanisms for pathogen detection in *C. elegans*, with potentially conserved roles also in mammals.

## Author Summary

*Pseudomonas aeruginosa* and *Staphylococcus aureus* are bacteria that can form part of the human microbiota, but can also cause severe disease. Despite their great clinical importance, little is known about their interactions with the human host before disease onset, particularly regarding the molecules that host cells use to prevent and combat infection. The invertebrate *Caenorhabditis elegans* is a powerful model host to study host-pathogen interactions and, because of its simplicity and highly-developed experimental methods, can illuminate fundamental mechanisms of bacterial pathogenesis. We used *C. elegans* to understand the cellular events that occur during early stages of *P. aeruginosa* and *S. aureus* infection. We found that *P. aeruginosa* slowly colonizes the intestine, producing virulence-related membrane vesicles, and causing accumulation of electron-dense biofilm-like material on intestinal cells and abnormalities in them. In contrast, *S. aureus* colonizes rapidly, disrupting microvilli and lysing host cells. We found that these different strategies result in different host gene expression responses. Focusing on *S. aureus* infection, the *C. elegans* response is mainly microbicidal and detoxifying, aiding host survival and bearing similarities with the human response. Our study provides new insights into mechanisms that *P. aeruginosa* and *S. aureus* use to cause disease, and into *C. elegans* defenses, with potential implications for human immunity and disease.

## Introduction

The study of host-microbe interactions seeks to understand the symbiotic relationships between hosts and microbiota, and their perversion during infectious disease. Essential steps are the identification of bacterial virulence mechanisms and of host defense pathways. In mammalian hosts, Nod-like receptors (NLRs), Toll-like receptors (TLRs), and NF-κB play important roles in the intestinal epithelium, a critical interface of contact between host and microbiota [1], [2], [3]. However, how these signaling pathways function in the context of the whole organism is poorly understood, and potentially novel pathways may yet be uncovered. Likewise, the critical initial stages of infection, before the onset of overt pathogenesis, are poorly defined.

Genetically tractable invertebrate model systems have aided efforts to identify evolutionarily conserved components of the innate immune system [4]. For example, studies using *Drosophila melanogaster* showed the central importance of the Toll and IMD signaling pathways for the regulation of Relish-family (NF-κB) transcription factors [3], [5]. Likewise, studies using *Caenorhabditis elegans* revealed the involvement of evolutionarily conserved p38 MAPK, insulin, TGF-β, and β-catenin signaling pathways [6], [7], [8]. In addition to being a genetically tractable model system, *C. elegans* is a transparent bacterivore, which allows the direct, real-time observation of infection and gene expression reporters *in vivo*. These qualities make it a useful model host for the study of infection and host defense in the context of the whole organism [9]. *C. elegans* is particularly useful for studying intestinal epithelial innate defenses, because it has only 20 such cells that are not shed (as are mammalian intestinal epithelia) and are non-renewable [9], [10], allowing the study of defense functions *in vivo* without potentially confounding cell proliferation and tissue repair. Furthermore, the unique biology of *C. elegans* allows researchers to focus entirely on epithelial innate defense because it lacks a circulatory system, macrophage-like professional immune phagocytes, and antibody-based adaptive immunity [11].

On the bacterial side, it is important to elucidate the virulence mechanisms that defeat host defenses and establish infection. Pathogenic bacteria are thought to have experienced stepwise additions of virulence factors, as they evolved to survive different host antimicrobial responses and to colonize new niches [12]. Our studies using *C. elegans* as a model host may thus interrogate early steps in the evolution of bacteria as pathogens, and their interactions with prototypical metazoan epithelial cells. Here we focus on two paradigmatic human pathogenic bacteria of great medical importance that represent two broad categories of evolutionarily distant microbes, the Gram-negative *Pseudomonas aeruginosa* and Gram-positive *Staphylococcus aureus. P. aeruginosa* causes systemic acute infections in patients with weakened immune systems [13] and establishes chronic infections in the lungs of cystic fibrosis patients [14]. *P. aeruginosa* can also infect a wide variety of plants, metazoans, and single-celled eukaryotes [15]. *S. aureus* is a Gram-positive bacterium that can cause severe diseases in many animal species [16], [17]. In recent years, patients lacking classical risk factors have suffered increasing rates of infection by virulent antibiotic-resistant strains [18]. Human colonization by *S. aureus* is widespread: 30% of the population carries *S. aureus* in the microflora of epithelia in the nasopharynx, skin, and intestine [19]. *S. aureus* can cause severe skin infections, osteomyelitis, endocarditis, food poisoning, pneumonia, and flesh-eating disease [20], for which it deploys an impressive armamentarium of virulence factors, including cytolysins that cause the destruction of host immune cells and tissues [21], [22]. Despite great progress in their identification, the exact contribution of each virulence strategy to disease *in vivo* is poorly understood. The genetic makeup of the host is suspected to determine susceptibility to infection, but the genetic determinants of susceptibility are unknown [20]. Mice lacking adaptive immunity survive intravenous *S. aureus* infection as well as wild-type animals, suggesting that innate immunity is the main clearing mechanism for *S. aureus* infection in mammals, but the exact mechanism is unclear [23]. New approaches are needed to understand the molecular basis of innate host defenses against *P. aeruginosa* and *S. aureus* infection.

To this end, our laboratory has developed *C. elegans-P. aeruginosa* [24], [25] and *C. elegans-S. aureus* [17], [26] model systems to facilitate the study of the role of innate host defenses in conferring resistance to bacterial infections and to identify host signaling pathways relevant to defense [7], [27], [28], [29]. These infection models recapitulate key aspects of *P. aeruginosa* or *S. aureus* disease in mammals (see below), including the requirement of virulence factors necessary for mammalian infection, and have been used to identify novel *P. aeruginosa* and *S. aureus* virulence factors [17], [30], [31], [32]. Despite great progress in the dissection of *C. elegans* host defense signaling pathways since the initial description of the system in 1999 [9], [24], [33], [34], little information has been available on the cellular basis of bacterial pathogenesis and nematode killing.

In this study, we focused on the interactions between *C. elegans* intestinal cells —as prototypical metazoan epithelial cells, and as the first line of defense against intestinal infection—and *P. aeruginosa* or *S. aureus*. We investigated the cytopathologies that occur during infection, which suggest distinct mechanisms of virulence used by each bacterial species *in vivo*. With *P. aeruginosa*, we found that initial intestinal distention, putative outer membrane vesicle (OMV) production, and extracellular matrix accumulation on the intestinal cell brush border are followed by host autophagic abnormalities, intracellular invasion, and penetration of the epithelial barrier. Similarly, previous studies found that *P. aeruginosa* forms biofilms in the lungs of infected patients, where OMV production is also evident [35], [36]. In contrast, faster accumulation of *S. aureus* in the *C. elegans* intestine resulted in enterocyte effacement and loss of intestinal cell volume, followed by intestinal epithelial cell lysis and bacterial invasion of the rest of the body, with complete degradation of host internal tissues. Likewise, previous studies showed intestinal colonization by *S. aureus*, enterocyte effacement in rabbits and neonates, and toxin-mediated cell lysis both *in vitro* and *in vivo* [37], [38], [39], [40], [41], [42], [43].

We also evaluated the differential impact of these distinct pathogenic processes on host gene transcription. We previously defined the host transcriptional response to *P. aeruginosa* infection [44]. To understand if and how the host responds to different virulence mechanisms by employing distinct transcriptional host responses, here we defined the host response to *S. aureus*. The two responses show minimal overlap; the response to *S. aureus* apparently involves host damage- and TLR-independent recognition of microbial molecules, potentially pathogen-associated molecular patterns (PAMPs), whereas *C. elegans* may sense *P. aeruginosa*-derived heat-labile signals or pathogen-elicited damage. Using functional genomics, we identified host factors critical for host defense against *S. aureus*, some of which are analogous to human innate defense factors. These observations advance our knowledge of bacterial pathogenesis in *C. elegans*, and show that the *C. elegans* infection model illuminates evolutionarily conserved mechanisms of bacterial pathogenesis and epithelial host defense.

## Results

### *P. aeruginosa* infection: intestinal distention, extracellular material accumulation, intracellular invasion, outer membrane vesicles, and abnormal autophagy

**Figure 1:**
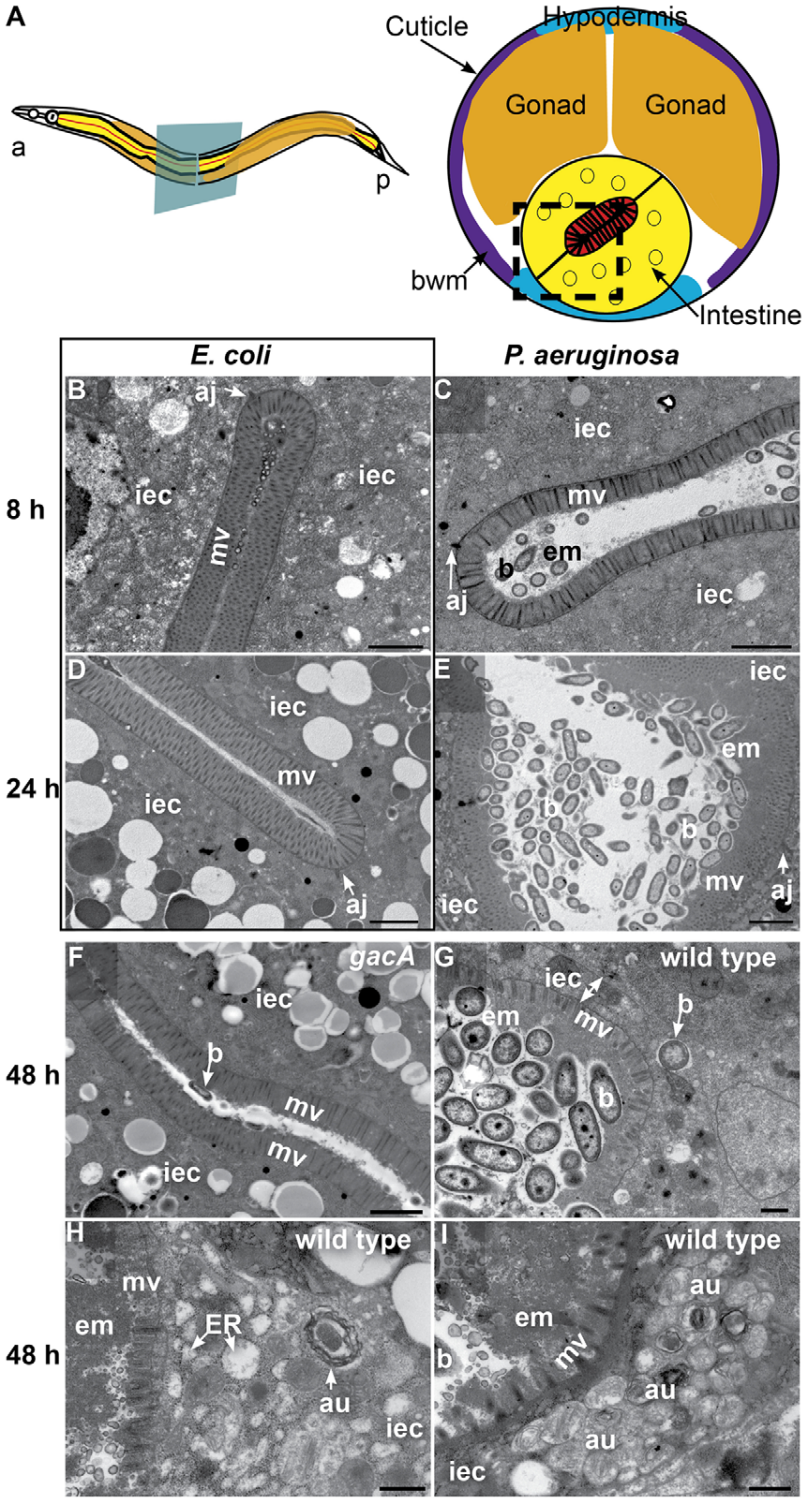
*P. aeruginosa* causes intestinal distention, extracellular material accumulation, intracellular invasion, and abnormal autophagy. **A**. Schematic representation of *C. elegans* body plan and plane of section (Left) and midbody transversal section indicating major organs (Right). Square highlights area of focus in TEM micrographs. **B–I**. TEM micrographs of transversal midbody sections of animals feeding on non-pathogenic *E. coli* OP50(**B, D**), virulent *P. aeruginosa* PA14(**C, E, G, H, I**), or attenuated *gacA P. aeruginosa* PA14 (**F**) for 8 h (**B, C**), 24 h (**D, E**), or 48 h (**F–I**). Scale bar in **B–F**, 2 µm; in **G–I**, 0.5 µm. iec, intestinal epithelial cell; mv, microvilli; b, bacterial cell; bwm, body wall muscle; aj, apical junctions; em, extracellular material; au, arrested autophagosomes, ER, expanded endoplasmic reticulum.

To determine the cytopathology of *P. aeruginosa* colonization of the *C. elegans* intestine, we used transmission electron microscopy (TEM) of the intestinal epithelium (Figure 1A) to evaluate signs of pathogenesis at early times (8 h) or at later times (24 h and 48 h) of infection. At 8 h of infection, we found gross intestinal distention but little bacterial accumulation. Instead, we observed unidentified electron-dense extracellular material accumulating on the apical surface of the brush border (Figure 1C). In addition to coating the brush border, the electron-dense material surrounded bacterial cells that appeared intact and formed clumps in the intestinal lumen. Also surrounding the bacteria and in contact with the extracellular material, we found abundant accumulation of putative outer membrane vesicles (OMVs) (Figure S1A, B). *P. aeruginosa* OMVs have been shown to act as a virulence factor and toxin delivery mechanism [45]. We did not observe intestinal distention, OMVs, or matrix accumulation in *E. coli-*fed control animals (Figure 1B).

At 24 h of infection, the intestine became further distended, with noticeably more bacterial cells accumulating in the lumen in the form of clumps of cells surrounded by extracellular matrix (Figure 1E). There was a thick layer of matrix material coating the microvilli, which were present and of approximately normal length (*i.e.*, ∼1 µm). In contrast, *E. coli-*fed animals lacked these signs of pathogenesis, exhibiting non-distended intestinal lumina and intestinal epithelial cells filled with lipid droplets and other gut granules characteristic of healthy animals (Figure 1D).

At 48 h of infection, pathogenesis advanced further, resulting in higher levels of bacterial accumulation in the grossly distended intestinal lumen (Figure 1G). The bacterial cells were mostly not in direct contact with the microvillar surface, but separated from it by a thick layer of extracellular material. At this time, there was widespread shortening of the microvilli and intracellular invasion by the bacteria (Figure 1G). Intracellular invasion was observed in 21% of cross sections (N = 14), only after 48 h infection. In some cases, we found bacterial cells at distal sites beyond the intestine, suggesting that *P. aeruginosa* can penetrate the intestinal cells and invade other tissues (Figure S1C). In addition to these phenotypes, we observed an increased number of autophagosomes, readily identifiable by their multi-membranous structure (Figure 1H and S2). Indeed, most autophagosomes appeared to be either early autophagosomes (Figure 1H) or aberrant multivesicular autophagosomes (Figure 1I). In contrast, mutant *gacA P. aeruginosa*, lacking the master virulence regulator GacA and therefore attenuated for *C. elegans* killing [25], caused much lower levels of autophagosome accumulation (Figure S2), pathogenesis (Figure 1F), and OMVs (Figure S1D) by 48 h. Intracellular PA14 *gacA* was not observed, even after 72 h (N = 15). We observed less dense matrix accumulation in *gacA* mutant-infected animals than with wild type *P. aeruginosa*, and did not observe microvillar shortening, intracellular invasion, or severe luminal distention. At all times during the infection by both wild-type and *gacA P. aeruginosa*, we only observed what appeared to be live *P. aeruginosa* cells, in contrast to *S. aureus* as described below.

### *S. aureus* infection: anal deformation, intestinal distention, enterocyte effacement, and cell lysis

Interestingly, the cytopathology of *S. aureus* infection in the *C. elegans* intestine is markedly different from a *P. aeruginosa* infection. First, using GFP-labeled *S. aureus*, we observed rapid accumulation of bacteria in the intestine 4 h after infection initiation, whereas *P. aeruginosa* did not start accumulating until 8 after initial exposure (Figure S4A, B). At 4 h, *S. aureus* accumulated in the anterior and posterior ends of the intestine, and the rectum (Figure S3A, C), with less accumulation in the mid section of the intestinal lumen, where the bacteria appeared to be adhering to the apical surface of intestinal cells (Figure S3B). *S. aureus* accumulated further over the course of the following 4 h (Figure S4B). The infected animals moved slowly, were smaller (Figure S4C, D), and appeared to produce fewer eggs, than healthy animals.

In addition to the intestinal distention and accumulation phenotypes, we observed a marked deformation of the anal region with *S. aureus* (Figure S4C) but not with *P. aeruginosa* (not shown) or non-pathogenic *E. coli* (Figure S4D). This deformed anal region (Dar) phenotype [46] appeared 4–8 h after initiation of infection and required live *S. aureus* (Figure S4E). Interestingly, this Dar phenotype was dependent on *bar-1*/β-catenin and *mpk-1*/extracellular signal regulated kinase (ERK) (Figures S4F, G, J, K, and L), which is also required for the Dar response to *Microbacterium nematophilum* [47]. We previously showed that *bar-1*/β-catenin and its downstream target gene *egl-5*/HOX exhibit a defective intestinal response to *S. aureus* [7]. Unexpectedly, mutants defective in *egl-5*/HOX exhibited a wild-type Dar response (Figure S4H, L), despite having an altered intestinal host response to *S. aureus* [7] and a defective anal swelling response to *M. nematophilum* infection [48]. Similarly, *pmk-1*/p38 MAPK mutants exhibited a slightly less noticeable, but equally frequent, Dar phenotype following *S. aureus* infection (Figure S4I, L), consistent with our previous observation that *pmk-1* mutants are only subtly more susceptible to *S. aureus-*mediated killing [7]. These data suggest that the Dar phenotype may be a defensive host swelling response to pathogen-mediated host damage, since it requires an active host response to live bacteria.

**Figure 2:**
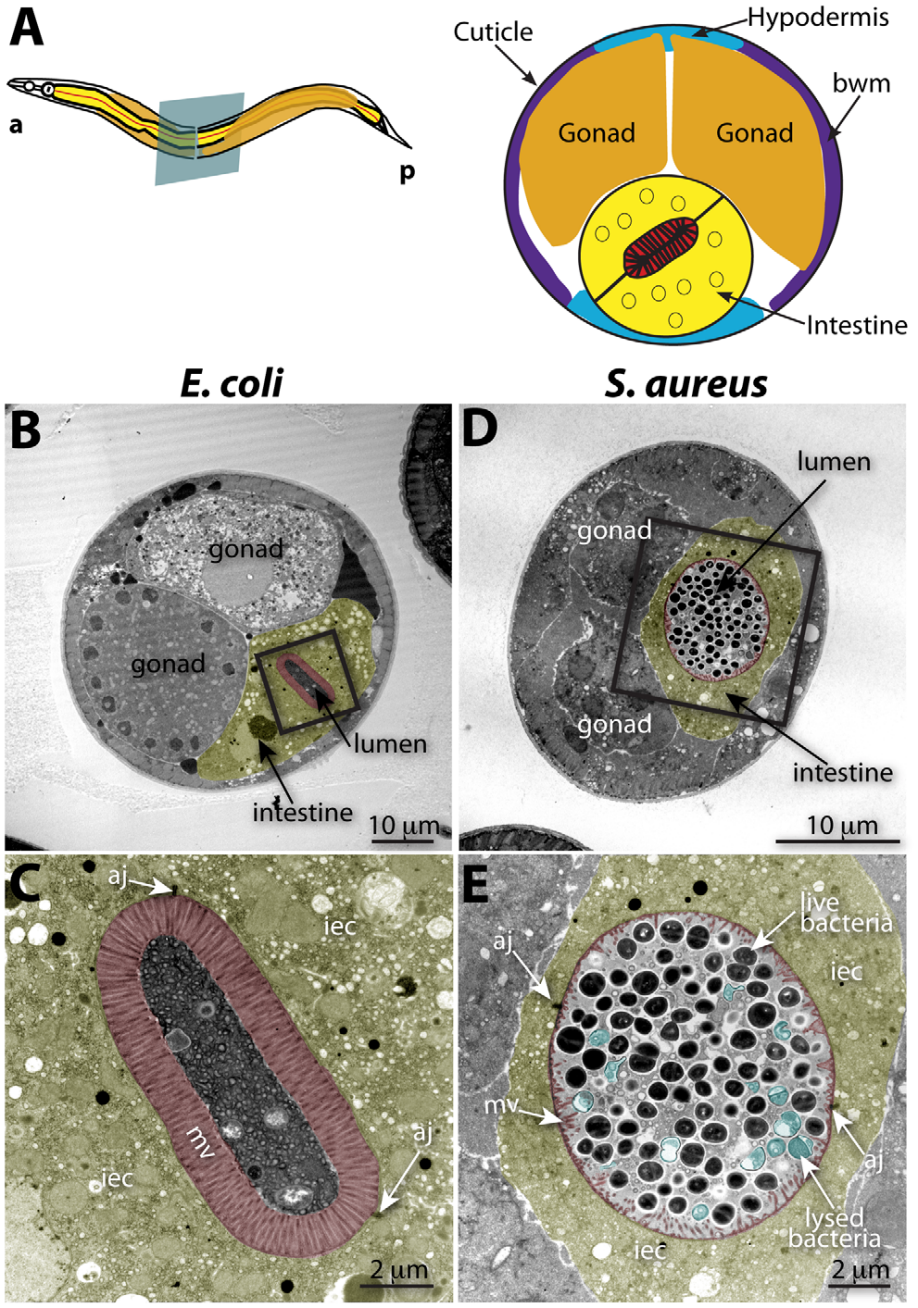
Intestinal distention, enterocyte effacement, and bacterial lysis at 12 h of *S. aureus* infection in *C. elegans*. **A**. Schematic representation of *C. elegans* body plan and plane of section (Left) and midbody transversal section indicating major organs (Right). **B**. Midbody transversal section of a control animal fed non-pathogenic *E. coli*. Square indicates area magnified in **C**. **C**. Higher magnification showing healthy microvilli (mv), intestinal epithelial cells (iec), apical junctions (aj), bacteria and bacterial debris in the intestinal lumen. **D**. Midbody transversal section of an animal infected with *S. aureus* for 12 h. Square indicates area magnified in **E**. **E**. Higher magnification showing short or absent microvilli, shrunken intestinal epithelial cells, apical junctions, and live and lysed bacteria in the distented intestinal lumen. Microvilli are false-colored red from the terminal web to the apical membrane, and intestinal epithelial cells are highlighted in yellow. Lysed bacteria in **E**. are false-colored blue.

**Figure 3:**
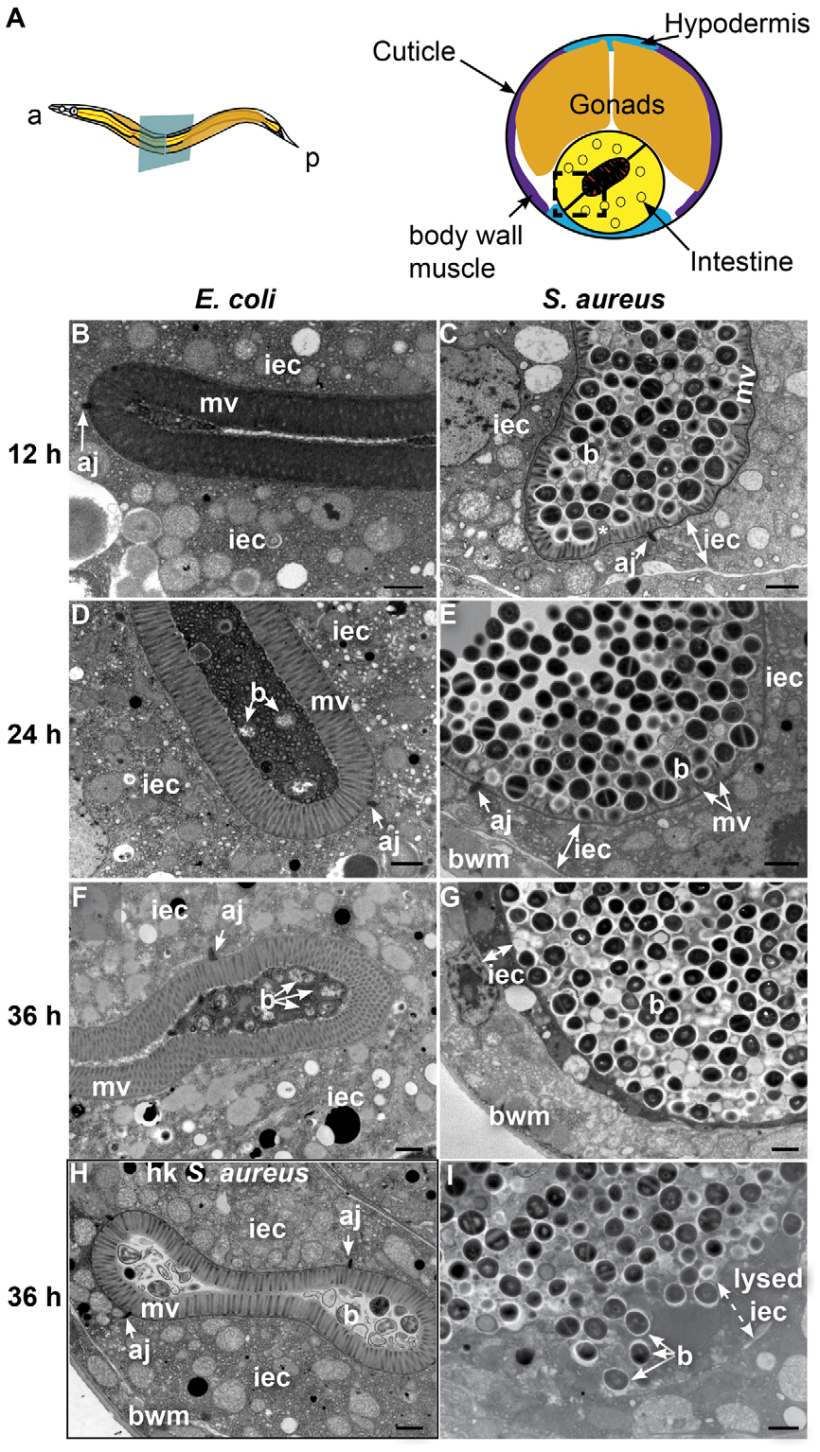
*S. aureus* causes intestinal cell effacement and lysis in *C. elegans*. **A**. Schematic representation of *C. elegans* body plan and plane of section (Left) and midbody transversal section indicating major organs (Right). Square highlights area of focus in TEM micrographs. **B–I**. TEM micrographs of transversal midbody sections of animals feeding on heat-killed non-pathogenic *E. coli* OP50 (**B, D, F**), virulent *S. aureus* NCTC8325 (**C, E, G, I**), or heat-killed *S. aureus* (**H**) for 12 h (**B, C**), 24 h (**D, E**), or 36 h (**F–I**). Scale bar, 1 µm. iec, intestinal epithelial cell; mv, microvilli; b, bacterial cell; bwm, body wall muscle; aj, apical junctions, asterisk in **C** indicates membrane blebbing.

To investigate the cytopathology of *S. aureus* infection, we performed TEM of *S. aureus*-infected animals, focusing on the intestinal epithelium (Figure 2A). After 12 h of infection, we found a striking decrease in the length of the microvilli compared to animals feeding on non-pathogenic *E. coli* (Figures 2B–E, 3B–C). We also observed significant plasma membrane “blebbing” from the apical surface of intestinal cells (Figures 2D–E, 3C, S6A–B). The intestinal lumina of *S. aureus*-infected animals were markedly distended, consistent with our previous observations using light microscopy [17]. Distention was apparently a consequence of severe volume loss of the intestinal epithelial cells, with concomitant accumulation of bacterial cells in the enlarged luminal space (Figure 2B, D). In marked contrast to *P. aeruginosa*, an average 34% of *S. aureus* cells in the lumen (9–63%, N = 8 cross sections) were lysed at 12 h of infection (Figure 2E); these appeared similar to published TEM micrographs showing *S. aureus* cells killed with antimicrobial peptides *in vitro* [49], suggesting that the *C. elegans* intestine may produce bactericidal factors active against *S. aureus*.

In contrast to animals feeding on *E. coli*, at 24 h of infection the microvilli were almost completely destroyed (Figure 3D, E) and at 36 h were completely absent, in what is termed “enterocyte effacement” (Figure 3F, G, I). Further, at 36 h we observed a reduction of intestinal cell volume (see thin sliver in Figure 3G) and intestinal cell lysis (Figure 3I). Also at 36 h, we observed a few dead animals, the organs of which were completely degraded except for the collagenous cuticle and an unidentified circular internal structure (Figure S5). Heat-killed *S. aureus* did not cause intestinal distention, microvillar effacement, intestinal cell lysis, or death (Figure 3H). These data show that *S. aureus* causes membrane and cytoskeletal rearrangements, as well as enterocyte effacement and destruction, possibly by secreted membrane-active bacterial toxins such as cytolysins or other pore-forming toxins (PFTs). Hemolysins α, β, and γ are known *S. aureus* cytolysins. However, they appeared to not be required for pathogenesis and killing, as a *S. aureus* strain lacking all three hemolysins exhibited similar kinetics of *C. elegans* killing as the isogenic wild type (Figure S7). Similarly, the α-hemolysin Δ*hla* mutant was as capable of causing enterocyte effacement, intestinal distention, membrane blebbing, and intestinal cell lysis as wild type (Figure S6C–F). These results indicate that virulence factors other than the hemolysins are responsible for the observed intestinal cell lysis.

### *S. aureus* infection triggers an antimicrobial and detoxifying transcriptional host response

Because host defense from, and digestion of, ingested bacteria are necessarily linked in bacterivorous animals such as *C. elegans*, the distinction between innate immune responses and digestive responses is blurred. Previous studies have investigated the long-term effects of ingestion of pathogenic bacteria, defining a common necrotic host response that is triggered by several pathogens after 24 h of infection [50]. To investigate gene expression changes more likely to be elicited directly by *S. aureus* detection, we evaluated gene expression at an earlier infection time, namely 8 h. We previously reported that *P. aeruginosa* induces a potent transcriptional host response early during infection of *C. elegans*, which significantly contributed to our understanding of *C. elegans* defense from *P. aeruginosa* infection [44]. To determine whether *C. elegans* mounts a similar host response to *S. aureus* infection, we performed whole-genome transcriptional profiling of animals infected with *S. aureus* for 8 h, relative to animals feeding on non-pathogenic *E. coli*. We found 186 transcripts that increased at least two-fold in abundance and 198 that decreased at least two-fold after infection (Table S1). Focusing on 46 genes up-regulated 4-fold or higher as a smaller sample, we found that the majority had potential xenobiotic detoxification or antimicrobial activities, consistent with their involvement in a protective host response (Table 1). In this group, a number of genes of unknown function appeared to encode short secreted polypeptides that may possess antimicrobial activities (Table 1).

**Table 1:** *C. elegans* genes induced 4-fold or higher after 8 h infection with *S. aureus*.

Cosmid Name	Public name	Sequence Description	Fold Change	Presumptive Function*	Expression
***K08C7.5***	***fmo-2***	Flavin-containing monooxygenase FMO	100.0	Detoxification	Intestine, pharynx^1^,^3^
***Y65B4BR.1***	***Y65B4BR.1***	Phospholipase	14.7	Antimicrobial	
***C45G7.3***	***ilys-3***	Invertebrate lysozyme	14.3	Antimicrobial	Intestine, pharynx, vulva^3^
***Y46C8AL.4***	***clec-71***	C-type lectin	9.8	Antimicrobial	
***ZC443.5***	*ugt-18*	UDP-glucoronosyl/UDP-glucosyl transferase	9.4	Detoxification	
***M60.2***	*M60.2*	Placental protein 11	9.2		Intestine^2^
***C48B4.1***	*C48B4.1*	Acyl-CoA oxidase I	8.9	Metabolism	Intestine^2^
***K11D12.4***	*cpt-4*	Acetyltransferase	8.7	Metabolism	Intestine^2^
***K12G11.3***	*sodh-1*	Zinc-containing alcohol dehydrogenase superfamily	8.5	Detoxification	Intestine, pharynx^2^
***F54F3.3***	*F54F3.3*	Lipase	8.4	Antimicrobial	Intestine^2^
***T01C3.4***	*T01C3.4*	Lipase	8.4	Antimicrobial	
***ZK666.6***	***clec-60***	C-type lectin, von Willebrand factor, type A	8.3	Antimicrobial	Intestine^3^
***B0222.4***	*tag-38*	Glutamate decarboxylase	8.3	Metabolism	
***C23G10.11***	*C23G10.11*	Short, basic	7.4	Antimicrobial	
***C45G7.2***	*ilys-2*	Invertebrate lysozyme	7.3	Antimicrobial	
***F46C5.1***	*F46C5.1*	Short, basic	7.0	Antimicrobial	Intestine^2^
***F28G4.1***	*cyp-37B1*	Cytochrome P450	6.9	Detoxification	
***T07G12.5***	*T07G12.5*	Xanthine/uracil/vitamin C permease family	6.9	Detoxification	Intestine, vulva^2^
***K01A2.2***	*far-7*	Nematode fatty acid retinoid binding	6.6	Signaling	
***F38E11.1***	*hsp-12.3*	Heat shock protein Hsp20	6.6	Stress	
***ZK507.4***	*ZK507.4*		6.4		
***T22F3.11***	*T22F3.11*	Permease, major facilitator superfamily transporter	6.3	Detoxification	Intestine^2^
***B0213.15***	*cyp-34A9*	Cytochrome P450	6.1	Detoxification	Intestine^2^
***F09C8.1***	*F09C8.1*	Phospholipase precursor	6.1	Antimicrobial	Intestine^2^
***T09H2.1***	*cyp-34A4*	Cytochrome P450	6.0	Detoxification	
***B0218.8***	***clec-52***	C-type lectin	5.9	Antimicrobial	Intestine, rectal gland^3^,^4^
***F01G10.3***	*ech-9*	Enoyl-CoA hydratase	5.8	Metabolism	
***D1086.5***	*D1086.5*		5.5		hypodermis?^2^
***C34C6.7***	*C34C6.7*		5.4		
***F36D3.9***	***cpr-2***	Cysteine protease	5.4	Antimicrobial	
***K02G10.7***	*aqp-8*	Major intrinsic protein	5.4	Detoxification	Intestine^2^
***Y51H4A.5***	*Y51H4A.5*	Lipase, class 3	5.3	Metabolism	
***F58B3.2***	***lys-5***	Lysozyme	5.2	Antimicrobial	
***C35C5.8***	*C35C5.8*		5.2		
***F53A9.8***	***F53A9.8***	Short His-rich	5.1	Antimicrobial	Intestine, rectal gland^2^,^3^
***Y46C8AL.5***	*clec-72*	C-type lectin	4.9	Antimicrobial	Intestine^2^
***Y5H2B.5***	*cyp-32B1*	Cytochrome P450	4.9	Detoxification	Intestine^2^
***C29F7.2***	*C29F7.2*	DUF1679; Predicted small molecule kinase	4.6	Detoxification	Intestine^2^
***F43C11.7***	*F43C11.7*	Zn-finger, RING	4.5		Intestine^2^
***T07H3.3***	*math-38*	MATH domain	4.5	Signaling	
***M05D6.7***	*gbh-2*	Gamma-butyrobetaine, 2-oxoglutarate dioxygenase	4.4	Metabolism	Hypodermal^2^
***F41C3.1***	*F41C3.1*	Protein of unknown function DUF1265	4.3		
***Y46C8AL.3***	*clec-70*	C-type lectin	4.3	Antimicrobial	Intestine, rectal gland, vulval, uterine, anal depressor muscles^3^
***F46B6.8***	*F46B6.8*	Lipase	4.2	Antimicrobial	
***F58B3.1***	*lys-4*	Lysozyme	4.1	Antimicrobial	
***C50F7.5***	*C50F7.5*	Pro-rich	4.1	Antimicrobial	Intestine, rectal epithelial cells?^2^

References: ^1^ [99],

^2^NextDB (http://nematode.lab.nig.ac.jp/db2/index.php);

^3^This study,

^4^ [100].

*In the absence of further functional characterization, these predictions should be considered speculative.

Bold public names indicate genes selected for qRT-PCR as biomarkers of the response. Functional predictions were based on known homologies and the presence of predicted signal peptides, and should be considered speculative until further functional characterization.

To identify potential physiological roles of this host response, we used two complementary methods to study the over-representation of gene ontology (GO) classes (see Materials and Methods). These analyses revealed up-regulation of detoxifying and antimicrobial responses and down-regulation of growth-related metabolic pathways and extracellular structural components (Table S7). Significance of representation analysis revealed that the most significantly induced gene class contains sugar-binding proteins including C-type lectins (CTLs, N = 15, *p* = 1.2E-5), which could act as signaling receptors, opsonizing agents, or direct antimicrobial effectors [51], [52], [53], [54]. The most significantly repressed GO classes contain genes encoding structural constituents of the cuticle (*e.g.* collagens; N = 47, *p* = 4.4E-25), phosphate and inorganic anion transport (N = 47, *p* = 4.5E-25 and *p* = 5.8E-21), basement membrane components (N = 4, *p* = 1E-6), and lipid transporters (N = 5, *p* = 6E-6). A second approach (see Text S1) expanded these observations to include additional metabolic enzymes and transporters (Table S2). These analyses highlight the potential role of CTLs as a major immune effector strategy used by *C. elegans* during infection by *S. aureus*, and the significant metabolic component of the host response.

### Limited overlap with other stress responses

Because we observed cell membrane rearrangements suggestive of the activity of membrane-active toxins (Figures 2E, 3C, E, G, I, S6), we hypothesized that part of the host response to *S. aureus* may also be triggered by PFTs. Indeed, we found that 22 of 422 probe sets up-regulated during exposure of worms to the *Bacillus thuringiensis* PFT Cry5B [55] were also induced during infection with *S. aureus* (Table S3), significantly higher than the 4 probe sets expected by chance alone. These data suggest that the overlapping set of genes shared by the responses to *S. aureus* and Cry5B may constitute a host response triggered by intestinal cell membrane disruption.

Because we observed evidence of intestinal destruction and nutritional deprivation in animals infected with *S. aureus*, we hypothesized that infected animals might be starving. Previous work identified 18 genes whose expression changed during starvation [56]. In contrast, only one out of 9 previously identified fasting-induced genes (*acs-2*) and 4 out of 9 fasting-repressed genes (*lbp-8, acdh-1, fat-7*, and *F08A8.2*) were affected by *S. aureus* infection. Furthermore, the fasting-induced gene *hacd-1* [56] was *repressed* during infection. These data suggest that the early transcriptional host response to *S. aureus* infection is minimally impacted by the starvation response.

### *S. aureus* infection triggers at least two waves of transcriptional response in the intestinal epithelium

**Figure 4:**
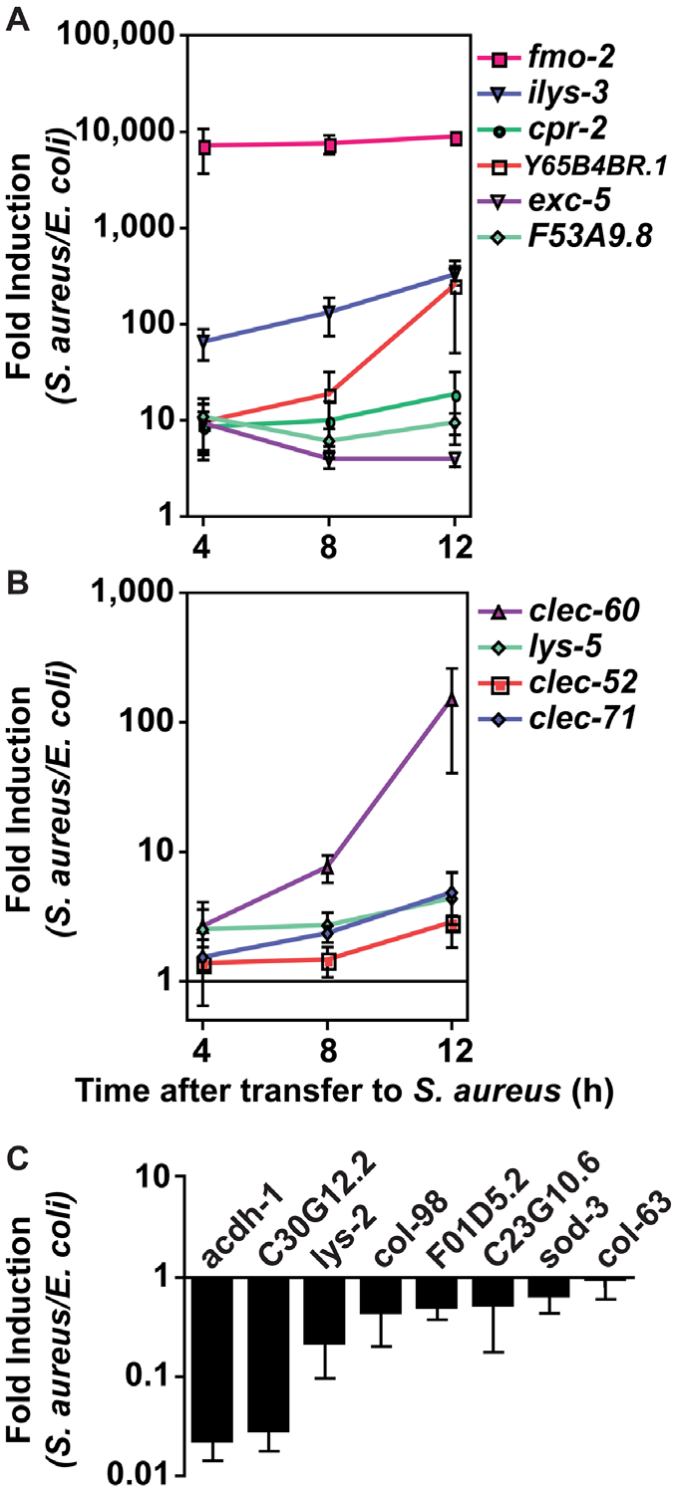
The *C. elegans* host response to *S. aureus* infection is comprised of two kinetic groups. **A, B**. qRT-PCR analysis of genes predicted to be up-regulated by microarray analysis. Transcript levels were measured in synchronized young adult wild-type animals feeding on heat-killed *E. coli* OP50 or infected with *S. aureus* NCTC8325 for 4, 8, and 12 h. Data are the means of three biological replicates, each replicate measured in duplicate and normalized to a control gene, expressed as the ratio of the corresponding *S. aureus-*induced levels and the basal *E. coli* levels. **A**. Immediate-early-induced genes were highly induced by 4 h of infection. **B**. Later induction of early response genes. **C**. qRT-PCR analysis of genes predicted to be repressed by microarray analysis. Transcript levels were measured as in **A**, after 8 h infection with *S. aureus*. Data are the means of three biological replicates, each replicate measured in duplicate and normalized to a control gene, expressed as the ratio of the corresponding *S. aureus-*induced levels and the basal *E. coli* levels. Error bars are SEM.

To validate the microarray experiments, we measured transcript levels for ten selected “biomarker” genes by qRT-PCR over a time course of infection. These ten genes, used as models of the larger host response, were chosen to represent different up-regulation levels and functional annotations (Table 2). All ten genes tested were induced in response to infection (Figure 4A, B). A subset of these biomarkers, *fmo-2* (FMO), *ilys-3* (lysozyme), *cpr-2* (protease), *Y65B4BR.1* (lipase), *exc-5* (GEF), and *F53A9.8* (putative antimicrobial peptide), were already induced 10-fold or higher by the first time-point at 4 h (Figure 4A). A second subset, *lys-5* (lysozyme), *clec-52, clec-60*, and *clec-71* (CTLs), were only modestly induced by 4 h and exhibited a further increase by 12 h (Figure 4B). Thus, time-resolved gene expression analysis revealed the existence of at least two kinetic groups, defined by their expression levels at 4 h. We also measured transcript levels for 8 genes predicted to be repressed upon infection, confirming reduced expression for 7 of them (Figure 4C). Together, these results confirm the predictive value of the genome-wide profiling.

**Table 2:** Selection of 10 biomarker genes to measure the host response to *S. aureus*.

Cosmid Name	Public name	Pathogen-specificity (plus *S. aureus*)*	Fold Change	Prediction
***K08C7.5***	***fmo-2***	*E. faecalis* (UP)	100.0	detoxification enzyme
***Y65B4BR.1***	***Y65B4BR.1***	*S. marcescens* (UP)	14.7	antibacterial lipase
***C45G7.3***	***ilys-3***	*P. luminescens* (UP) *P. aeruginosa* (DOWN)	14.3	lysozyme
***Y46C8AL.4***	***clec-71***	*P. aeruginosa* (UP)	9.8	C-type lectin
***ZK666.6***	***clec-60***	*M. nematophilum* (UP)	8.3	C-type lectin
***B0218.8***	***clec-52***	*P. aeruginosa* (DOWN)	5.9	C-type lectin
***F36D3.9***	***cpr-2***	*E. faecalis* (UP)	5.4	antibacterial protease
***F58B3.2***	***lys-5***	*E. faecalis* (UP) *P. aeruginosa* (DOWN)	5.2	lysozyme
***F53A9.8***	***F53A9.8***	*E. faecalis, P. aeruginosa, M. nematophilum, P. luminescens, Cry5B* (UP)	5.1	antibacterial peptide
***C33D9.1***	***exc-5***	*—*	N/S	CDC-42 GEF

*: based on microarray results; UP: up-regulated; DOWN: down-regulated; N/S: not significant by microarray.

Genes were selected to represent a variety of induction levels, pathogen-specificities, and molecular functions.

**Figure 5:**
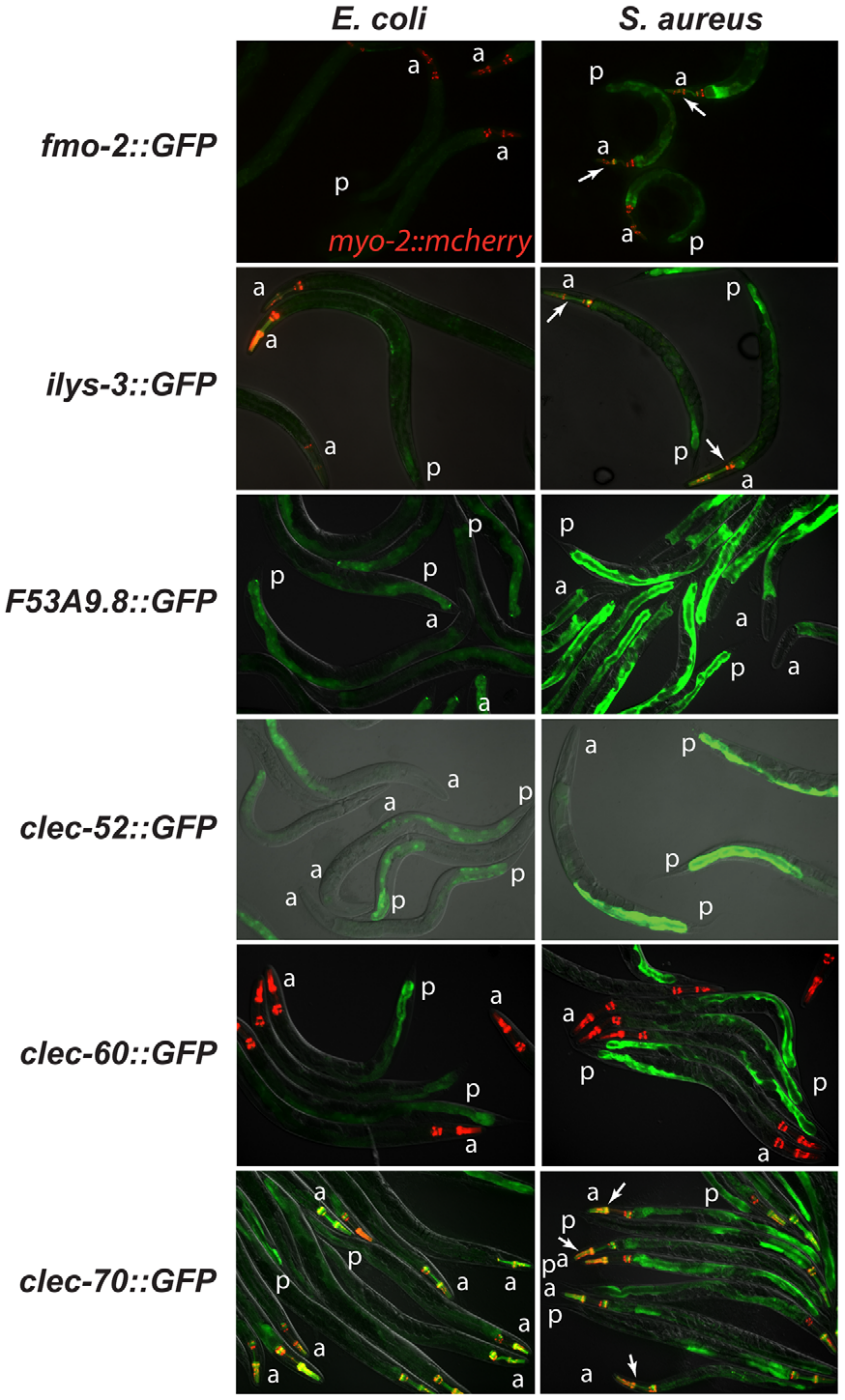
The *C. elegans* host response to *S. aureus* is induced in the intestinal epithelial cells. Transcriptional reporters using upstream sequences to *fmo-2, ilys-3*, *F53A9.8, clec-52, clec-60*, and *clec-70* fused to GFP were induced in the intestinal epithelial cells after 24 h of *S. aureus* NCTC8325 infection (left panels) compared to parallel *E. coli* OP50*-*fed controls (right panels). Despite strong induction of *fmo-2* and *ilys-3* as measured by qRT-PCR, the corresponding GFP reporters exhibited low levels of expression; this could be due to their low basal expression on *E. coli* (not shown). Red, *myo-2::NLS::cherry* coinjection marker expressed in the pharynx in the head. A fold induction of 1 indicates no induction. a, anterior end; p, posterior end. Arrows indicate pharyngeal expression. *ilys-3* was also expressed in the pharynx, in an unidentified cell superimposed on the pharynx, and in unidentified cells, possibly epithelial cells, in the vulva (Fig. S6A,B). *clec-70* was also expressed in unknown cells in the pharynx (Fig. S6C) and in the uterine muscle (Fig. S6D), but only in one transgenic line of three. *F53A9.8* was also expressed in a group of cells surrounding the rectum, possibly the rectal gland cells that secrete molecules into the rectal lumen (Fig. S6G, H).

To elucidate the spatial pattern of the host response, we used transgenic animals carrying transcriptional reporters in which the promoters for 5 of the 10 biomarker genes (*clec-52, clec-60, F53A9.8, fmo-2*, and *ilys-3*), as well as *clec-70*, an additional CTL gene up-regulated by *S. aureus* and important for host defense (see below), were fused to GFP. We infected these transgenic animals with *S. aureus* and compared the intensity and pattern of GFP expression with control animals feeding on *E. coli*. All the genes tested were expressed at low basal levels in the intestines of the latter (Figure 5, left panels). After infection with *S. aureus*, all of the GFP reporters were induced in the intestinal epithelial cells (Figure 5, right panels). *ilys-3, F53A9.8, clec-52, clec-60*, and *clec-70* were all expressed more strongly in the posterior end of the intestine than the anterior. *ilys-3, fmo-2*, and *clec-70* were also induced in the pharynx. Although promoter-GFP fusions like these lack potentially important endogenous regulatory 3′ UTR and intronic sequences, these data are consistent with endogenous RNA localization data from ongoing genome-wide *in situ* hybridization studies of animals feeding on non-pathogenic *E. coli* (NextDB, http://nematode.lab.nig.ac.jp/db2/index.php, and Table 1). Thus, the *C. elegans* transcriptional host response to *S. aureus* is primarily localized in the intestinal epithelial cells and, in some cases, additional sites (Figure S8A–H).

### Distinct modes of detection of *S. aureus* and *P. aeruginosa* infection

**Figure 6:**
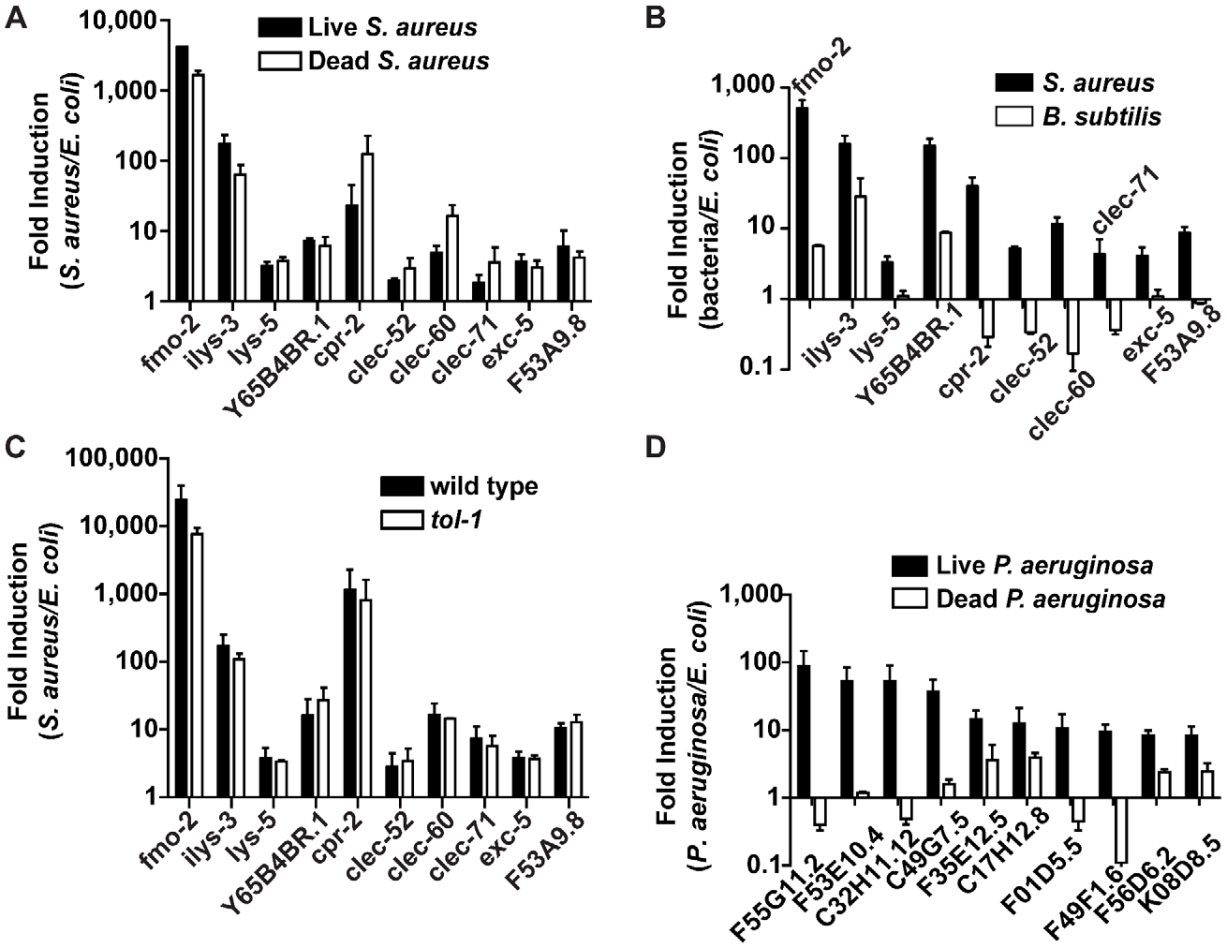
Potential PAMP-mediated, TLR-independent sensing of *S. aureus*. **A**. qRT-PCR analysis of induced genes in animals feeding on heat-killed *S. aureus*. Transcript levels were measured in synchronized young adult wild-type animals infected with live *S. aureus* NCTC8325 or feeding on dead *S. aureus* or *E. coli* OP50 for 8 h. **B**. qRT-PCR analysis of induced genes in animals feeding on *B. subtilis*. Transcript levels were measured in synchronized young adult wild-type animals feeding on *B. subtilis* PY79 or heat-killed *E. coli* for 8 h. **C**. qRT-PCR analysis of induced genes in *tol-1* mutant animals. Transcript levels were measured in synchronized young adult animals feeding on heat-killed *E. coli* or infected with *S. aureus* for 8 h. **D**. qRT-PCR analysis of genes induced by live or heat-killed *P. aeruginosa*. Transcript levels were measured in synchronized young adult animals feeding on heat-killed *E. coli* or *P. aeruginosa*, or infected with live *P. aeruginosa* PA14 for 4 h. In all cases, data are the means of two biological replicates, each replicate measured in duplicate and normalized to a control gene, expressed as the ratio of the corresponding bacteria*-*induced levels and the basal *E. coli* levels. Error bars are SEM.

Up-regulation of the 10 biomarker genes could be a result of cell damage caused by *S. aureus*, or of microbial detection independent of the inflicted damage. To discriminate between these two scenarios, we measured gene induction during exposure to live *S. aureus*, which causes pathogenesis, or heat-killed *S. aureus*, which does not (Figures 3G–I and S4C, E). Unexpectedly, all 10 biomarker genes were induced at least equally well on heat-killed *S. aureus* as on live *S. aureus* (Figure 6A). This result suggested that the ten biomarker genes form part of a host response against microbe-derived molecules, possibly pathogen-associated molecular patterns (PAMPs) [3], and not of a damage response.

Bacterial cell-wall components, such as LPS and flagellin in Gram-negative bacteria and peptidoglycan in both Gram-negatives and Gram-positives, are common PAMPs in many systems [57]. To test whether Gram-positive cell wall components in general were able to trigger the same *S. aureus-*induced response, we assayed gene induction in animals feeding on non-pathogenic Gram-positive bacterium *Bacillus subtilis* compared with *E. coli-*fed controls. Remarkably, of the ten biomarker genes, only *fmo-2*, *ilys-3*, and *Y65B4BR.1* were induced by *B. subtilis*, albeit at much lower levels than with *S. aureus* (Figure 6B). The remaining 7 genes either were not induced or were repressed by *B. subtilis*. These results suggest that PAMPs other than shared Gram-positive cell wall molecules may be molecular triggers for the *S. aureus-*induced host response in *C. elegans* (see Discussion).

How is *S. aureus* detected? In fruit flies and mammals, Toll-like receptors (TLR) are involved in PAMP detection. *C. elegans* has a single gene encoding a TLR, *tol-1*, which has been shown to be important for avoidance responses to *Serratia marcescens* and for full induction of antimicrobial peptide *abf-2* in response to *Salmonella enterica* [58], [59]. However, *tol-1* was not required for the induction of any of the 10 *S. aureus*–induced biomarker genes (Figure 6C). Furthermore, *tol-1* mutants were not more susceptible to *S. aureus-*mediated killing than wild type (Figure S10). One caveat is that the *tol-1(nr2033)* allele used, a deletion that eliminates the cytoplasmic TIR domain necessary for signaling [60], is viable, thus considered a partial loss of function for viability but a null allele for immune signaling [58]. These results show that *tol-1* is most likely not required for the *C. elegans* host response to *S. aureus* and suggest that alternative mechanisms may exist for PAMP detection.

Since *mpk-1*/ERK is important for the rectal epithelial cell swelling response to *M. nematophilum* [47] and for the swelling response to *S. aureus* (Figure S4K, L), we wondered whether *mpk-1* might also be important for the intestinal response to *S. aureus*. However, *mpk-1* animals exhibited no measurable defect in the induction of the biomarker genes relative to *E. coli-*fed controls (Figure S9). Therefore, *mpk-1* is dispensable for at least part of the intestinal transcriptional response to *S. aureus*, but not the anal swelling response.

In contrast to *S. aureus*, which either alive or dead triggered the induction of 10 biomarker genes (Figure 6A), heat-killed *P. aeruginosa* did not trigger the induction of 10 *P. aeruginosa-*induced biomarker genes (Figure 6D). Together, these data are consistent with the idea that *C. elegans* may recognize *S. aureus* infection mainly via TLR-independent PAMP detection, whereas it may recognize *P. aeruginosa* infection via detection of either Damage-Associated Molecular Patterns (DAMPs), or unidentified heat-labile PAMPs.

### *P. aeruginosa* and *S. aureus* trigger partially overlapping host responses

**Figure 7:**
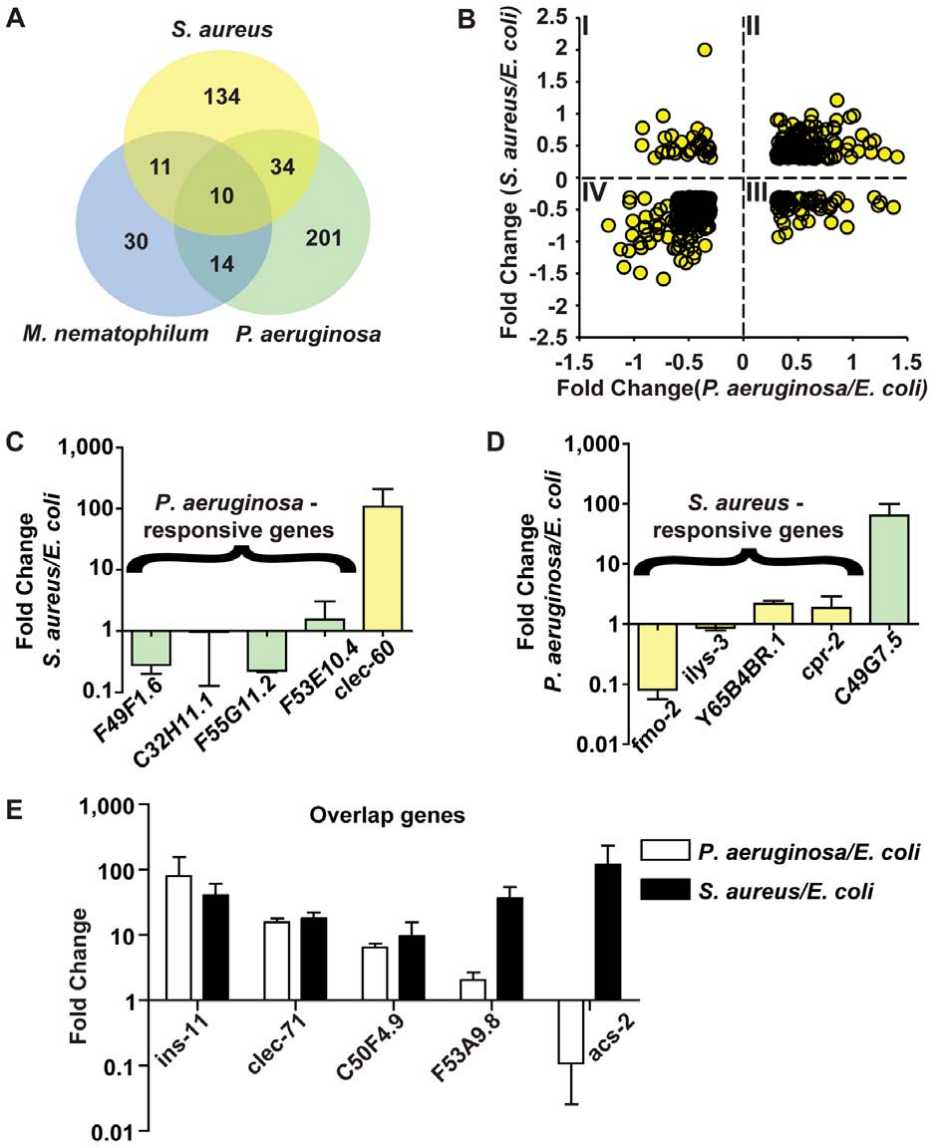
The *C. elegans* host response is comprised of pathogen-specific and -shared components. **A**. Comparison of genes induced 2-fold or higher (*p*≤0.01) upon infection with *S. aureus* for 8 h, *P. aeruginosa* for 8 h, and *M. nematophilum* for 6 h. Gene identities are presented in Table 3. **B**. Quadrant analysis of genes with expression changes both during *S. aureus* and *P. aeruginosa* infection. X axis, Fold Change for each gene during *P. aeruginosa* infection (log_10_ values). Y axis, Fold Change for each gene during *S. aureus* infection (log_10_ values). **C**. qRT-PCR analysis of *P. aeruginosa*–induced genes during *S. aureus* infection. **D**. qRT-PCR analysis of *S. aureus*–induced genes during *P. aeruginosa* infection. Transcript levels were measured in synchronized young adult wild-type animals feeding on non-pathogenic *E. coli* OP50 or infected with pathogen for 8 h (*S. aureus* NCTC8325) or 4 h (*P. aeruginosa* PA14). Results are the average of three biological replicates, each replicate measured in duplicate and normalized to a control gene, expressed as the ratio of the corresponding *P. aeruginosa -*induced levels and the basal *E. coli* levels. **E**. qRT-PCR analysis of overlap genes during *S. aureus* or *P. aeruginosa* infection. Transcript levels were measured in synchronized young adult wild-type animals feeding on non-pathogenic *E. coli* or infected for 8 h or 4 h, respectively (on *P. aeruginosa*, these genes were predicted to change by 4 h as well as 8 h). Results are the average of three biological replicates, each replicate measured in duplicate and normalized to a control gene, expressed as the ratio of the corresponding pathogen*-*induced levels and the basal *E. coli* levels. Previous microarray analysis wrongly predicted *acs-2* to be induced by *P. aeruginosa*, the only example of this kind that we have found.

Host responses to pathogenic attack in plants and animals are remarkably pathogen-specific. In *C. elegans*, pathogen-specific gene induction has been observed at late times of infection (*i.e.*, 24 h), when damage and necrosis are apparent [50]. To determine whether pathogen-elicited gene induction at earlier times (*i.e.*, 8 h) is also pathogen-specific, we compared the host response to *S. aureus* with our previously published study of the early host response to *P. aeruginosa* [44]. Of 186 genes induced by *S. aureus* and 259 genes induced by *P. aeruginosa*, 44 genes were induced by both pathogens, which is more than expected by chance (Figure 7A, Table 3). We validated the results of the microarray comparison using qRT-PCR; four genes predicted to be specifically induced by *P. aeruginosa* were either unaffected or repressed by *S. aureus* (Figure 7C), and four genes predicted to be induced by *S. aureus* were either unaffected or repressed by *P. aeruginosa* (Figure 7D). In contrast, four of five predicted overlap genes showed increased expression with both *S. aureus* and *P. aeruginosa* (Figure 7E). This suggests that the early host response to pathogen attack involves activation of a “pan-pathogen” response against a broad spectrum of pathogens, as well as a more tailored response that is optimized for the defense against the specific class of pathogens that is causing the infection.

**Table 3:** Pathogen specificity of the response to infection.

a. *S. aureus* AND *P. aeruginosa* AND *M. nematophilum*		
Name	Description	Signal Peptide
*acs-2*	Long-chain-fatty-acid-CoA ligase	+
*C15A11.7*	Amine oxidase, similar to tetracycline resistance protein TetB	−
*C50F4.9*	Contains similarity to *Phoneutria reidyi* Neurotoxin PRTx3–7	+
*ech-9*	Enoyl-CoA hydratase	−
*F52F10.3*	Acyltransferase 3 family	−
*F53A9.8*	His-rich short polypeptide	−
*glc-1*	Alpha subunit of a glutamate-gated chloride channel	+
*H02F09.3*	Contains similarity to *Staphylococcus haemolyticus* Serine-rich adhesin for platelets precursor.; SW:Q4L9P0	+
*K05F1.10*	Contains similarity to Interpro domains IPR002919 (Protease inhibitor I8, cysteine-rich trypsin inhibitor-like), IPR013032 (EGF-like region)	+
*ZC443.3*	Contains similarity to *Saccharomyces cerevisiae* essential protein involved in intracellular protein transport, coiled-coil protein necessary for transport from ER to Golgi; required for assembly of the ER-to-Golgi SNARE complex; SGD:YDL058W	−

b. *S. aureus* AND *M. nematophilum* NOT *P. aeruginosa*		
Name	Description	Signal Peptide
*C07A9.8*	Putative membrane protein, Bestrophin	−
*ilys-2*	Invertebrate lysozyme	+
*clec-60*	C-type lectin, von Willebrand factor, type A	+
*clec-61*	C-type lectin, von Willebrand factor, type A	+
*clec-62*	C-type lectin, von Willebrand factor, type A	+
*clec-70*	C-type lectin	+
*F09F7.6*		−
*F54F3.3*	Lipase	+
*R07C12.1*		−
*sodh-1*	Zinc-containing alcohol dehydrogenase	−
*T22F3.11*	Permease, major facilitator superfamily transporter	+

c. *S. aureus* AND *P. aeruginosa* NOT *M. nematophilum*		
Name	Description	Signal Peptide
*C18A11.1*	Short (119 aa)	−
*C23G10.11*	Short (63 aa)	+
*C29F7.2*	DUF1679; Predicted small molecule kinase	−
*C34C6.7*		−
*C46H11.2*	Flavin-containing monooxygenase	−
*C50F7.5*	Pro-rich	+
*clec-71*	C-type lectin	+
*cpt-4*	Carnitine O-acyltransferase CPTI	−
*cyp-34A9*	Cytochrome P450 CYP2 subfamily	+
*D1086.5*		+
*dod-22*	CUB-like domain	+
*F16C3.2*		+
*F21E9.3*	Transthyretin-like family	+
*F21F8.4*	Aspartyl protease	+
*F41G4.8*		+
*F45D3.4*		+
*F46B3.1*	Metridin-like ShK toxin	+
*F49H6.13*	DUF1164	+
*F53A9.1*	Short His/Gly rich (77 aa)	−
*F53A9.2*	Short His/Gly rich (83 aa)	−
*F53A9.6*	Short His/Gly rich (86 aa)	−
*far-7*	Nematode fatty acid retinoid binding protein	−
*gbh-2*	Gamma-butyrobetaine,2-oxoglutarate dioxygenase	−
*hsp-12.3*	Alpha-B-crystallin	−
*ins-11*	Insulin-like peptide of the insulin superfamily of proteins	+
*M60.2*	Placental protein 11	+
*mpk-2*	Mitogen-activated protein kinase	−
*srsx-34*	Permease, major facilitator superfamily	−
*T01C3.4*	Triacylglycerol lipase	+
*T04F8.7*		−
*ugt-18*	UDP-glucoronosyl/UDP-glucosyl transferase	+
*Y40B10A.6*	O-methyltransferase, family 3	−
*Y43C5A.3*	Short, basic Gly-rich (108 aa); contains similarity to *Ixodes scapularis* Putative secreted salivary gland peptide.; TR:Q5Q982	+
*Y60C6A.1*	Protein of unknown function CX	+

d. *M. nematophilum* AND *P. aeruginosa* NOT *S. aureus*		
*Name*	Description	Signal Peptide
*B0024.4*	DUF274	+
*C17H12.8*	CUB-like domain	+
*C31A11.5*	Predicted acyltransferase	+
*C49C8.5*	Ficolin and related extracellular proteins	+
*clec-67*	C-type lectin	+
*clec-69*	C-type lectin	+
*clec-86*	C-type lectin	+
*F09B9.1*	Predicted acyltransferase	+
*F35E12.5*	CUB-like domain	+
*F52F10.3*	Predicted acyltransferase	−
*M28.8*		+
*Y41D4B.16*	DUF274	+
*Y46C8AL.2*	C-type lectin, Proline-rich region	+
*ZK896.5*	CUB-like domain	+

Partially overlapping responses between *S. aureus, P. aeruginosa* and *M. nematophilum* (**a**), *S. aureus* and *M. nematophilum* (**b**), *S. aureus* and *P. aeruginosa* (**c**), or *M. nematophilum* and *P. aeruginosa* (**d**). Genes encoding products with signal peptides suggestive of secretion are indicated.

To further test the hypothesis of the existence of pathogen-shared and -specific components of the host response, we compared the genes differentially affected by *S. aureus* infection with previously published profiling of *C. elegans* infected with *M. nematophilum* [61]. The comparison of microarray studies independently performed in these separate laboratories may underestimate overlapping gene sets due to the use of different infection, RNA extraction, and data processing methods. Nonetheless, we found a relatively high degree of overlap between the responses to *S. aureus* and *M. nematophilum* (21 genes out of 186); 10 genes were induced by *S. aureus, M. nematophilum*, and *P. aeruginosa* (Figure 7A, Table 3). In contrast, 44 (68%) out of 65 genes induced by *M. nematophilum* were not induced by *S. aureus*. These data further support the existence of a core, shared response and a specific, tailored response.

GO annotations of genes affected by *S. aureus* or *P. aeruginosa* also exhibit a degree of specificity. Whereas the *S. aureus-*induced host response includes many sugar binding proteins, the *P. aeruginosa-*induced response is not characterized by any particular over-represented GO annotation (Table S7). Additionally, whereas the repressed response to *S. aureus* consists of many transporters and cuticle components, the repressed response to *P. aeruginosa* is mostly represented by metabolic pathways (Table S7). Interestingly, both repressed responses included basement membrane genes (N = 5, *p* = 1.4E-6 for *P. aeruginosa*), suggesting host growth suppression by both types of infection.

To investigate whether there were correlated or anti-correlated components of the responses to *S. aureus* and *P. aeruginosa*, we focused on genes whose expression changed both during infection with *S. aureus* and infection with *P. aeruginosa*. We broke down the two responses by plotting genes whose expression changed more than 2-fold with *S. aureus* (Y axis in Figure 7B) and with *P. aeruginosa* (X axis in Figure 7B). Genes whose expression changed only during infection with one of the two pathogens were thus not included. This method defined four quadrants: I) Genes induced by *S. aureus* and repressed by *P. aeruginosa*; II) Genes induced by *S. aureus* and *P. aeruginosa*; III) Genes repressed by *S. aureus* and induced by *P. aeruginosa*; and IV) Genes repressed by *S. aureus* and *P. aeruginosa* (Figure 7B). NextBio biogroup representation analysis (see Materials and Methods) on each quadrant failed to detect any over-represented biogroup in Quadrants I and III. However, in Quadrant II (genes up-regulated by both pathogens) several biogroups were over-represented, *e.g.* genes involved in detoxification, iron sequestration, and energy generation (Table S2a, b). Likewise, biogroups over-represented in Quadrant IV (down-regulated by both pathogens) included transporters, cuticle components, and fatty acid (FA) β-oxidation (Table S2a, b). The common repression of FA β-oxidation is not consistent with a starvation response, where β-oxidation is induced [56]. Furthermore, only 3 of 9 fasting repressed genes (*lbp-8*, *acdh-1*, and *F08A8.2*) [56] were repressed during *P. aeruginosa* infection [44]. Similarly to *S. aureus*, the fasting-repressed gene *hacd-1* was induced during *P. aeruginosa* infection [44]. Together, these observations show the distinct nature of the host response to distinct pathogens, and highlights metabolic components of early host responses to infection.

Because overlapping gene expression changes measured by qRT-PCR do not necessarily imply the involvement of the same tissues during infection with different pathogens, we further investigated the expression of *clec-60::GFP* (up-regulated by *S. aureus* and *M. nematophilum*, Figure S11A, B, C, D) and *F53A9.8::GFP* (up-regulated by *S. aureus, M. nematophilum*, and *P. aeruginosa*, Figure S11E, F, G, H). *clec-60::GFP* was induced by *M. nematophilum* and by *S. aureus* in the intestine (Figure S11C, D), and down-regulated by *P. aeruginosa* (Figure S11B) compared with non-pathogenic *E. coli* (Figure S11A). Likewise, *F53A9.8::GFP* was induced by all three pathogens in the intestine (Figure S11F, G, H) compared with *E. coli* controls (Figure S11E). These data suggest that components of the host response are induced in the intestine by distinct pathogens.

### Genes induced by *S. aureus* influence host survival

**Figure 8:**
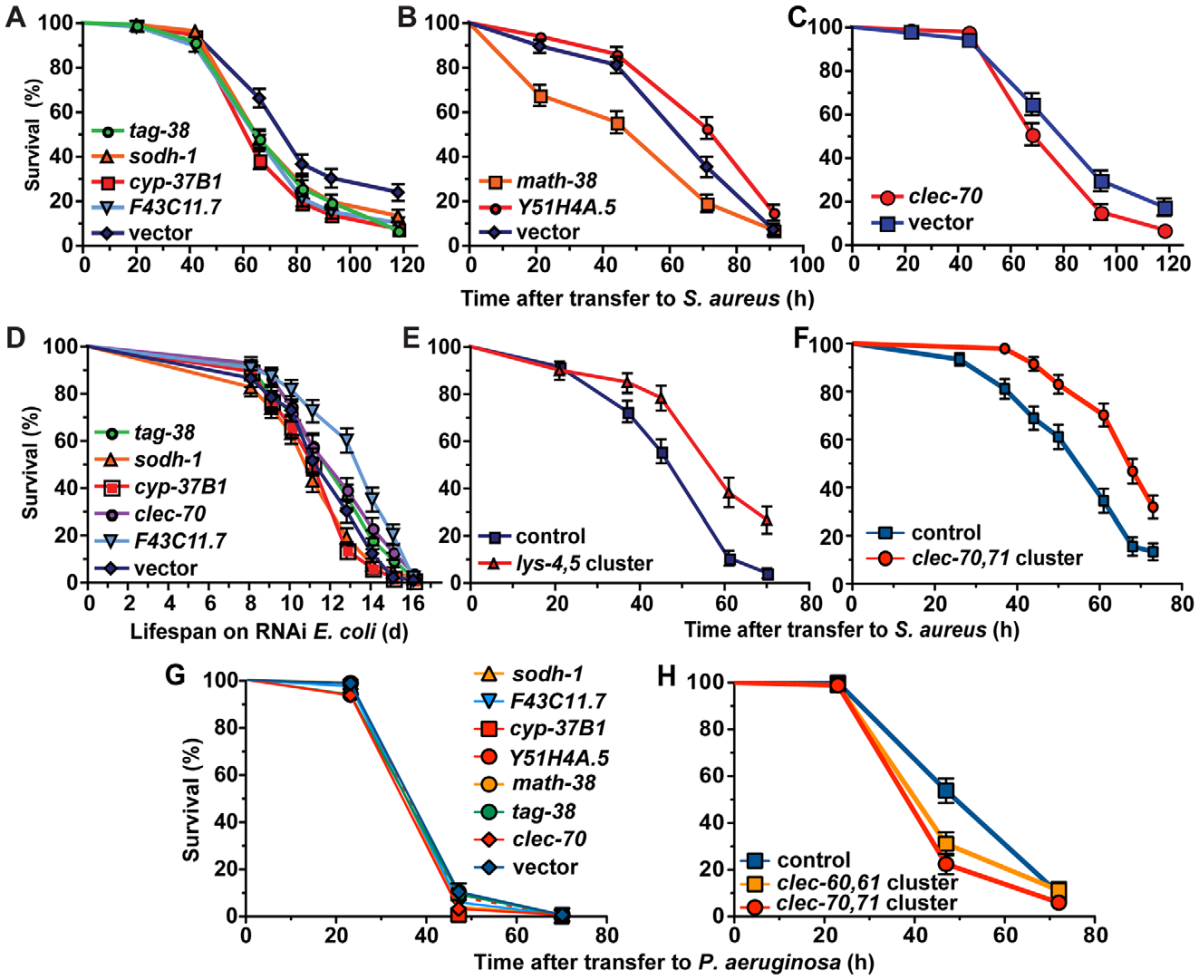
Host response genes are biologically relevant to host survival during *S. aureus* infection. **A, B, C**. After RNAi knockdown of *S. aureus-*induced genes, animals were transferred to *S. aureus* NCTC8325 infection plates. Survival statistics: *vector* in **A** (MS = 82 h; N = 125), *tag-38* (MS = 66 h, N = 142, *p* = 0.0002), *sodh-1* (MS = 66 h; N = 142; *p* = 0.009), *cyp-37B1* (MS = 66 h; N = 145; *p*<0.0001), *F43C11.7* (MS = 66 h; N = 147; *p = *0.0003), *vector* in **B** (MS = 71 h; LT_50_ = 67.6 h, N = 99/8), *math-38* (LT_50_ = 48.6 h; N = 92/7; *p = *0.0012), *Y51H4A.5* (MS = 91 h; N = 100; *p = *0.0122); *vector* in **C** (LT_50_ = 68.1 h; N = 91), *clec-70* (LT_50_ = 67.9 h; N = 98; *p = *0.0145). **D**. Lifespan of *E. coli-*fed animals. Lifespan statistics: *vector* (MS = 12.8 d; N = 89), *tag-38* (MS = 12.8 d, N = 93, *p = *0.1708), *sodh-1* (MS = 11.1 d; N = 104; *p = *0.1887), *cyp-37B1* (MS = 12.8 d; N = 95; *p* = 0.1473), *clec-70* (MS = 12.8 d; N = 99; *p = *0.0354), *F43C11.7* (MS = 14.08 d; N = 88; *p<*0.0001). **E, F**. Overexpression of host response genes enhances host survival during *S. aureus* infection. **E**. Animals carrying *lys-4,5* cluster transgenes survived longer (LT_50_ = 54.1 h; N = 60; *p*<0.0001) during *S. aureus* infection than control animals bearing transgenes composed of coinjection marker and *clec-60::GFP* promoter fusion (LT_50_ = 40.6 h; N = 91/4). **F**. Animals carrying an integrated array containing a cluster of *clec-70,71* (MS = 68 h; N = 94; *p*<0.0001) were more resistant to *S. aureus* mediated killing than control animals bearing *rol-6* coinjection marker alone (MS = 61 h; N = 90). **G**. RNAi knockdown of *S. aureus* induced genes did not result in enhanced susceptibility to *P. aeruginosa*. Survival statistics: *vector* (MS = 47 h, N = 80/9); *sodh-1* (MS = 47 h, N = 84/8, *p* = 0.2476); *F43C11.7* (MS = 47 h, N = 83/9, *p* = 0.2592); *cyp-37B1* (MS = 47 h, N = 92/11, *p* = 0.0201); *Y51H4A.5* (MS = 47 h, N = 83/5, *p* = 0.6712); *math-38* (MS = 47 h, N = 85/13, *p* = 0.9919); *tag-38* (MS = 47 h, N = 90/10, *p* = 0.3373); *clec-70* (MS = 47 h, N = 84/8, *p* = 0.0184). **H**. Overexpression of *S. aureus* induced C-type lectin genes resulted in mild enhanced susceptibility to *P. aeruginosa. clec-60::GFP,myo-2::mCherry* control (MS = 72 h; N = 98/20), *clec-60,61,myo-2::mCherry* (MS = 47 h; N = 94/16; *p* = 0.0408), *clec-70,71, myo-2::mCherry* (MS = 47 h; N = 92/12; *p* = 0.0002).

To determine whether *S. aureus*-induced genes have protective functions in host defense, we performed whole-animal RNAi knockdown of 42 of the 46 most highly up-regulated genes (Table 1), and identified 6 genes [*tag-38* (Glutamate decarboxylase/sphingosine phosphate lyase), *sodh-1* (sorbitol dehydrogenase), *cyp-37B1* (Cytochrome P450), *F43C11.7* (F-box containing protein), *math-38* (MATH domain-containing signaling protein), and *clec-70* (secreted CTL)] whose decreased expression caused enhanced susceptibility to killing by *S. aureus* (Figure 8A,B, C), but not *P. aeruginosa* (Figure 8G). We also identified one gene,*Y51H4A.5* (a putative intracellular lipase) whose decreased expression caused slightly enhanced resistance to *S. aureus* killing, suggesting that its expression is detrimental to the host, or that it functions as a repressor of host defense (Figure 8B). Importantly, the lifespan of animals continuously fed dsRNA-expressing, non-pathogenic *E. coli* was near wild type, except for *F43C11.7*, which actually caused increased lifespan (Figure 8D). These data show that *S. aureus-*induced genes have important functions in pathogen-specific host defense, and that the enhanced susceptibility to *S. aureus* mediated by RNAi knockdown is not due to a non-specific decrease in viability. Although the molecular identities of these genes offer clues to their potential functions, the elucidation of their exact mechanisms of action will require further study.

Potential immune effectors identified in our analysis include antimicrobial peptides, lysozymes (enzymes that degrade peptidoglycan in the bacterial cell wall), and CTLs [62]. To test whether lysozymes or CTLs induced by *S. aureus* can confer resistance to *S. aureus-*mediated killing when expressed to higher levels than in the wild type, we constructed transgenic *C. elegans* carrying multiple copies of *lys-4* and *lys-5*, *clec-60* and *clec-61*, or *clec-70* and *clec-71* (each is a pair of genes that are adjacent to each other in the genome). Transgenic animals carrying the *lys-4,5 or clec-60,61* clusters survived significantly longer than transgenic control animals (Figure 8E; Figure S12). Transgenic animals carrying the *clec-70,71* cluster extrachromosomally did not exhibit enhanced resistance (not shown); however, three independent spontaneous integrant lines did (Figure 8F). Interestingly, strains with multiple copies of either cluster of C-type lectin genes exhibited enhanced susceptibility to *P. aeruginosa-*mediated killing (Figure 8H). Collectively, the data suggest that pathogen-induced intestinal expression of epithelial detoxifying and antimicrobial proteins is an important and pathogen-specific mechanism of *C. elegans* host defense against *S. aureus* infection.

## Discussion

Among the bacteria that cause intestinal infections in *C. elegans*, the best-studied is *P. aeruginosa*, a Gram-negative human pathogen. Despite many advances in understanding *P. aeruginosa-C. elegans* interactions, little was known about the morphological and cell biological consequences of infection *in vivo*. This report provides unprecedented high-resolution description of bacterial intestinal infections of clinical relevance in *C. elegans*, with emphasis on the comparative cytopathology of infection by *P. aeruginosa* and *S. aureus. P. aeruginosa* and *S. aureus* cause markedly different symptoms in *C. elegans*.

During infection with *P. aeruginosa*, we observed marked intestinal distention, extracellular matrix accumulation in the intestinal lumen, extracellular material accumulation on the apical surface, enlargement of the rough endoplasmic reticulum (RER), and abnormal autophagy in the host intestinal cells. The intestinal distention is likely a result of loss of cytoplasmic volume of the intestinal cells; the identity of the extracellular material is currently unknown. One possibility is that it is a biofilm matrix produced by *P. aeruginosa*, perhaps as a defensive mechanism. Alternatively, it could be produced by the host in response to *P. aeruginosa*, but not *S. aureus*. Further study is required to elucidate the origin of this substance. The cause of abnormal autophagy is also unknown; however, the aberrant structures we observed have also been described in *unc-52* mutant animals, which are defective in the early steps of autophagosome assembly [63]. Thus, it is possible that *P. aeruginosa* infection causes autophagy arrest. It is tempting to speculate that this may represent a virulence mechanism deployed by *P. aeruginosa* to evade autophagic clearance of intracellular bacteria, an important host defense mechanism in the intestinal cells of *C. elegans* [64] and humans [65]. The intracellular invasion we observed provides a rationale for the benefit to *P. aeruginosa* of inhibiting host autophagy. *gacA* mutant *P. aeruginosa* was defective in inducing these phenotypes, indicating that GacA orchestrates virulence mechanisms related to host cell disruption in *C. elegans*. The molecular identity of these virulence mechanisms remains unknown; however, the observed putative OMVs may provide a mechanism for delivery of bacterial virulence factors to *C. elegans* intestinal cells. Perhaps the reliance on OMVs for virulence may explain why *P. aeruginosa* defective in type III secretion, an alternative mechanism of virulence factor delivery to host cells, are not defective in nematode killing [66]. OMV production is induced in *P. aeruginosa* by cellular stress [67]; therefore, the abundant OMV production we observed may indicate that *P. aeruginosa* perceives the intestinal lumen as a stressful environment, presumably as a result of defensive host factors secreted by the intestinal cells.

The molecular identity of such putative intestinal defense factors remains unknown. In previous work, we defined the early transcriptional host response to *P. aeruginosa* [68]. Among the genes that are up-regulated by *P. aeruginosa* infection, several encode putative antimicrobial factors such as ShK toxins. Whatever the molecular identity of the antibacterial factors induced during *P. aeruginosa* infection, heat-killed *P. aeruginosa* did not induce a set of 10 biomarkers of the response. This observation is consistent with our previous studies suggesting that *P. aeruginosa* virulence may be required, at least partially, for response induction [44], [69]. These results are consistent with several interpretations. It is possible that detection of virulent *P. aeruginosa* is mediated by recognition of the damage inflicted on the host cells, *e.g.*, *via* DAMP perception [70], or by recognition of PAMPs in the context of host damage, in what has come to be known as a pattern of pathogenesis [71]. Alternatively, PAMPs released only by live bacteria (PAMP-*per vitae*, or PAMP-PV) may be specifically recognized by *C. elegans* as a trigger for the response [71]. A third option is the release of PAMP-*post mortem* (PAMP-PM) upon heat inactivation of *P. aeruginosa*, which in turn could dampen the host response. PAMP-PM detection has been proposed to be a mechanism used by mammals to limit inflammatory damage to the host once the infection has been controlled [71]. Finally, detection of *P. aeruginosa* could be mediated by heat-labile signals that were destroyed during heat-inactivation in our experiments. Although further work is required to conclusively show which of these scenarios is correct, we have found that *P. aeruginosa* strains with increasing levels of virulence cause increasing levels of induction of *C. elegans* host response gene *irg-1* [69], suggesting that the extent of host cell damage determines the magnitude of the host response to the hypothetical *P. aeruginosa-*produced PAMPs or DAMPs that are detected.

During *S. aureus* infection, we observed rapid intestinal colonization, swelling of the anus, and effacement and destruction of intestinal epithelial cells, providing important mechanistic information about *S. aureus-*mediated pathogenesis of host epithelial cells *in vivo*. The molecular mechanism of cell destruction remains unknown; here we show that it is hemolysin-independent and is abrogated by heat inactivation of *S. aureus*. One explanation for this observation is that host tissue damage may be caused by the active pathogen, and not an unbridled host response. Alternatively, unknown heat-labile *S. aureus* toxins may cause cell lysis, as can occur in human cells [72]; further study is required to distinguish between these possibilities. Previous experiments using fertile animals showed that α-hemolysin Δ*hla* mutant *S. aureus* were defective in *C. elegans* killing [17]. To our surprise, the Δ*hla* Δ*hlb*, Δ*hla* Δ*hlg*, or Δ*hla* Δ*hlb* Δ*hlg* hemolysin mutants did not exhibit any killing defect in our assay using sterile animals (Figure S7). Thus, it is possible that α-hemolysin-mediated killing of *C. elegans* requires internal hatching of eggs retained inside the mother as a result of stress.

Eventually, the pathogenic process results in internal tissue degradation and nematode death. These steps recapitulate key features of *S. aureus* infection in mammals both *in vivo* and *in vitro, e.g.* enterocyte effacement during intestinal infection, and cell lysis [37], [38], [39], [40], [41], [42], [43]. Thus, we propose that the *C. elegans-S. aureus* model has significant relevance to the study of conserved virulence mechanisms used by *S. aureus* to evade host epithelial defenses and attack host epithelial cells in general.

Transcriptional profiling showed that *S. aureus* infection elicits changes in expression of a minor fraction of the total genome of ∼22,000 genes, indicating high specificity. This early response does not require live bacteria, suggesting that it involves detection of *S. aureus per se* (perhaps through PAMP perception) as opposed to indirectly through host cell damage (through DAMPs) [70]. Whatever the relevant PAMPs are, it is clear that they are not shared between *S. aureus* and *B. subtilis*, also a Gram-positive bacterium. For example, the peptidoglycan differs greatly between these two species [73], and could potentially be differentially sensed by *C. elegans*. Other possibilities include differential detection of surface-expressed lipoteichoic acids or differentially expressed surface proteins. Further work is required to elucidate the nature of such signal(s).

Our results provide insight into the cellular biological effects of pathogenic infection on the epithelial barrier *in vivo*, as well as the early defense mechanisms deployed by *C. elegans* to fend off attack. The affected genes represent evolutionarily conserved categories relevant to human innate immunity. To gain insight into evolutionarily conserved effector mechanisms of host defense, we compared the genes up-regulated in *C. elegans* with previously published data using human neutrophils, which are important effector cells in human innate immunity [74]. When grouped by molecular function, we found some overlapping functional classes during the *C. elegans* and human neutrophil responses to *S. aureus* infection (Table S4). These classes included detoxification factors [*e.g.* transporters, UDP-glucuronosyltransferases (UGTs), cytochrome P450s, GSTs, and flavin-containing monooxygenases (FMOs), [75]], antimicrobial effectors (*e.g.* CTLs, peptidases, and proteases), galectins [76], and signaling components including EGF-like domain containing proteins, Cdc42 guanyl nucleotide exchange factors (GEFs), F-box proteins, mitogen-activated protein kinases (MAPKs), and Leucine-rich repeat domain (LRR) containing proteins. This observation shows that the *C. elegans* host response to infection shares important components with the human cellular host response, and suggests that human innate responses have ancient components that are conserved across phylogeny. Indeed, it is thought that modern vertebrate innate immunity represents an accretion of ancient invertebrate innate defenses [77].

Comparative genomics identified pathogen-specific as well as pathogen-shared components of the host response. This observation, consistent with similar ones recently made by others using different approaches [50], [78], [79], illustrates how diverse pathogens affect distinct aspects of host physiology as reflected in the distinct nature of the host responses. We also found overlap in gene expression patterns among the responses to three different pathogens, *S. aureus* (intestinal infection, Gram-positive), *M. nematophilum* (cuticular infection, Gram-positive), and *P. aeruginosa* (intestinal infection, Gram-negative), defining a core induced response that involves intracellular detoxification, iron sequestration, and sugar binding. In addition to a common set of up-regulated genes, we observed a repressed core response common to *S. aureus* and *P. aeruginosa* that involves anion transport, growth-related genes, lipid- and alcohol-metabolic genes, and acyl-CoA dehydrogenases. The fact that metabolic regulation is a major component of the *C. elegans* host response to bacterial pathogens provides a rationale to investigate metabolic changes that occur in higher organisms as a result of infection, particularly in innate defense tissues such as epithelia. Recent reports suggest a significant metabolic component during the host response in mammals as well [80], [81], [82].

The non-overlapping responses to *S. aureus* and *P. aeruginosa* may reflect the different virulence strategies of the two pathogens and/or may be a consequence of the distinct molecular composition of Gram-positive and -negative cell walls. Further studies are required to dissect the relative contribution of each factor, including the survey of additional Gram-positive and -negative infections in *C. elegans*. In a first step along those lines, we found that the Gram-positive non-pathogenic bacterium *B. subtilis* does not induce the same set of 10 biomarkers as *S. aureus*. Additionally, of 531 genes up-regulated by the Gram-positive pathogen *E. faecalis* [50], only 15 were shared with the response to *S. aureus* (Table S5a). The same report characterized the *C. elegans* late host response (*i.e.*, 24 h) to four additional pathogens, identifying a set of shared genes that defined a pathogen-shared necrotic response [50]. During *S. aureus* infection we observed up-regulation of none of 16 up-regulated shared late response genes, and down-regulation of only three of six down-regulated shared response genes (Table S5c), suggesting that the host response evolves significantly over time.

Mammalian intestinal epithelial cells directly sense and respond to bacterial stimulation [2], by inducing the expression of antimicrobial genes such as CTLs [53]. Similarly, most of the early *C. elegans* response to *S. aureus* occurs in the epithelial cells of the intestine. In *Drosophila*, mice, and humans, TLRs are important receptors that drive the activation of signaling cascades downstream of microbial stimulation. In *C. elegans*, however, loss of function of the sole TLR does not result in a defective immune response to *S. aureus*. Furthermore, *C. elegans* does not have an NF-kB homolog nor an inflammasome, raising the possibility that in mammals as in *C. elegans*, at least a portion of the immune response to *S. aureus* may be regulated independently of the TLR/NF-kB signaling axis [6], [51], [83]. Indeed, we previously reported that β-catenin and HOX genes are required for perception of pathogenic attack by *S. aureus* to drive the expression of epithelial host response genes [7]. Significantly, we also found that β-catenin and HOX proteins modulated NF-κB signaling in a human epithelial cell line during TLR2 stimulation, illustrating that previously unknown human innate immunity pathways can be identified using *C. elegans* [7].

We identified 6 host factors out of 42 tested whose lowered expression caused enhanced susceptibility to *S. aureus*. This corresponds to a 14% hit rate, which was much greater than expected; we had assumed that there would be significant functional redundancy among *C. elegans* immune effectors. Moreover, RNAi typically exhibits incomplete penetrance and expressivity. In addition to these 6 genes, knockdown of *Y51H4A.5* (lipase) caused mild resistance to *S. aureus* mediated killing, suggesting that *Y51H4A.5* acts to limit survival as a negative regulator of the host response or by harming the host instead of protecting it. RNAi of *F43C11.7* caused enhanced susceptibility to *S. aureus*, yet extended lifespan on non-pathogenic *E. coli*. This is an example of genes that have opposite effects on host defense and lifespan regulation, indicating that these related processes are genetically separable [84]. Increased expression of host defense genes provides a mechanistic explanation of *C. elegans* defenses, as we found that animals carrying multiple copies of three genomic clusters of lysozymes or CTLs exhibited enhanced resistance to *S. aureus*. Lysozymes are well-known, evolutionarily ancient antibacterial effector molecules that degrade peptidoglycan and are also produced in human intestinal epithelial cells [1]. Recent studies have shown that some vertebrate CTLs, including human HIP/PAP, have direct bactericidal activity [53]. *clec-70* and *clec-71* share similar domain architectures with HIP, and *clec-60* and *clec-61* share similarities with arthropod receptor CTLs, suggesting that they may function respectively as antimicrobials or receptors in the *C. elegans* host response [85], [86], [87]. Collectively, these observations show that the large number of pathogen*-*response genes contribute cumulative, incremental defense functions to host survival.

It is interesting that elements of the *C. elegans* immune response enhance host survival during infection with *S. aureus* (a human pathogen), supporting the notion that pathogen detection and response, as well as mechanisms of bacterial pathogenesis, share conserved features among distantly related hosts or microbes, respectively. It has been proposed that pathogens have experienced stepwise additions of virulence factors, as they evolved to survive different host antimicrobial responses, and to colonize new niches [12]. Our studies of the *C. elegans-S. aureus* system may thus probe an early step in the evolution of *S. aureus* as a pathogen and its interaction with prototypical metazoan epithelial cells. In humans, unknown host and bacterial factors determine whether *S. aureus* will become an innocuous member of the normal microbiota, or whether it will switch to a more virulent state and become a serious pathogen [23]. In this light, studies of the *C. elegans* intestinal epithelial response to *S. aureus* provide a unique starting point to identify previously unknown signaling pathways and molecular mechanisms of host immune response to bacterial virulence. Understanding how *S. aureus* disrupts host defense and causes host damage and death is critical to identifying new therapeutic targets to treat infectious disease.

## Materials and Methods

### Strains

*C. elegans* was grown on nematode-growth media (NGM) plates seeded with *E. coli* OP50-1 at 15–20°C according to standard procedures [88]. *C. elegans* strains used in this study are detailed in Table S6a. Bacterial strains are detailed in Table S6b.

### Electron microscopy

Wild type N2 Bristol animals were synchronized by hypochlorite treatment and L1 arrest and incubated on NGM plates seeded with *E. coli* OP50-1. Late L4 animals were collected and plated on 15 cm TSA plates seeded with live *S. aureus* NCTC8325 or heat-killed NCTC8325, and parallel NGM plates seeded with OP50-1. After 12, 24, and 36 h incubation at 25°C, animals were collected and incubated in fixation buffer (2.5% glutaraldehyde, 1.0% paraformaldehyde in 0.05 M sodium cacodylate buffer, pH 7.4 plus 3.0% sucrose). During the initiation of fixation, animals were cut in half with a surgical blade in a drop of fixative under a dissecting microscope, fixed overnight at 4°C, rinsed in 0.1 M cacodylate buffer, post-fixed in 1.0% osmium tetroxide 0.1 M cacodylate buffer, rinsed in buffer and water, and stained *en bloc* in 2% aqueous uranyl acetate. After rinsing in water, animals were embedded in 2% agarose in phosphate buffer saline, dehydrated through a graded series of ethanol washes to 100%, then 100% propylene oxide, and finally 1∶1 propylene oxide:EPON overnight. Blocks were infiltrated in 100% EPON and then embedded in fresh EPON overnight at 60°C. Thin sections were cut on a Reichert Ultracut E ultramicrotome and collected on formvar-coated gold grids. Sections were post-stained with uranyl acetate and lead citrate and viewed using a JEOL 1011 transmission electron microscope at 80 kV with an AMT digital imaging system (Advanced Microscopy Techniques, Danvers, MA). For each observation, whenever possible at least 10 cross-sections were evaluated, and representative images were chosen.

### Microarray analysis

#### *C. elegans* growth and infection

*fer-15(b26)ts;fem-1(hc17)* animals were synchronized by hypochlorite treatment and L1 arrest [88]. Arrested L1 larvae were placed onto NGM plates seeded with OP50 and grown at the restrictive temperature (25°C) in order to obtain sterile adults. Young adult animals were transferred to slow-killing plates (NGM agar containing 0.35% peptone), seeded with OP50, or tryptic soy agar plates (TSA, see below) seeded with RN6390. Animals were harvested at 8 h after transfer. Three independent replicates of each treatment were isolated.

#### RNA isolation

Total RNA was extracted using TRI Reagent (Molecular Research Center, http://www.mrcgene.com) according to the manufacturer's instructions, followed by purification on RNeasy columns (Qiagen, http://www1.qiagen.com).

#### Microarray target preparation and hybridization for Affymetrix GeneChips

RNA samples were prepared and hybridized to Affymetrix full-genome GeneChips for *C. elegans* at the Harvard Medical School Biopolymer Facility, according to instructions from Affymetrix (http://www.affymetrix.com). Briefly, 5 µg of total RNA was reverse transcribed using an oligo dT-T7 primer and Superscript II reverse transcriptase, followed by second-strand cDNA synthesis. The double-stranded cDNA was then purified using a DNA purification kit (Qiagen), and used as the template for *in vitro* transcription using T7 RNA polymerase and biotinylated nucleotides. The resulting cRNA was fragmented and hybridized onto *C. elegans* Affymetrix GeneChips as previously described [44].

#### Microarray analysis for *S. aureus* studies

Affymetrix .cel files were uploaded into the Resolver Gene Expression Data Analysis System, version 5.1 (Rosetta Inpharmatics, http://www.rii.com) at the Harvard Center for Genomic Research for analysis. For each condition, three replicate microarrays were normalized and analyzed using the Resolver intensity error model for single color chips [89]. The two conditions were then compared in Resolver to determine fold change for each probe set and a *p*-value, using a modified *t* test. Genes with a 2-fold or greater fold change and a *p*-value <0.01 were considered differentially expressed. Differentially expressed probe sets were compared for *S. aureus* infection (this study), *P. aeruginosa* infection [44], and *M. nematophilum* infection [90] using Resolver.

#### Significance of over-representation analysis

Analysis of over-representation of GO annotation categories was performed using FuncAssociate (http://llama.med.harvard.edu/cgi/func/funcassociate). GO Bioset analysis was performed using NEXTBIO (www.nextbio.com) [91]. For greater inclusivity, gene expression changes greater than 1 were included in the NEXTBIO analysis, as suggested by NEXTBIO.

### Infection and lifespan assays

All assays were conducted at 25°C, 65% relative humidity. Animals were scored as alive or dead by gentle prodding with a platinum wire. Kaplan-Meier statistical analyses were performed using the software Prism (GraphPad, http://www.graphpad.com). Survival data were compared as described using the log-rank test. Data are represented as median survival (MS) or lethal time – 50 (LT_50_) when MS values were skewed by small number of timepoints, N (number of deaths/censored), and *p* value. A *p*-value <0.05 was considered significantly different from control.

#### *S. aureus* killing assays

Assays were performed as described [17]. Briefly, NCTC8325 (or mutant derivatives, as noted) was grown overnight in tryptic soy broth (TSB, BD, Sparks, MD) with 10 µg/ml nalidixic acid (Sigma). 5–10 µl of overnight cultures diluted 1∶5 in fresh TSB were seeded on 35 mm tryptic soy agar (TSA, BD, Sparks, MD) plates with 10 µg/ml nalidixic acid. For accumulation experiments using GFP-*S. aureus*, plates were supplemented with 10 µg/ml chloramphenicol (Sigma) for plasmid maintenance. A total of 25–35 L4 stage hermaphrodites were transferred to each of three replicate plates per strain. Animals that died as a consequence of a bursting vulva or crawled off the agar were censored. Experiments were performed at least twice. For heat-killing of *S. aureus*, fresh overnight cultures of NCTC8325 were concentrated 10-fold and incubated at 95°C for 45 min. Following heat treatment, no live cells could be detected by plating undiluted cultures on TSA plates.

#### *P. aeruginosa* slow-killing assays

Briefly, PA14 was cultured in Luria broth (LB), seeded on slow-killing plates and incubated first for 24 h at 37°C and then for 24 h at 25°C. A total of 25–35 L4 stage hermaphrodites were transferred to each of three replicate plates per strain. Experiments were performed at least twice.

#### *M. nematophilum* infection assays

Assays were performed as described [92]. Briefly, OP50 and CBX102 were cultured overnight in LB, mixed at 1∶10 ratio of CBX102:OP50 and plated on NGM. A total of 25–35 L4 stage hermaphrodites were transferred to each of three replicate plates per strain. Assays were conducted at 25°C. For Dar quantification, animals were analyzed directly on infection plates, in triplicate.

#### Lifespan assays

Briefly, RNAi plates seeded with dsRNA-expressing *E. coli* HT115 were used. Approximately 100 synchronized *eri-1(mg366)* L1 larvae were added to each of 3 plates, and incubated for 24 h at 15°C followed by 24 h at 25°C, which causes animal sterility. 35–50 late L4 stage animals were added to each of 3 fresh RNAi plates seeded with dsRNA-expressing *E. coli* HT115, and incubated at 25°C. Lifespan is defined as the time elapsed from when animals were put on plates to when they were scored as dead. Experiments were performed at least twice. Animals that died of a bursting vulva or crawled off the agar were censored.

### Quantitative RT-PCR (qRT-PCR) analysis

Animals were treated essentially as described for killing assays described above, with the following modifications. For *S. aureus* infection assays, infected samples were compared to parallel samples feeding on *E. coli* OP50, heat-killed by 30 min incubation at 95°C, plated on the same TSA medium. All strains compared were grown in parallel. Total RNA was extracted using TRI Reagent, and reverse transcribed using the Superscript III kit (Invitrogen). cDNA was subjected to qRT-PCR analysis using SYBR green detection (BIO-RAD SYBR Green supermix) on iCycler (Bio-Rad, http://www.bio-rad.com) and RealPlus (Eppendorf, Germany) machines. Primers for qRT-PCR were designed using Primer3Plus (Massachusetts Institute of Technology, http://www.bioinformatics.nl/cgi-bin/primer3plus/primer3plus.cgi), checked for specificity against the *C. elegans* genome and tested for efficiency with a dilution series of template. Primer sequences are available upon request. All Ct values are normalized against the control gene *snb-1*, which did not vary under conditions being tested. Fold change was calculated using the Pfaffl method [93]. We found some variability in gene induction levels from experiment to experiment. The source of this variation has not been conclusively ascertained; however, we suspect it may derive from differences between batches of agar plates used for infection assays. Importantly, all experiments were repeated at least twice (biological replicates) and were internally controlled. Additionally, despite numerical variability in fold induction, all results were internally consistent.

### GFP fusions

PCR primers to amplify 1665 bp of sequence upstream of the *clec-60* start site, 3505 bp upstream of the *clec-70* start site, 1774 bp upstream of the *fmo-2* start site, and 913 bp upstream of the *ilys-3* start site were designed using the online PCR primer design tool provided by the British Columbia Genome Sciences Center (http://elegans.bcgsc.bc.ca/promoter_primers/index.html). Splicing by overlapping extension PCR (SOE-PCR) was used as described [94] to generate promoter-GFP fusion PCR fragments, which were transformed at 3 ng/ µl into wild type animals by microinjection with 40 ng/ µl of a *myo-2::NLS::mCherry* construct as coinjection marker used to identify transgenic animals (courtesy of J. Kaplan, Massachusetts General Hospital). Primer sequences are available upon request.

### GFP fusion induction

L4 animals carrying extrachromosomal arrays were transferred from NGM plates seeded with OP50-1 to *S. aureus, P. aeruginosa*, or *M. nematophilum* killing plates essentially as described above. After incubation, animals were mounted on glass slides with 2% agarose pads, anesthetized with 30 mM NaN_3_, and immediately used for imaging. Exposure times were set for the most highly expressed condition and kept constant throughout each experiment.

### RNAi knockdown

#### RNAi screen

Enhanced RNAi *eri-1(mg366)* mutants were propagated at 15°C. RNAi of selected genes was carried out in triplicate using bacterial feeding RNAi [95]. Synchronized L1 animals were transferred to RNAi plates, incubated at 15°C for 25 h and then 25°C for 24 h to induce sterility, and then transferred to NCTC8325-seeded killing assays. The screen was performed once, and positive clones that exhibited altered survival on *S. aureus* were tested at least once more. RNAi clones were obtained from the Ahringer laboratory (except *clec-70* RNAi, which was made by recombining pDONR201.clec-70 from the ORFeome library [96] with pDEST.L4440 [97]. Sequences of positive RNAi clones were confirmed.

#### *cdc-25.1* RNAi

To sterilize worms previous to use in killing assays, *cdc-25.1* RNAi was carried out by feeding L4 animals for 24 h at 15°C.

### PMN expression analysis

Gene expression microarray data files of *S. aureus* infection were obtained from Gene Expression Omnibus (GEO accession: GSE2405) and analyzed. The samples were derived from human polymorphonuclear leukocytes (PMNs) from three healthy donors using a separate HU133A GeneChip (Affymetrix) for each donor [74]. We examined the data from PMNs that were either uninfected or infected with live *S. aureus* for 9 hours. The dataset was MAS5.0-normalized and filtered by excluding probe sets with 100% ‘absent’ calls (MAS5.0 algorithm) across all samples. FDR analysis (q-value<0.005) with 1000 permutations using significance analysis of microarrays [98] was performed to identify genes that were differentially induced in *S. aureus*-infected PMNs versus uninfected controls.

### Epifluorescence microscopy

Images were acquired using a Zeiss AXIO Imager Z1 microscope with an Zeiss AxioCam HRm camera and Axiovision 4.6 (Zeiss) software. Image cropping and minimal manipulation were performed using Photoshop (Adobe).

### Gene accession information

Gene Public Name, Gene WormBase ID, Source GenBank ID, Gene CGC Name; *bar-1*, WBGene00000238, U46673, *bar-1*; *col-63*, WBGene00000639, Z81143, *col-63*; *col-98*, WBGene00000673, Z81503, *col-98*; *cpr-2*, WBGene00000782, Z81531, *cpr-2*; *egl-5*, WBGene00001174, L15201, *egl-5*; *exc-5*, WBGene00001366, Z68159, *exc-5*; *fmo-2*, WBGene00001477, Z70286, *fmo-2*; *ins-11*, WBGene00002094, U41279, *ins-11*; *lys-2*, WBGene00003091, AL021479, *lys-2*; *lys-5*, WBGene00003094, Z73427, *lys-5*; *mpk-1*, WBGene00003401, Z46937, *mpk-1*; *pmk-1*, WBGene00004055, U58752, *pmk-1*; *sod-3*, WBGene00004932, U42844, *sod-3*; *tol-1*, WBGene00006593, AF348166, *tol-1*; *unc-32*, WBGene00006768, Z11115, *unc-32*; *C32H11.1*, WBGene00007864, NM_070062.2; *C50F4.9*, WBGene00008234, Z70750; *F01D5.2*, WBGene00008493, Z81493; *acs-2*, WBGene00009221, Z81071, *acs-2*; *F55G11.2*, WBGene00010123, Z82272; *clec-60*, WBGene00014046, Z49132, *clec-60*; *clec-61*, WBGene00014047, Z49132, *clec-61*; *clec-52*, WBGene00015052, U58752, *clec-52*; *C23G10.6*, WBGene00016013, U39851; *C30G12.2*, WBGene00016274, U21319; *ilys-3*, WBGene00016670, AF067611, *ilys-3*; *C49G7.5*, WBGene00016783, AF016418; *acdh-1*, WBGene00016943, AC006625, *acdh-1*; *F49F1.6*, WBGene00018646, AF100656; *F53A9.8*, WBGene00018731, U23523; *F53E10.4*, WBGene00018760, U88177; *clec-70*, WBGene00021581, AC024785, *clec-70*; *clec-71*, WBGene00021582, AC024785, *clec-71*; *Y65B4BR.1*, WBGene00022040, AC024847.

## Supporting Information

Figure S1 *P. aeruginosa* makes putative outer membrane vesicles (OMVs), disrupts the brush border, and penetrates the epithelial barrier.
**A–D**. TEM micrographs of *P. aeruginosa*-infected animals after 48 h infection. Scale bars, 0.5 µm. **A**. Detail of intestinal lumen filled with OMVs and bacterial cells (b), and brush border (mv) coated with extracellular material (em). Note disruption of the microvilli (black asterisk) and OMV shedding from the bacterial cells (black arrowheads). At this time point the terminal web (tw) appears whole. **B**. High magnification TEM showing apparent OMV shedding off bacterial cells (b, indicated with black arrowheads). **C**. Example of distal dissemination of *P. aeruginosa.* A bacterial cell (b) is shown between the body-wall muscle (bwm) and the cuticle (cu), which is the exoskeleton of the animal. **D**. Detail of animal infected with *gacA* mutant *P. aeruginosa.* The bacteria (b) appear less rugose than their wild-type counterparts. There is much less microvillar pathology (mv) and extracellular material (em). The terminal web is unaffected (tw). We also find evidence of exocytosis (vesicles labelled with asterisks).
(8.19 MB TIF)

Figure S2 Wild type, but not *gacA* mutant, *P. aeruginosa* causes increased early autophagosomes.
*fer-15;fem-1* sterile animals were infected with wild type or *gacA* mutant *P. aeruginosa* PA14 for 24 h. Autophagosomes (Fig. 1H–I) were counted in TEM transversal sections of both intestinal epithelial cells, and are represented as means of n = 6 different sections each. Error bars are SEM. ***p<0.0001(Two-tailed t test).
(1.56 MB TIF)

Figure S3 Early intestinal accumulation of *S. aureus*.
**A, B, C**. High magnification micrographs of a representative animal infected with GFP-expressing *S. aureus* 4 h after initiation of infection. Accumulation in pharyngeal-intestinal valve and foregut (A), midgut (B), and rectum (C). Arrowheads indicate areas magnified in insets to illustrate the faint green haze likely due to bacterial cell lysis (A, C) and bacterial attachment to the apical surfaces of enterocytes (B). Green, GFP-*S. aureus*. Red, autofluorescent granules. Distention of the anterior intestinal lumen immediately adjacent to the pharyngeal-intestinal valve was apparent (A). There was less accumulation of bacteria in the mid section of the intestinal lumen. The bacteria appear to attach to the apical surface of the intestinal cells, as well as each other to a thickness of 3–4 bacterial cell diameters, either due to the dumbbell-shaped intestinal lumen or to direct bacteria-enterocyte and bacteria-bacteria interactions (B).
(4.07 MB TIF)

Figure S4 *S. aureus* accumulates in the intestine and causes anal swelling in *C. elegans*.
**A, B**. Timecourse of *S. aureus* intestinal accumulation. **A**, representative epifluorescence (left) and Nomarski (right) micrographs of animals infected with GFP-expressing *S. aureus*, illustrating three categories of intestinal accumulation observed. a, p, indicate anterior and posterior ends respectively. **B**, quantification of intestinal accumulation of *S. aureus* (SA) and *P. aeruginosa* (PA) at different times. At early times, green haze from lysed bacteria could be misinterpreted as *P. aeruginosa* accumulation if evaluated at low magnification. N≥18 animals for each condition. **C, D, E**. Deformed anal region (Dar) phenotype during *S. aureus* infection. a, p, indicate anterior and posterior ends respectively. **C**. Nomarski micrograph showing a representative animal infected with *S. aureus* for 12 h. Note smaller size than uninfected animal shown in D. Inset, higher magnification of anal region, highlighting swelling (arrow). **D**. Micrograph of an uninfected animal. Note smooth tapering of the tail region. Inset, detail of anal region, noting the absence of swelling (arrow). Micrographs in C, D are at same magnification. **E**. Quantification of the Dar phenotype in *S. aureus*-infected animals, compared with animals feeding on heat-killed *S. aureus*. Error bars represent standard deviation. N = 82 (live bacteria), N = 97 (heat killed bacteria). **F, G, H, I, J, K**. Nomarski micrographs illustrating the Dar phenotype after 12 h of *S. aureus* infection in wild type (**F**), *egl-5* (**H**), *pmk-1* (**I**), *and unc-32* (**J**) mutant animals in contrast to non-Dar *bar-1* (**G**) and *mpk-1,unc-32* (**K**) mutants. **L**. Quantification of Dar phenotype in wild type (N = 93), *bar-1* (N = 69), *egl-5* (N = 89), *pmk-1* (N = 87), *unc-32* (107), and *mpk-1,unc-32* (N = 83) animals. ***, *p*<0.001 (two-tailed one-sample t test).
(8.78 MB TIF)

Figure S5 Complete destruction of internal structure in *S. aureus*-killed animal.
TEM micrograph of a transversal midbody section of a *S. aureus*-killed animal, after 36 h infection. The only remaining internal structure is an unidentified circular remnant (lower right). Scale bar, 2 µm.
(8.72 MB TIF)

Figure S6 α-hemolysin-independent membrane blebbing and cell lysis.
TEM micrographs of transversal sections of *S. aureus*-infected animals after 12 h infection. Red false-coloring indicates membrane blebbing and microvillus shortening, by highlighting the apical surface of the intestinal epithelial cells from the underlying terminal web to the end of the microvilli and the membrane blebs in the intestinal lumen. **A**. Section of intestinal ring 1, showing four intestinal epithelial cells (iec). Two apical junctions are visible (aj). **B**. Midbody section, showing two intestinal epithelial cells. One apical junction is visible (lower center). Asterisks indicate extensive host cell membrane blebbing. Scale bars, 0.5 µm. **C**. Cross-section of an animal infected with wild type *S. aureus* for 24 h. The box indicates section magnified in **D**. Scale bar, 10 µm. **D**. Detail of animal infected with wild type *S. aureus*. Scale bar, 2 µm. **E**. Cross-section of animal infected with α-hemolysin-defective Δ*hla* mutant *S. aureus*. The box indicates section magnified in **F**. Scale bar, 10 µm. **F**. Detail of animal infected with Δ*hla* mutant *S. aureus*. Note the upper intestinal cell is not yet lysed, whereas the lower cell is clearly lysed (indicated) and invaded by live bacteria (indicated). Also, microvillar shortening, membrane blebbing, and intestinal cell volume loss were indistinguishable from wild type at this timepoint. Scale bar, 2 µm. iec, intestinal epithelial cell; tw, terminal web; mv, microvilli.
(9.89 MB TIF)

Figure S7 *S. aureus* hemolysins are dispensable for *C. elegans* killing.
*spe-9;fer-15* sterile animals were infected with triple hemolysin Δ*hla* Δ*hlb* Δ*hlg* mutant RN6390 *S. aureus*, or with the double mutant combinations. All killed *C. elegans* with similar kinetics.
(7.35 MB TIF)

Figure S8 Extraintestinal sites of host response gene expression.
*ilys-3::GFP* expression in pharynx and unidentified cell near terminal bulb (arrow, **A**) and vulval cells (**B**). One transgenic line had *clec-70::GFP* expression in unidentified head cells (arrows, **C**), vulval and uterine muscles (**D**), rectal gland cells (arrow, **E**), and anal depressor muscle (**F**). *F53A9.8::GFP* expressed in rectal gland cells in animals feeding on *E. coli* (arrows, **G**) and on *S. aureus* for 24 h (arrows, **H**).
(8.58 MB TIF)

Figure S9 *mpk-1*/ERK is dispensable for the intestinal host response.
Transcript levels were measured in synchronized young adult animals feeding on heat-killed *E. coli* or infected with *S. aureus* for 8 h. Data are the means of two biological replicates, each replicate measured in duplicate and normalized to a control gene, expressed as the ratio of the corresponding *S. aureus-*induced levels and the basal *E. coli* levels. Error bars are SEM.
(3.22 MB TIF)

Figure S10 *tol-1*/TLR is dispensable for host survival of *S. aureus* infection.
*tol-1(nr2033)* mutants exhibit the same susceptibility to *S. aureus-*mediated killing as wild type. Animals were sterilized with *cdc-25* RNAi previous to killing assays (see Experimental Procedures). *Wild type* (LT_50_ = 75.6 h; N = 101), *tol-1* (LT_50_ = 75.35 h; N = 117; *p* = 0.8814).
(5.78 MB TIF)

Figure S11 Pathogen-specific induction of infection reporters.
**A, B, C, D**. Animals carrying *clec-60::gfp* arrays were infected with pathogens for 24 h, in parallel with non-pathogenic *E. coli* control (**A**). Induction of *clec-60::gfp* by infection with *M. nematophilum* (**C**) and *S. aureus* (**D**), and repression by infection with *P. aeruginosa* (**B**). Note vulval expression in **B** and **C** (arrows)**. E, F, G, H**. Animals carrying *F53A9.8::gfp* were infected with pathogens for 24 h, in parallel with non-pathogenic *E. coli* control (**E**). Induction of *F53A9.8::gfp* by infection with *M. nematophilum* (**G**), *S. aureus* (**H**), and *P. aeruginosa* (**F**); the levels of induction on *S. aureus* were highest. On non-pathogenic *E. coli, clec-60::GFP* was expressed at low levels, mostly in the 9^th^ ring of intestinal epithelial cells (Fig. S7A), and *F53A9.8::GFP* was weakly expressed mostly in the posterior intestine and the rectal gland cells (Fig. S7E). During infection with *M. nematophilum, clec-60::GFP* was expressed weakly in the intestine, as well as occasional expression in the vulva, consistent with previous reports (Fig. S7C, [90]). *M. nematophilum* induced moderate levels of *F53A9.8::GFP* expression in the intestine (Fig. S7G). During infection with *P. aeruginosa*, we observed reduced expression of *clec-60::GFP* in the intestine, below the level observed on *E. coli*, except for occasional expression in the vulva (Fig. S7B), and induced expression of *F53A9.8::GFP* in the intestine (Fig. S7F). Finally, during infection with *S. aureus* we observed highest expression of both reporters, mostly in the posterior half of the intestine (Fig. S7D, H). *clec-52::GFP* was also downregulated on *P. aeruginosa* (not shown).
(6.29 MB TIF)

Figure S12 *clec-60,61*/CTL overexpression is protective during *S. aureus* infection.
Transgenic animals carrying *clec-60,61* cluster extrachromosomal arrays survived longer (LT_50_ = 54.2 h; N = 79; *p* = 0.019) during *S. aureus* infection than control animals bearing arrays composed of coinjection marker and *clec-60::GFP* promoter fusion (LT_50_ = 47.3 h; N = 95).
(6.37 MB TIF)

Table S1 List of genes whose expression changed more than two-fold, after 8 h of infection with *S. aureus* RN6390.
Threshold *p*≤0.01 for significance. Public names in bold indicate genes selected for qRT-PCR analysis.
(0.09 MB XLS)

Table S2 Nextbio biogroups, defined by GO annotations, that are over-represented among expression changes 8 h after infection with *S. aureus* (SA, a) or *P. aeruginosa* (PA, b).
Key in **a**: green genes have same direction on SA and PA; red genes are SA down, PA up; bold black genes are SA up and PA down (or no change on PA).
(0.07 MB XLS)

Table S3 List of genes whose expression changed during infection with *S. aureus* and during exposure to *B. thuringiensis* PFT Cry5B.
(0.03 MB XLS)

Table S4 List of shared gene classes upregulated during infection of *C. elegans* and human neutrophils with *S. aureus*.
(0.10 MB DOC)

Table S5 List of genes whose expression changed during infection with *S. aureus*, and during infection with (**a**) *Enterococcus faecalis*, (**b**) *L. chromiireducens*, and (**c**) common late response genes.
(0.02 MB XLS)

Table S6 **A**. List of *C. elegans* strains used in this study. **B**. List of bacterial strains used in this study.
(0.06 MB DOC)

Table S7 Over-represented GO annotations among gene expression changes during *S. aureus* or *P. aeruginosa* infection for 8 h.
“Output is in the form of “Rank, N, M, X, LOD, P, P-adj, GO Attribute”, where Rank: position in the attribute list ranked by significance of association with query; N: number of genes in the most surprising subquery with this attribute; M: size of most surprising sub query; X: number of genes overall with this attribute; LOD: the logarithm (base 10) of the odds ratio; positive values indicate over-representation; P: single hypothesis one-sided P-value of the association between attribute and query (based on Fisher's Exact Test); P-adj: adjusted P-value: fraction (as a %) of 1000 null-hypothesis simulations having attributes with this single-hypothesis P value or smaller.” From FuncAssociate (http://llama.med.harvard.edu/cgi/func/funcassociate).
(0.06 MB DOC)

Text S1 Supporting text.
(0.02 MB DOC)

## References

[1] Liévin-Le Moal V, Servin AL (2006). The front line of enteric host defense against unwelcome intrusion of harmful microorganisms: mucins, antimicrobial peptides, and microbiota.. Clin Microbiol Rev.

[2] Vaishnava S, Behrendt CL, Ismail AS, Eckmann L, Hooper LV (2008). Paneth cells directly sense gut commensals and maintain homeostasis at the intestinal host-microbial interface.. Proc Natl Acad Sci U S A.

[3] Akira S, Uematsu S, Takeuchi O (2006). Pathogen recognition and innate immunity.. Cell.

[4] Hoffmann JA, Kafatos FC, Janeway CA, Ezekowitz RA (1999). Phylogenetic perspectives in innate immunity.. Science.

[5] Lemaitre B, Hoffmann JA (2007). The host defense of Drosophila melanogaster.. Annu Rev Immunol.

[6] Kurz CL, Ewbank JJ (2003). Caenorhabditis elegans: an emerging genetic model for the study of innate immunity.. Nat Rev Genet.

[7] Irazoqui JE, Ng A, Xavier RJ, Ausubel FM (2008). Role for beta-catenin and HOX transcription factors in Caenorhabditis elegans and mammalian host epithelial-pathogen interactions.. Proc Natl Acad Sci U S A.

[8] Zugasti O, Ewbank JJ (2009). Neuroimmune regulation of antimicrobial peptide expression by a noncanonical TGF-beta signaling pathway in Caenorhabditis elegans epidermis.. Nat Immunol.

[9] Irazoqui JE, Urbach JM, Ausubel FM (2010). Evolution of host innate defence: insights from Caenorhabditis elegans and primitive invertebrates.. Nat Rev Immunol.

[10] McGhee JD (2007). The C. elegans intestine.. WormBook.

[11] Sifri CD, Begun J, Ausubel FM (2005). The worm has turned—microbial virulence modeled in Caenorhabditis elegans.. Trends Microbiol.

[12] Waterfield NR, Wren BW, Ffrench-Constant RH (2004). Invertebrates as a source of emerging human pathogens.. Nat Rev Micro.

[13] Lyczak JB, Cannon CL, Pier GB (2000). Establishment of Pseudomonas aeruginosa infection: lessons from a versatile opportunist.. Microbes Infect.

[14] Lyczak JB, Cannon CL, Pier GB (2002). Lung infections associated with cystic fibrosis.. Clin Microbiol Rev.

[15] Sifri CD, Ausubel FM, Cossart P, Boquet P, Normark S, Rappuoli R (2004). Use of simple non-vertebrate hosts to model mammalian pathogenesis.. Cellular Microbiology. 2 ed.

[16] Cuny C, Friedrich A, Kozytska S, Layer F, Nübel U (2009). Emergence of methicillin-resistant Staphylococcus aureus (MRSA) in different animal species.. Int J Med Microbiol.

[17] Sifri CD, Begun J, Ausubel FM, Calderwood SB (2003). Caenorhabditis elegans as a model host for Staphylococcus aureus pathogenesis.. Infect Immun.

[18] Boucher HW, Corey GR (2008). Epidemiology of methicillin-resistant Staphylococcus aureus.. CLIN INFECT DIS.

[19] Graham PL, Lin SX, Larson EL (2006). A U.S. population-based survey of Staphylococcus aureus colonization.. Annals of Internal Medicine.

[20] Gordon RJ, Lowy FD (2008). Pathogenesis of methicillin-resistant Staphylococcus aureus infection.. CLIN INFECT DIS.

[21] Diep BA, Otto M (2008). The role of virulence determinants in community-associated MRSA pathogenesis.. Trends Microbiol.

[22] Nizet V (2007). Understanding how leading bacterial pathogens subvert innate immunity to reveal novel therapeutic targets.. J Allergy Clin Immunol.

[23] von Köckritz-Blickwede M, Rohde M, Oehmcke S, Miller LS, Cheung AL (2008). Immunological mechanisms underlying the genetic predisposition to severe Staphylococcus aureus infection in the mouse model.. Am J Pathol.

[24] Tan MW, Mahajan-Miklos S, Ausubel FM (1999). Killing of Caenorhabditis elegans by Pseudomonas aeruginosa used to model mammalian bacterial pathogenesis.. Proc Natl Acad Sci USA.

[25] Tan MW, Rahme LG, Sternberg JA, Tompkins RG, Ausubel FM (1999). Pseudomonas aeruginosa killing of Caenorhabditis elegans used to identify P. aeruginosa virulence factors.. Proc Natl Acad Sci USA.

[26] Garsin DA, Sifri CD, Mylonakis E, Qin X, Singh KV (2001). A simple model host for identifying Gram-positive virulence factors.. Proc Natl Acad Sci USA.

[27] Powell JR, Kim DH, Ausubel FM (2009). The G protein-coupled receptor FSHR-1 is required for the Caenorhabditis elegans innate immune response.. Proc Natl Acad Sci U S A.

[28] Garsin DA, Villanueva JM, Begun J, Kim DH, Sifri CD (2003). Long-lived C. elegans daf-2 mutants are resistant to bacterial pathogens.. Science.

[29] Kim DH, Feinbaum R, Alloing G, Emerson FE, Garsin DA (2002). A conserved p38 MAP kinase pathway in Caenorhabditis elegans innate immunity.. Science.

[30] Begun J, Sifri CD, Goldman S, Calderwood SB, Ausubel FM (2005). Staphylococcus aureus virulence factors identified by using a high-throughput Caenorhabditis elegans-killing model.. Infect Immun.

[31] Bae T, Banger AK, Wallace A, Glass EM, Aslund F (2004). Staphylococcus aureus virulence genes identified by bursa aurealis mutagenesis and nematode killing.. Proc Natl Acad Sci USA.

[32] Skaar EP, Humayun M, Bae T, DeBord KL, Schneewind O (2004). Iron-source preference of Staphylococcus aureus infections.. Science.

[33] Mahajan-Miklos S, Tan MW, Rahme LG, Ausubel FM (1999). Molecular mechanisms of bacterial virulence elucidated using a Pseudomonas aeruginosa-Caenorhabditis elegans pathogenesis model.. Cell.

[34] Darby C, Cosma CL, Thomas JH, Manoil C (1999). Lethal paralysis of Caenorhabditis elegans by Pseudomonas aeruginosa.. Proc Natl Acad Sci USA.

[35] Kuehn MJ, Kesty NC (2005). Bacterial outer membrane vesicles and the host-pathogen interaction.. Genes & Development.

[36] Lam J, Chan R, Lam K, Costerton JW (1980). Production of mucoid microcolonies by Pseudomonas aeruginosa within infected lungs in cystic fibrosis.. Infect Immun.

[37] Naik S, Smith F, Ho J, Croft NM, Domizio P (2008). Staphylococcal enterotoxins G and I, a cause of severe but reversible neonatal enteropathy.. Clin Gastroenterol Hepatol.

[38] Kotler DP, Sandkovsky U, Schlievert PM, Sordillo EM (2007). Toxic shock-like syndrome associated with staphylococcal enterocolitis in an HIV-infected man.. CLIN INFECT DIS.

[39] Amaral MM, Coelho LR, Flores RP, Souza RR, Silva-Carvalho MC (2005). The predominant variant of the Brazilian epidemic clonal complex of methicillin-resistant Staphylococcus aureus has an enhanced ability to produce biofilm and to adhere to and invade airway epithelial cells.. J Infect Dis.

[40] Kamaras J, Murrell WG (2001). Intestinal epithelial damage in sids babies and its similarity to that caused by bacterial toxins in the rabbit.. Pathology.

[41] Kamaras J, Murrell WG (2001). The effect of bacterial enterotoxins implicated in SIDS on the rabbit intestine.. Pathology.

[42] da Silva MCA, Zahm J-M, Gras D, Bajolet O, Abely M (2004). Dynamic interaction between airway epithelial cells and Staphylococcus aureus.. Am J Physiol Lung Cell Mol Physiol.

[43] Zetola N, Francis JS, Nuermberger EL, Bishai WR (2005). Community-acquired meticillin-resistant Staphylococcus aureus: an emerging threat.. Lancet Infect Dis.

[44] Troemel ER, Chu SW, Reinke V, Lee SS, Ausubel FM (2006). p38 MAPK regulates expression of immune response genes and contributes to longevity in C. elegans.. PLoS Genet.

[45] Mashburn-Warren L, McLean RJC, Whiteley M (2008). Gram-negative outer membrane vesicles: beyond the cell surface.. Geobiology.

[46] Hodgkin J, Kuwabara PE, Corneliussen B (2000). A novel bacterial pathogen, Microbacterium nematophilum, induces morphological change in the nematode C. elegans.. Curr Biol.

[47] Nicholas HR, Hodgkin J (2004). The ERK MAP kinase cascade mediates tail swelling and a protective response to rectal infection in C. elegans.. Curr Biol.

[48] Nicholas HR, Hodgkin J (2009). The C. elegans Hox gene egl-5 is required for correct development of the hermaphrodite hindgut and for the response to rectal infection by Microbacterium nematophilum.. Dev Biol.

[49] Shimoda M, Ohki K, Shimamoto Y, Kohashi O (1995). Morphology of defensin-treated Staphylococcus aureus.. Infect Immun.

[50] Wong D, Bazopoulou D, Pujol N, Tavernarakis N, Ewbank JJ (2007). Genome-wide investigation reveals pathogen-specific and shared signatures in the response of Caenorhabditis elegans to infection.. Genome Biol.

[51] Geijtenbeek TBH, Gringhuis SI (2009). Signalling through C-type lectin receptors: shaping immune responses.. Nat Rev Immunol.

[52] Yu Y, Yu Y, Huang H, Feng K, Pan M (2007). A short-form C-type lectin from amphioxus acts as a direct microbial killing protein via interaction with peptidoglycan and glucan.. J Immunol.

[53] Cash HL, Whitham CV, Behrendt CL, Hooper LV (2006). Symbiotic bacteria direct expression of an intestinal bactericidal lectin.. Science.

[54] Kabelitz D, Medzhitov R (2007). Innate immunity—cross-talk with adaptive immunity through pattern recognition receptors and cytokines.. Curr Opin Immunol.

[55] Huffman DL, Abrami L, Sasik R, Corbeil J, van der Goot FG (2004). Mitogen-activated protein kinase pathways defend against bacterial pore-forming toxins.. Proc Natl Acad Sci USA.

[56] Van Gilst MR, Hadjivassiliou H, Yamamoto KR (2005). A Caenorhabditis elegans nutrient response system partially dependent on nuclear receptor NHR-49.. Proc Natl Acad Sci USA.

[57] Vance RE, Isberg RR, Portnoy DA (2009). Patterns of pathogenesis: discrimination of pathogenic and nonpathogenic microbes by the innate immune system.. Cell Host & Microbe.

[58] Pujol N, Link EM, Liu LX, Kurz CL, Alloing G (2001). A reverse genetic analysis of components of the Toll signaling pathway in Caenorhabditis elegans.. Curr Biol.

[59] Tenor JL, Aballay A (2008). A conserved Toll-like receptor is required for Caenorhabditis elegans innate immunity.. EMBO Rep.

[60] Takeda K, Kaisho T, Akira S (2003). Toll-like receptors.. Annu Rev Immunol.

[61] O'Rourke D, Baban D, Demidova M, Mott R, Hodgkin J (2006). Genomic clusters, putative pathogen recognition molecules, and antimicrobial genes are induced by infection of C. elegans with M. nematophilum.. Genome Res.

[62] Schulenburg H, Hoeppner MP, Weiner J, Bornberg-Bauer E (2008). Specificity of the innate immune system and diversity of C-type lectin domain (CTLD) proteins in the nematode Caenorhabditis elegans.. Immunobiology.

[63] Sigmond T, Fehér J, Baksa A, Pásti G, Pálfia Z (2008). Qualitative and quantitative characterization of autophagy in Caenorhabditis elegans by electron microscopy.. Meth Enzymol.

[64] Jia K, Thomas C, Akbar M, Sun Q, Adams-Huet B (2009). Autophagy genes protect against Salmonella typhimurium infection and mediate insulin signaling-regulated pathogen resistance.. Proc Natl Acad Sci USA.

[65] Deretic V (2009). Multiple regulatory and effector roles of autophagy in immunity.. Curr Opin Immunol.

[66] Wareham DW, Papakonstantinopoulou A, Curtis MA (2005). The Pseudomonas aeruginosa PA14 type III secretion system is expressed but not essential to virulence in the Caenorhabditis elegans-P. aeruginosa pathogenicity model.. FEMS Microbiol Lett.

[67] McBroom AJ, Kuehn MJ (2007). Release of outer membrane vesicles by Gram-negative bacteria is a novel envelope stress response.. Mol Microbiol.

[68] Troemel E, Chu S, Reinke V, Lee S, Ausubel FM (2006). p38 MAPK regulates expression of immune response genes and contributes to longevity in C. elegans.. PLoS Genet.

[69] Estes KA, Dunbar TL, Powell JR, Ausubel FM, Troemel ER (2010). bZIP transcription factor zip-2 mediates an early response to Pseudomonas aeruginosa infection in Caenorhabditis elegans.. Proc Natl Acad Sci USA.

[70] Medzhitov R (2009). Approaching the asymptote: 20 years later.. Immunity.

[71] Vance R, Isberg R, Portnoy D (2009). Patterns of pathogenesis: discrimination of pathogenic and nonpathogenic microbes by the innate immune system.. Cell Host & Microbe.

[72] Fernandes-Alnemri T, Wu J, Yu J-W, Datta P, Miller B (2007). The pyroptosome: a supramolecular assembly of ASC dimers mediating inflammatory cell death via caspase-1 activation.. Cell Death and Differentiation.

[73] Vollmer W, Blanot D, de Pedro MA (2008). Peptidoglycan structure and architecture.. FEMS Microbiol Rev.

[74] Borjesson DL, Kobayashi SD, Whitney AR, Voyich JM, Argue CM (2005). Insights into pathogen immune evasion mechanisms: Anaplasma phagocytophilum fails to induce an apoptosis differentiation program in human neutrophils.. J Immunol.

[75] Gems D, McElwee JJ (2005). Broad spectrum detoxification: the major longevity assurance process regulated by insulin/IGF-1 signaling?. Mech Ageing Dev.

[76] Dann SM, Eckmann L (2007). Innate immune defenses in the intestinal tract.. Curr Opin Gastroenterol.

[77] Salzet M (2001). Vertebrate innate immunity resembles a mosaic of invertebrate immune responses.. Trends Immunol.

[78] Alper S, Laws R, Lackford B, Boyd WA, Dunlap P (2008). Identification of innate immunity genes and pathways using a comparative genomics approach.. Proc Natl Acad Sci USA.

[79] Muir RE, Tan MW (2008). Virulence of Leucobacter chromiireducens subsp. solipictus to Caenorhabditis elegans: characterization of a novel host-pathogen interaction.. Appl Environ Microbiol.

[80] Pearce EL, Walsh MC, Cejas PJ, Harms GM, Shen H (2009). Enhancing CD8 T-cell memory by modulating fatty acid metabolism.. Nature.

[81] Anagnostou SH, Shepherd PR (2008). Glucose induces an autocrine activation of the Wnt/beta-catenin pathway in macrophage cell lines.. Biochem J.

[82] Bensinger SJ, Tontonoz P (2008). Integration of metabolism and inflammation by lipid-activated nuclear receptors.. Nature.

[83] Froy O (2005). Regulation of mammalian defensin expression by Toll-like receptor-dependent and independent signalling pathways.. Cell Microbiol.

[84] Evans EA, Chen WC, Tan M-W (2008). The DAF-2 insulin-like signaling pathway independently regulates aging and immunity in C. elegans.. Aging Cell.

[85] Watanabe A, Miyazawa S, Kitami M, Tabunoki H, Ueda K (2006). Characterization of a novel C-type lectin, Bombyx mori multibinding protein, from the B. mori hemolymph: mechanism of wide-range microorganism recognition and role in immunity.. J Immunol.

[86] Yu XQ, Gan H, Kanost MR (1999). Immulectin, an inducible C-type lectin from an insect, Manduca sexta, stimulates activation of plasma prophenol oxidase.. Insect Biochem Mol Biol.

[87] Yu X-Q, Kanost MR (2004). Immulectin-2, a pattern recognition receptor that stimulates hemocyte encapsulation and melanization in the tobacco hornworm, Manduca sexta.. Dev Comp Immunol.

[88] Powell JR, Ausubel FM (2008). Models of Caenorhabditis elegans infection by bacterial and fungal pathogens.. Methods Mol Biol.

[89] Weng L, Dai H, Zhan Y, He Y, Stepaniants SB (2006). Rosetta error model for gene expression analysis.. Bioinformatics.

[90] O'rourke D, Baban D, Demidova M, Mott R, Hodgkin J (2006). Genomic clusters, putative pathogen recognition molecules, and antimicrobial genes are induced by infection of C. elegans with M. nematophilum.. Genome Research.

[91] Grewal A, Lambert P, Stockton J (2007). Analysis of expression data: an overview.. Current protocols in human genetics/editorial board, Jonathan L Haines [et al].

[92] Gravato-Nobre MJ, Nicholas HR, Nijland R, O'rourke D, Whittington DE (2005). Multiple genes affect sensitivity of Caenorhabditis elegans to the bacterial pathogen Microbacterium nematophilum.. Genetics.

[93] Pfaffl MW (2001). A new mathematical model for relative quantification in real-time RT-PCR.. Nucleic Acids Res.

[94] Hobert O (2002). PCR fusion-based approach to create reporter gene constructs for expression analysis in transgenic C. elegans.. BioTechniques.

[95] Timmons L, Court DL, Fire A (2001). Ingestion of bacterially expressed dsRNAs can produce specific and potent genetic interference in Caenorhabditis elegans.. Gene.

[96] Reboul J, Vaglio P, Rual J-F, Lamesch P, Martinez M (2003). C. elegans ORFeome version 1.1: experimental verification of the genome annotation and resource for proteome-scale protein expression.. Nat Genet.

[97] Rual JF, Ceron J, Koreth J, Hao T, Nicot AS (2004). Toward improving Caenorhabditis elegans phenome mapping with an ORFeome-based RNAi library.. Genome Res.

[98] Tusher VG, Tibshirani R, Chu G (2001). Significance analysis of microarrays applied to the ionizing radiation response.. Proc Natl Acad Sci USA.

[99] Petalcorin MIR, Joshua GW, Agapow P-M, Dolphin CT (2005). The fmo genes of Caenorhabditis elegans and C. briggsae: characterisation, gene expression and comparative genomic analysis.. Gene.

[100] Pauli F, Liu Y, Kim YA, Chen P-J, Kim SK (2006). Chromosomal clustering and GATA transcriptional regulation of intestine-expressed genes in C. elegans.. Development.

