# Supplementary figures and images for: Distinct Pathogenesis and Host Responses during Infection of C. elegans by P. aeruginosa and S. aureus
Source: PLoS Pathog. 2010 Jul 1;6(7):e1000982. doi: 10.1371/journal.ppat.1000982 (PMC2895663; doi:10.1371/journal.ppat.1000982)

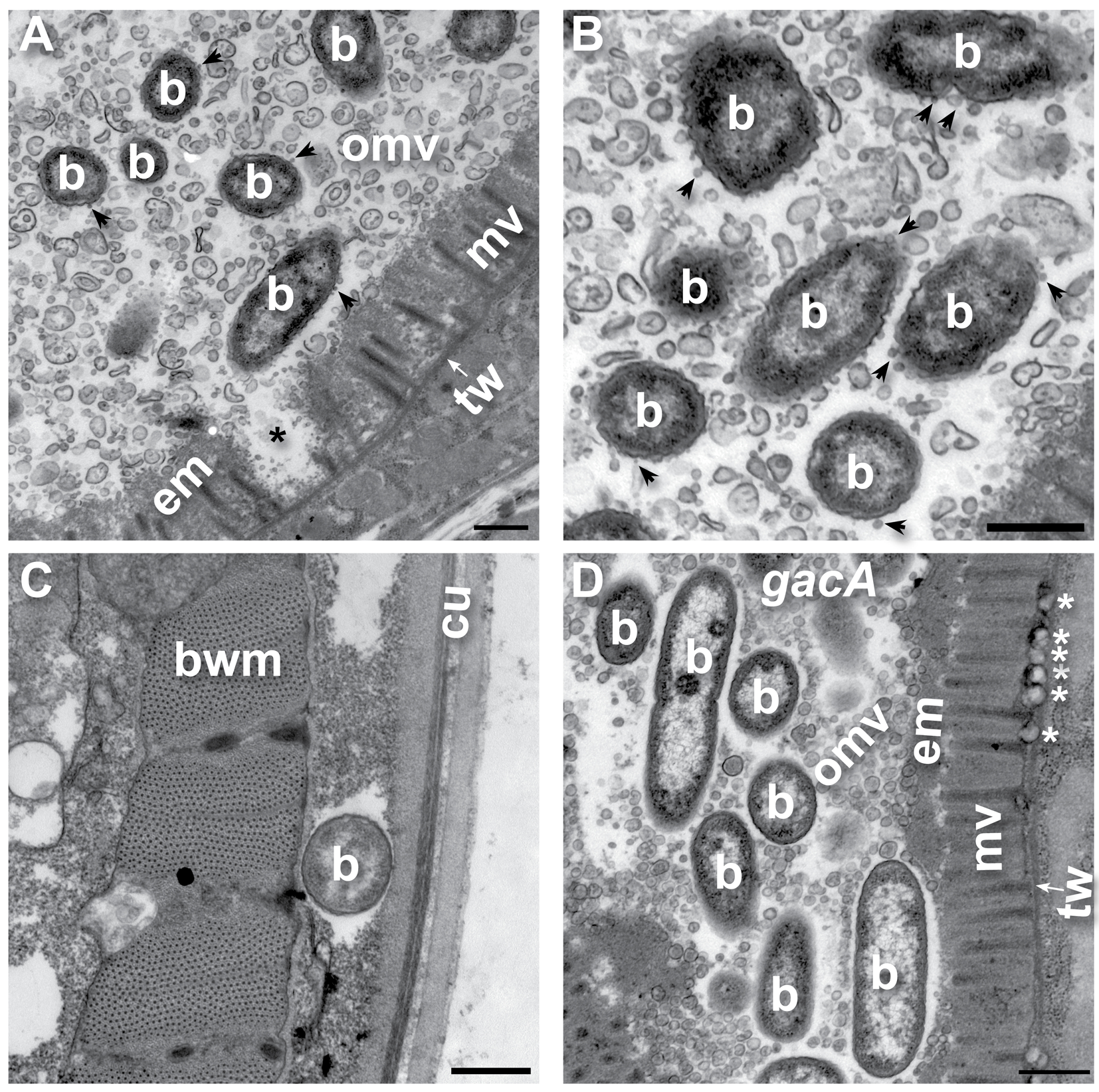

Supplement: Figure S1 — P. aeruginosa makes putative outer membrane vesicles (OMVs), disrupts the brush border, and penetrates the epithelial barrier. A–D. TEM micrographs of P. aeruginosa-infected animals after 48 h infection. Scale bars, 0.5 µm. A. Detail of intestinal lumen filled with OMVs and bacterial cells (b), and brush border (mv) coated with extracellular material (em). Note disruption of the microvilli (black asterisk) and OMV shedding from the bacterial cells (black arrowheads). At this time point the terminal web (tw) appears whole. B. High magnification TEM showing apparent OMV shedding off bacterial cells (b, indicated with black arrowheads). C. Example of distal dissemination of P. aeruginosa. A bacterial cell (b) is shown between the body-wall muscle (bwm) and the cuticle (cu), which is the exoskeleton of the animal. D. Detail of animal infected with gacA mutant P. aeruginosa. The bacteria (b) appear less rugose than their wild-type counterparts. There is much less microvillar pathology (mv) and extracellular material (em). The terminal web is unaffected (tw). We also find evidence of exocytosis (vesicles labelled with asterisks). (8.19 MB TIF) [file ppat.1000982.s001.tif]

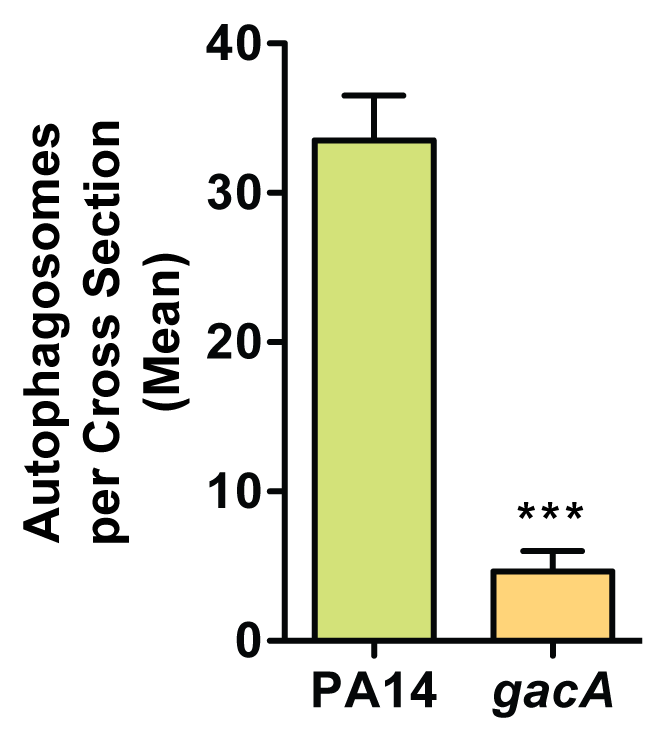

Supplement: Figure S2 — Wild type, but not gacA mutant, P. aeruginosa causes increased early autophagosomes. fer-15;fem-1 sterile animals were infected with wild type or gacA mutant P. aeruginosa PA14 for 24 h. Autophagosomes (Fig. 1H–I) were counted in TEM transversal sections of both intestinal epithelial cells, and are represented as means of n = 6 different sections each. Error bars are SEM. ***p<0.0001(Two-tailed t test). (1.56 MB TIF) [file ppat.1000982.s002.tif]

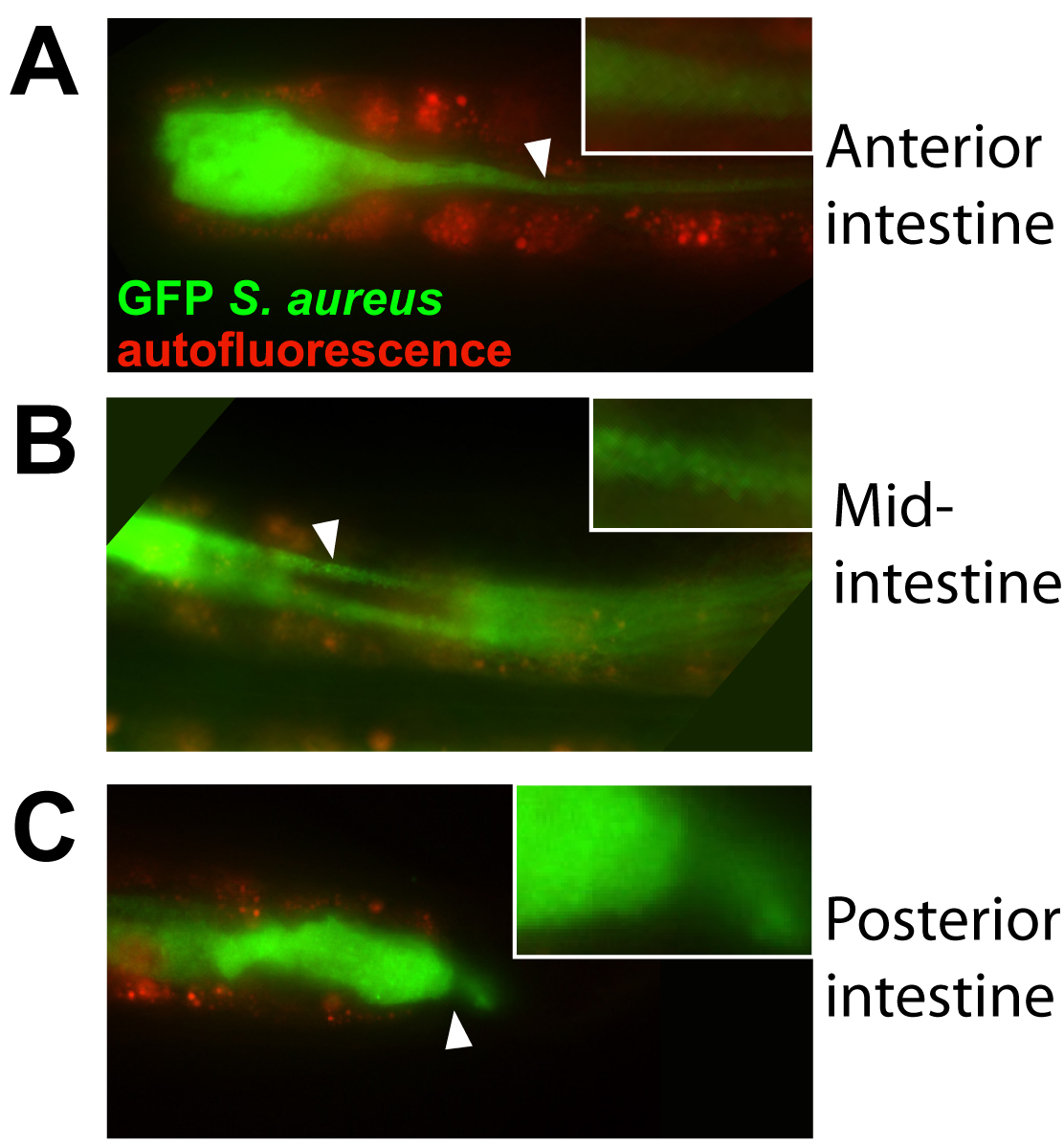

Supplement: Figure S3 — Early intestinal accumulation of S. aureus. A, B, C. High magnification micrographs of a representative animal infected with GFP-expressing S. aureus 4 h after initiation of infection. Accumulation in pharyngeal-intestinal valve and foregut (A), midgut (B), and rectum (C). Arrowheads indicate areas magnified in insets to illustrate the faint green haze likely due to bacterial cell lysis (A, C) and bacterial attachment to the apical surfaces of enterocytes (B). Green, GFP-S. aureus. Red, autofluorescent granules. Distention of the anterior intestinal lumen immediately adjacent to the pharyngeal-intestinal valve was apparent (A). There was less accumulation of bacteria in the mid section of the intestinal lumen. The bacteria appear to attach to the apical surface of the intestinal cells, as well as each other to a thickness of 3–4 bacterial cell diameters, either due to the dumbbell-shaped intestinal lumen or to direct bacteria-enterocyte and bacteria-bacteria interactions (B). (4.07 MB TIF) [file ppat.1000982.s003.tif]

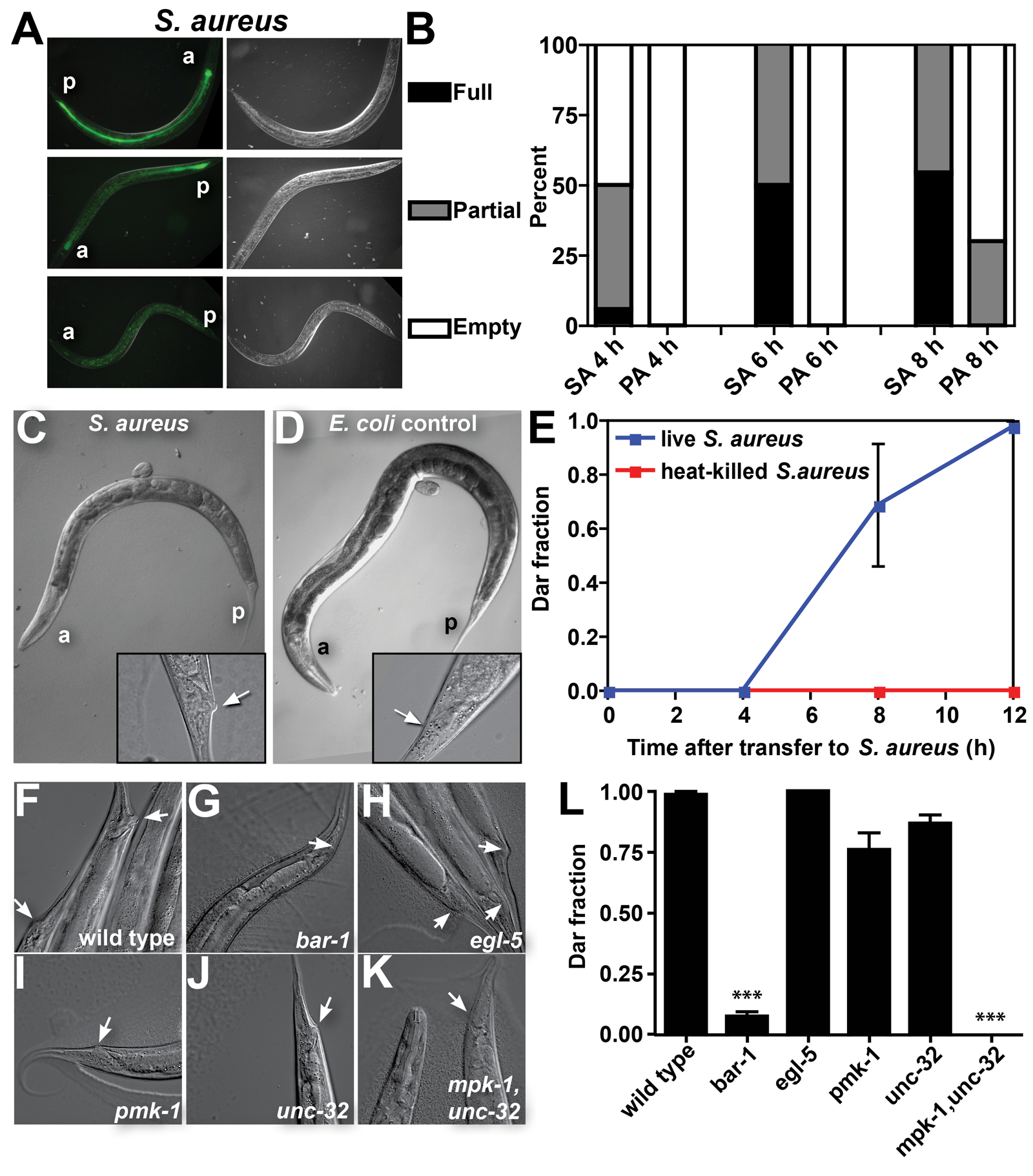

Supplement: Figure S4 — S. aureus accumulates in the intestine and causes anal swelling in C. elegans. A, B. Timecourse of S. aureus intestinal accumulation. A, representative epifluorescence (left) and Nomarski (right) micrographs of animals infected with GFP-expressing S. aureus, illustrating three categories of intestinal accumulation observed. a, p, indicate anterior and posterior ends respectively. B, quantification of intestinal accumulation of S. aureus (SA) and P. aeruginosa (PA) at different times. At early times, green haze from lysed bacteria could be misinterpreted as P. aeruginosa accumulation if evaluated at low magnification. N≥18 animals for each condition. C, D, E. Deformed anal region (Dar) phenotype during S. aureus infection. a, p, indicate anterior and posterior ends respectively. C. Nomarski micrograph showing a representative animal infected with S. aureus for 12 h. Note smaller size than uninfected animal shown in D. Inset, higher magnification of anal region, highlighting swelling (arrow). D. Micrograph of an uninfected animal. Note smooth tapering of the tail region. Inset, detail of anal region, noting the absence of swelling (arrow). Micrographs in C, D are at same magnification. E. Quantification of the Dar phenotype in S. aureus-infected animals, compared with animals feeding on heat-killed S. aureus. Error bars represent standard deviation. N = 82 (live bacteria), N = 97 (heat killed bacteria). F, G, H, I, J, K. Nomarski micrographs illustrating the Dar phenotype after 12 h of S. aureus infection in wild type (F), egl-5 (H), pmk-1 (I), and unc-32 (J) mutant animals in contrast to non-Dar bar-1 (G) and mpk-1,unc-32 (K) mutants. L. Quantification of Dar phenotype in wild type (N = 93), bar-1 (N = 69), egl-5 (N = 89), pmk-1 (N = 87), unc-32 (107), and mpk-1,unc-32 (N = 83) animals. ***, p<0.001 (two-tailed one-sample t test). (8.78 MB TIF) [file ppat.1000982.s004.tif]

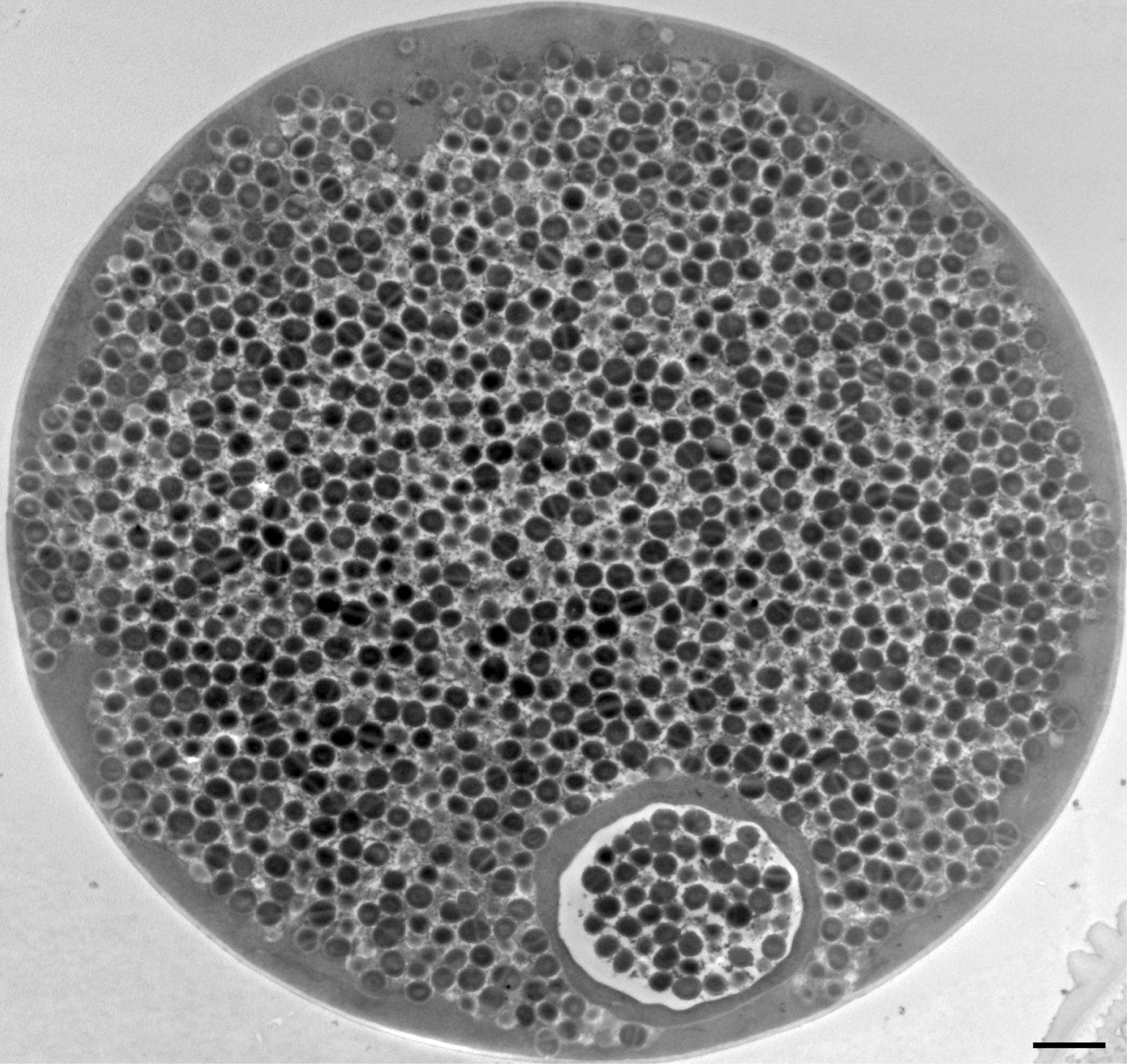

Supplement: Figure S5 — Complete destruction of internal structure in S. aureus-killed animal. TEM micrograph of a transversal midbody section of a S. aureus-killed animal, after 36 h infection. The only remaining internal structure is an unidentified circular remnant (lower right). Scale bar, 2 µm. (8.72 MB TIF) [file ppat.1000982.s005.tif]

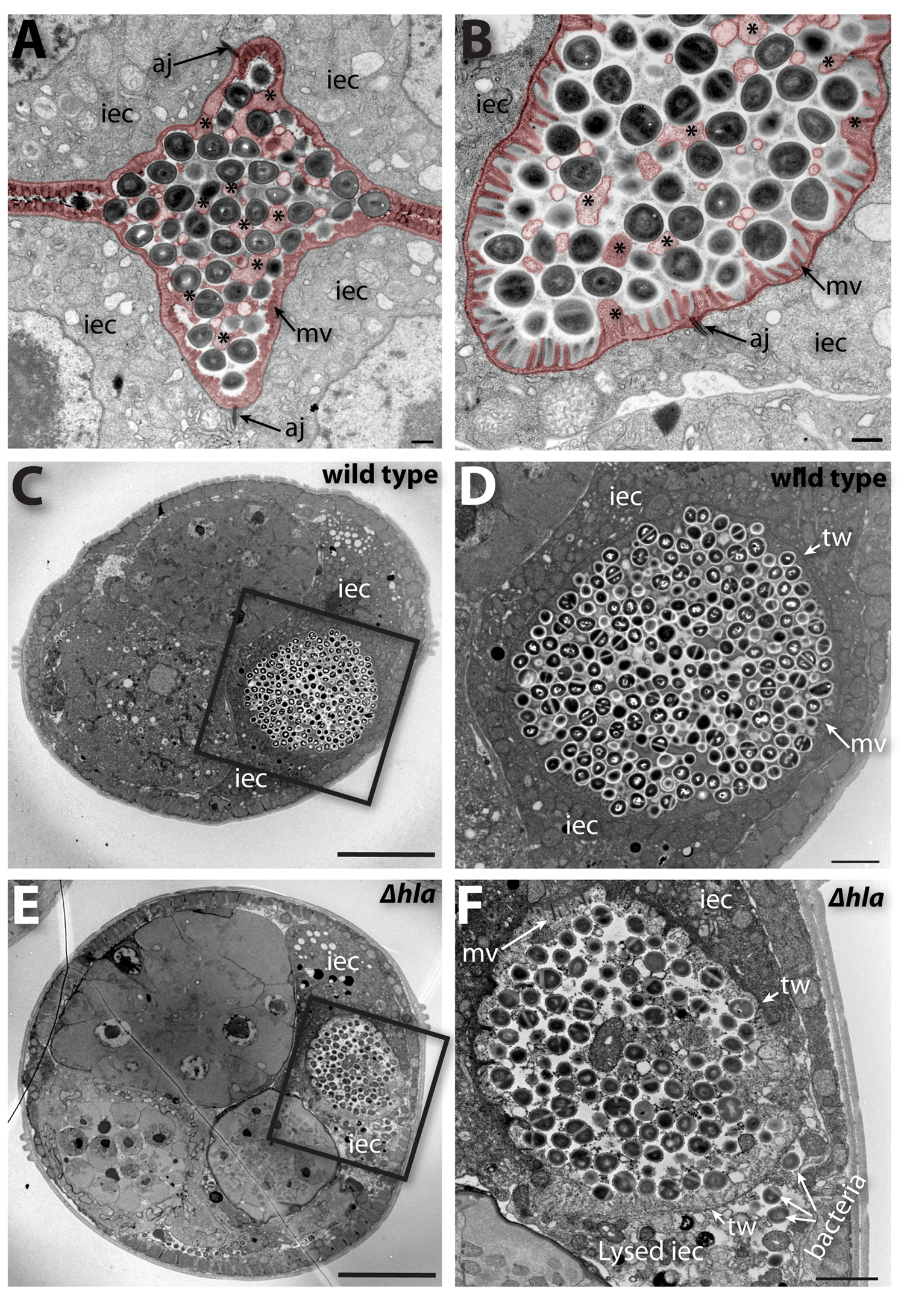

Supplement: Figure S6 — α-hemolysin-independent membrane blebbing and cell lysis. TEM micrographs of transversal sections of S. aureus-infected animals after 12 h infection. Red false-coloring indicates membrane blebbing and microvillus shortening, by highlighting the apical surface of the intestinal epithelial cells from the underlying terminal web to the end of the microvilli and the membrane blebs in the intestinal lumen. A. Section of intestinal ring 1, showing four intestinal epithelial cells (iec). Two apical junctions are visible (aj). B. Midbody section, showing two intestinal epithelial cells. One apical junction is visible (lower center). Asterisks indicate extensive host cell membrane blebbing. Scale bars, 0.5 µm. C. Cross-section of an animal infected with wild type S. aureus for 24 h. The box indicates section magnified in D. Scale bar, 10 µm. D. Detail of animal infected with wild type S. aureus. Scale bar, 2 µm. E. Cross-section of animal infected with α-hemolysin-defective Δhla mutant S. aureus. The box indicates section magnified in F. Scale bar, 10 µm. F. Detail of animal infected with Δhla mutant S. aureus. Note the upper intestinal cell is not yet lysed, whereas the lower cell is clearly lysed (indicated) and invaded by live bacteria (indicated). Also, microvillar shortening, membrane blebbing, and intestinal cell volume loss were indistinguishable from wild type at this timepoint. Scale bar, 2 µm. iec, intestinal epithelial cell; tw, terminal web; mv, microvilli. (9.89 MB TIF) [file ppat.1000982.s006.tif]

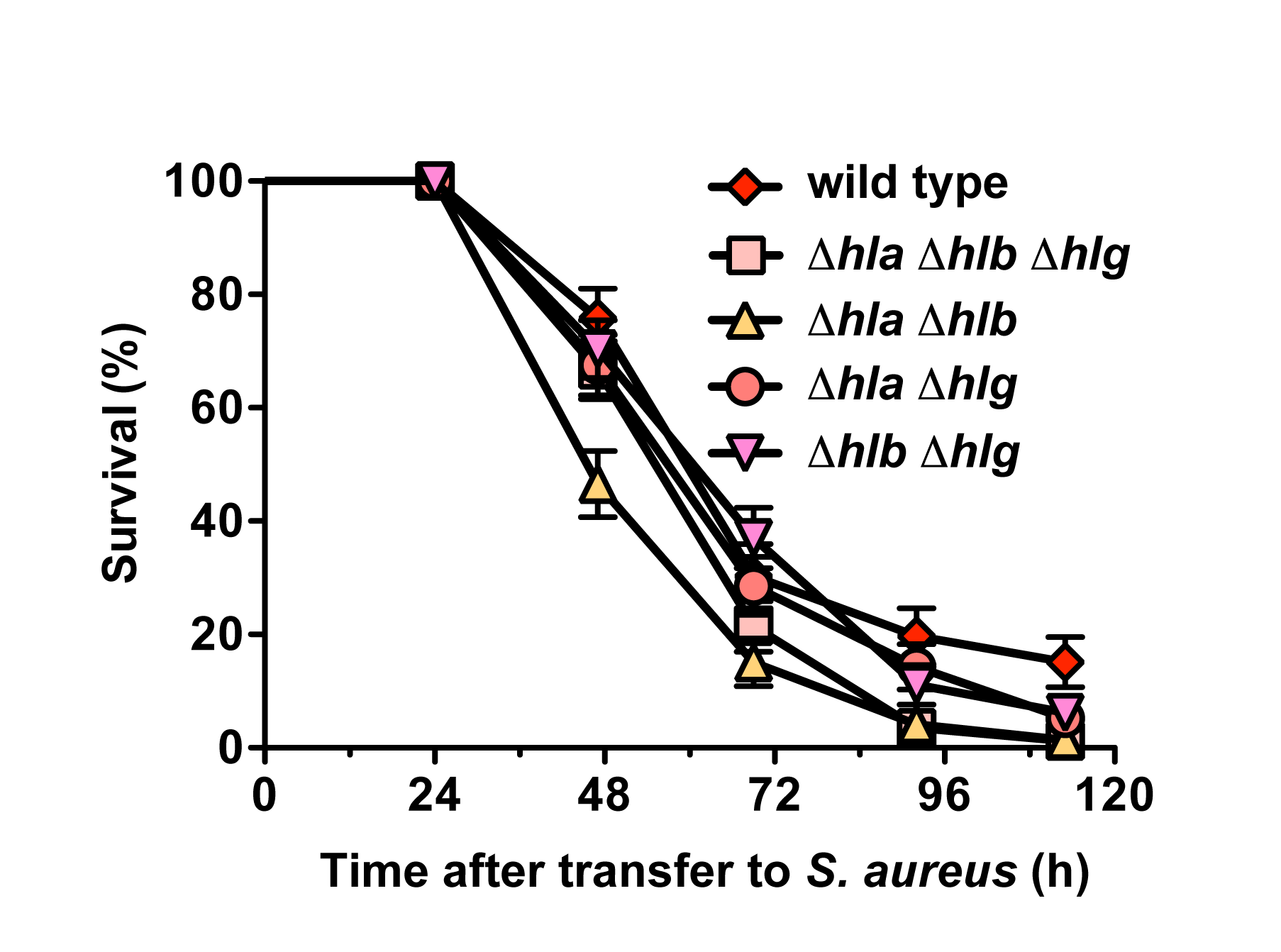

Supplement: Figure S7 — S. aureus hemolysins are dispensable for C. elegans killing. spe-9;fer-15 sterile animals were infected with triple hemolysin Δhla Δhlb Δhlg mutant RN6390 S. aureus, or with the double mutant combinations. All killed C. elegans with similar kinetics. (7.35 MB TIF) [file ppat.1000982.s007.tif]

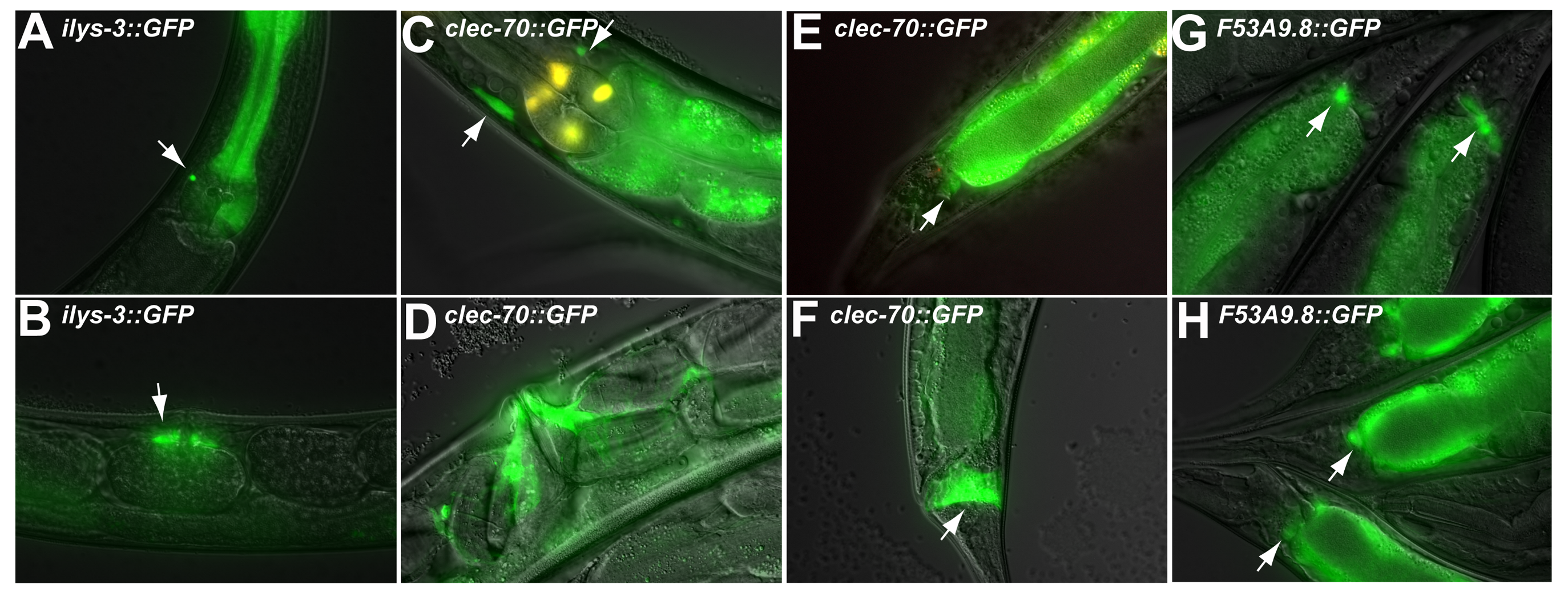

Supplement: Figure S8 — Extraintestinal sites of host response gene expression. ilys-3::GFP expression in pharynx and unidentified cell near terminal bulb (arrow, A) and vulval cells (B). One transgenic line had clec-70::GFP expression in unidentified head cells (arrows, C), vulval and uterine muscles (D), rectal gland cells (arrow, E), and anal depressor muscle (F). F53A9.8::GFP expressed in rectal gland cells in animals feeding on E. coli (arrows, G) and on S. aureus for 24 h (arrows, H). (8.58 MB TIF) [file ppat.1000982.s008.tif]

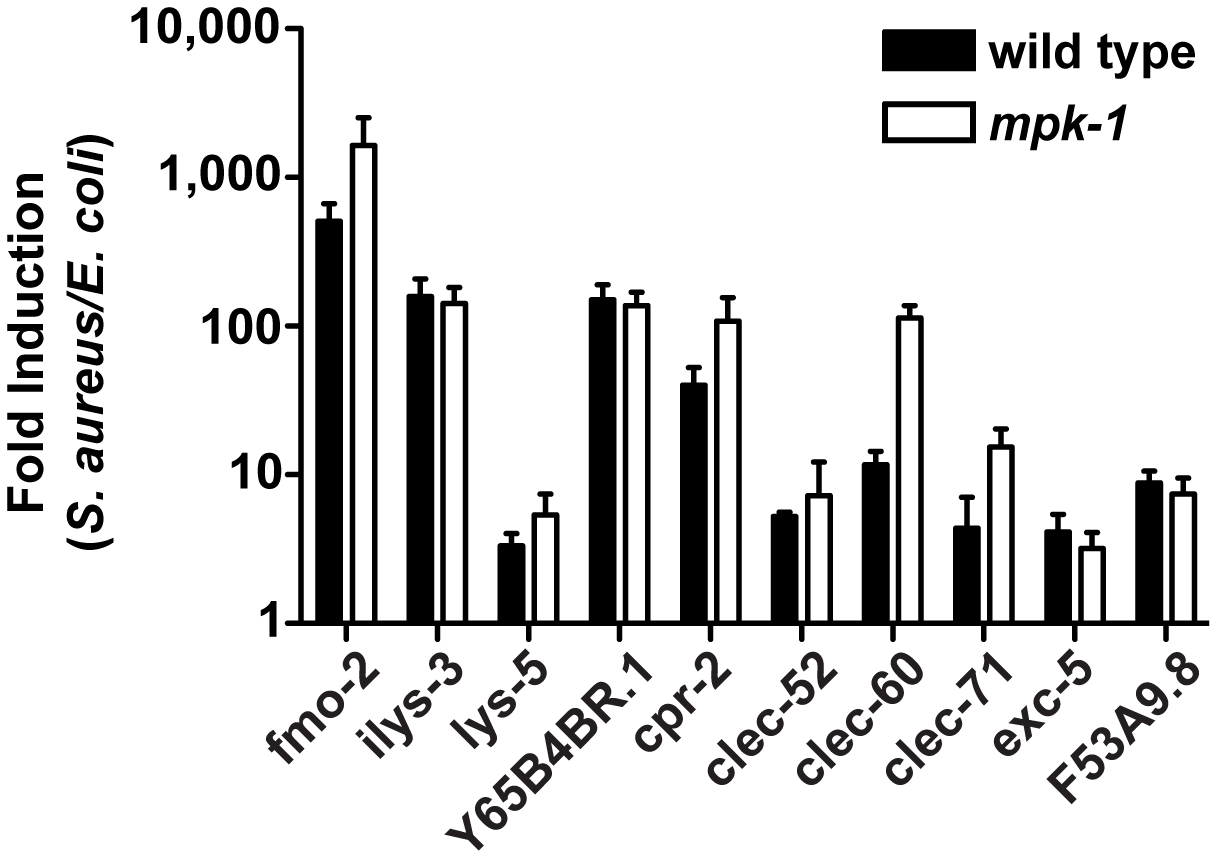

Supplement: Figure S9 — mpk-1/ERK is dispensable for the intestinal host response. Transcript levels were measured in synchronized young adult animals feeding on heat-killed E. coli or infected with S. aureus for 8 h. Data are the means of two biological replicates, each replicate measured in duplicate and normalized to a control gene, expressed as the ratio of the corresponding S. aureus-induced levels and the basal E. coli levels. Error bars are SEM. (3.22 MB TIF) [file ppat.1000982.s009.tif]

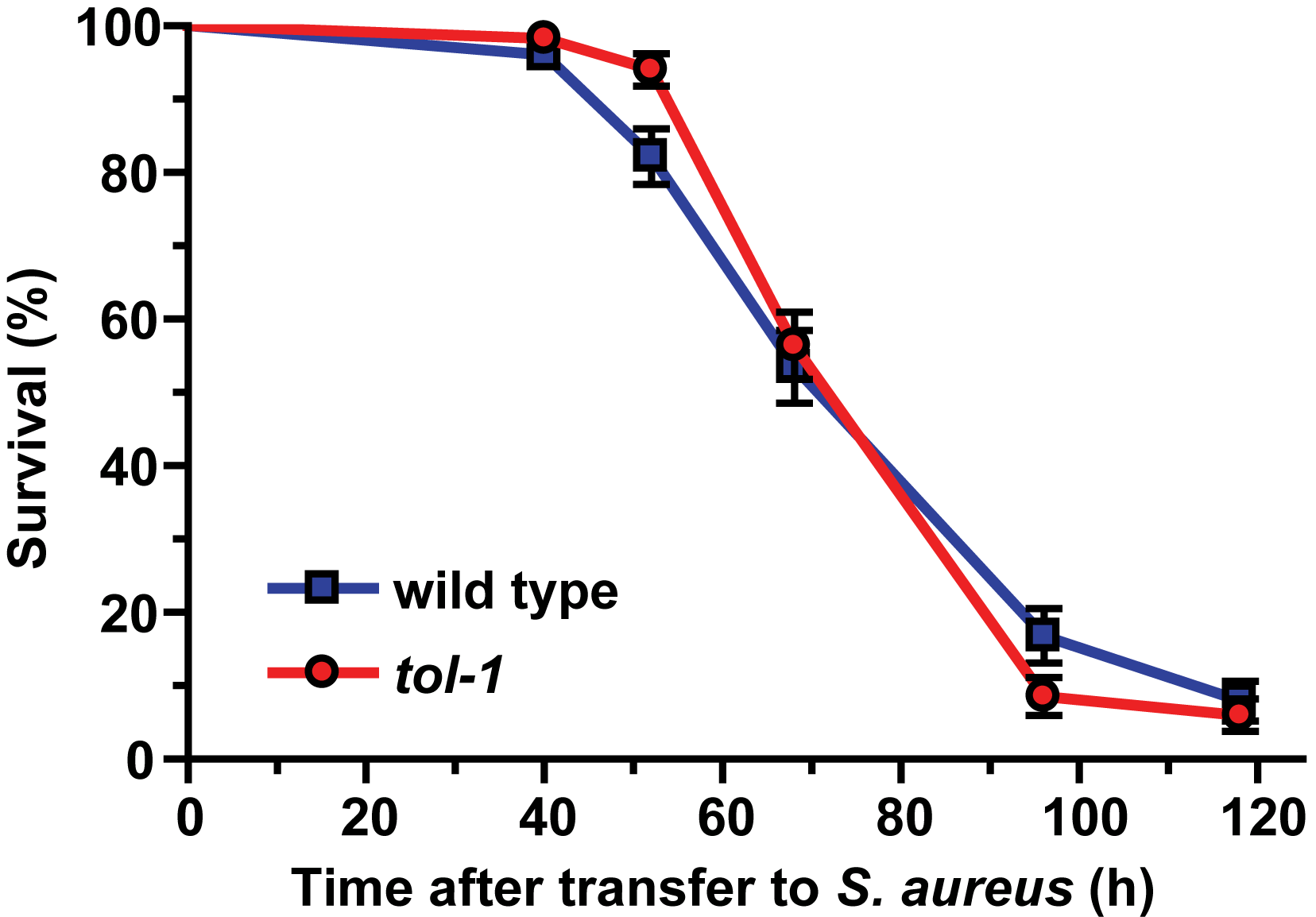

Supplement: Figure S10 — tol-1/TLR is dispensable for host survival of S. aureus infection. tol-1(nr2033) mutants exhibit the same susceptibility to S. aureus-mediated killing as wild type. Animals were sterilized with cdc-25 RNAi previous to killing assays (see Experimental Procedures). Wild type (LT50 = 75.6 h; N = 101), tol-1 (LT50 = 75.35 h; N = 117; p = 0.8814). (5.78 MB TIF) [file ppat.1000982.s010.tif]

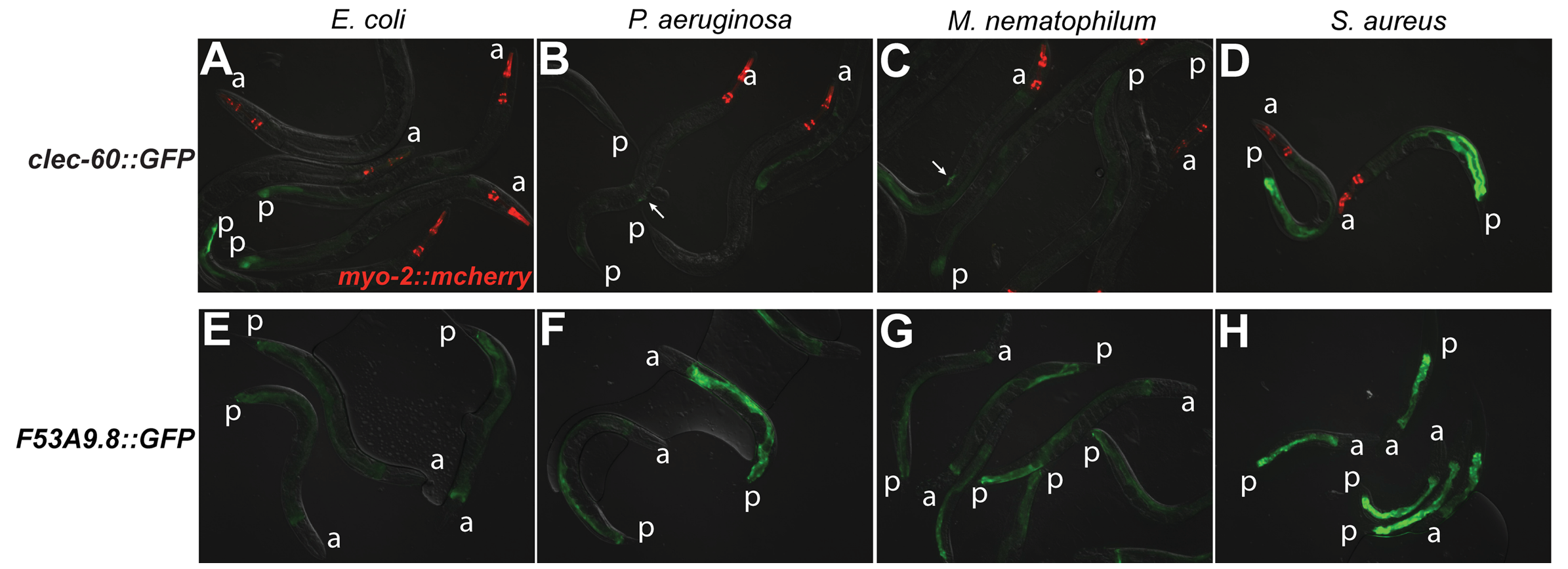

Supplement: Figure S11 — Pathogen-specific induction of infection reporters. A, B, C, D. Animals carrying clec-60::gfp arrays were infected with pathogens for 24 h, in parallel with non-pathogenic E. coli control (A). Induction of clec-60::gfp by infection with M. nematophilum (C) and S. aureus (D), and repression by infection with P. aeruginosa (B). Note vulval expression in B and C (arrows). E, F, G, H. Animals carrying F53A9.8::gfp were infected with pathogens for 24 h, in parallel with non-pathogenic E. coli control (E). Induction of F53A9.8::gfp by infection with M. nematophilum (G), S. aureus (H), and P. aeruginosa (F); the levels of induction on S. aureus were highest. On non-pathogenic E. coli, clec-60::GFP was expressed at low levels, mostly in the 9th ring of intestinal epithelial cells (Fig. S7A), and F53A9.8::GFP was weakly expressed mostly in the posterior intestine and the rectal gland cells (Fig. S7E). During infection with M. nematophilum, clec-60::GFP was expressed weakly in the intestine, as well as occasional expression in the vulva, consistent with previous reports (Fig. S7C, [90]). M. nematophilum induced moderate levels of F53A9.8::GFP expression in the intestine (Fig. S7G). During infection with P. aeruginosa, we observed reduced expression of clec-60::GFP in the intestine, below the level observed on E. coli, except for occasional expression in the vulva (Fig. S7B), and induced expression of F53A9.8::GFP in the intestine (Fig. S7F). Finally, during infection with S. aureus we observed highest expression of both reporters, mostly in the posterior half of the intestine (Fig. S7D, H). clec-52::GFP was also downregulated on P. aeruginosa (not shown). (6.29 MB TIF) [file ppat.1000982.s011.tif]

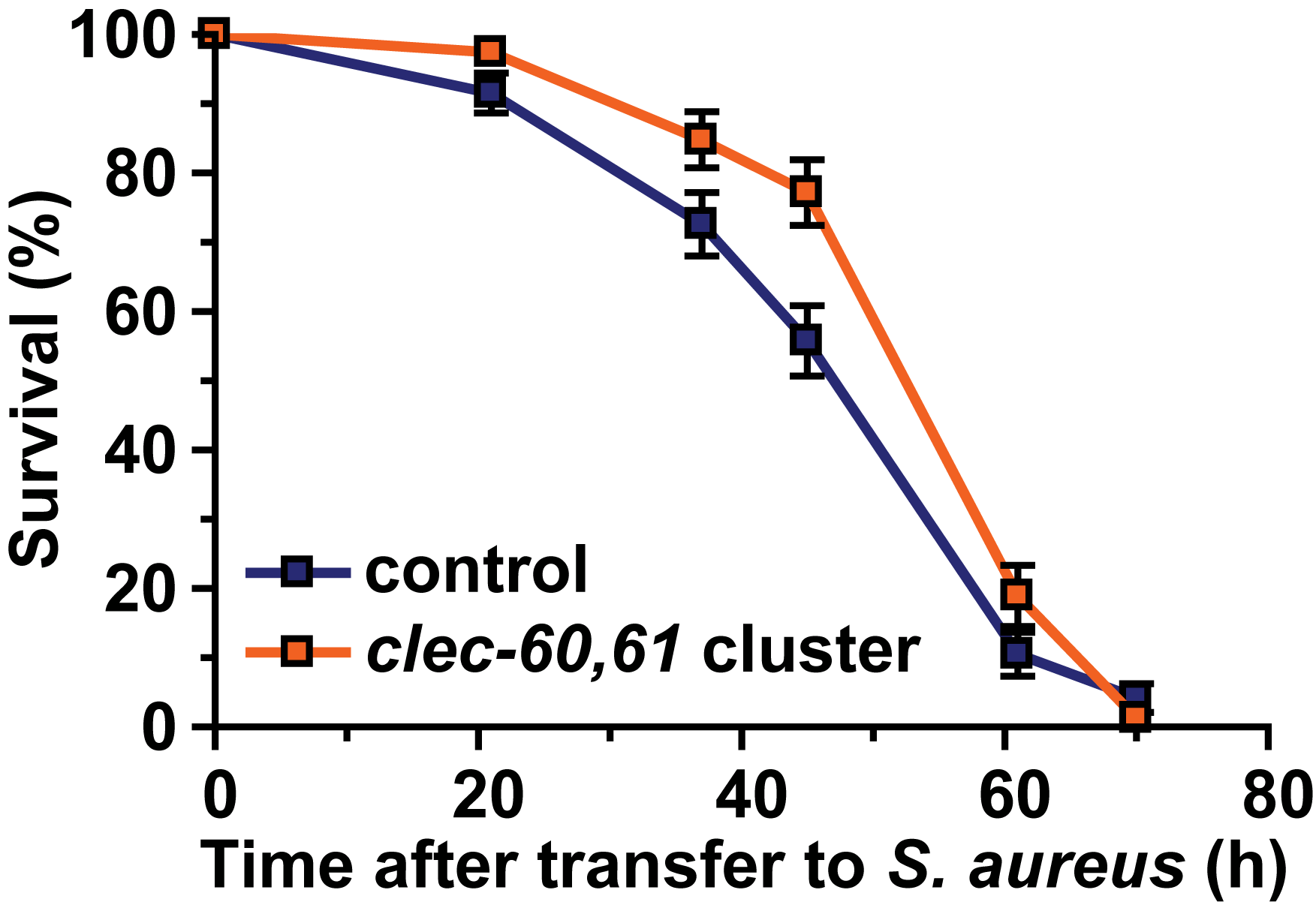

Supplement: Figure S12 — clec-60,61/CTL overexpression is protective during S. aureus infection. Transgenic animals carrying clec-60,61 cluster extrachromosomal arrays survived longer (LT50 = 54.2 h; N = 79; p = 0.019) during S. aureus infection than control animals bearing arrays composed of coinjection marker and clec-60::GFP promoter fusion (LT50 = 47.3 h; N = 95). (6.37 MB TIF) [file ppat.1000982.s012.tif]
