# Supplementary material for: Distinct Pathogenesis and Host Responses during Infection of C. elegans by P. aeruginosa and S. aureus
Source: PLoS Pathog. 2010 Jul 1;6(7):e1000982. doi: 10.1371/journal.ppat.1000982 (PMC2895663; doi:10.1371/journal.ppat.1000982)
Supplement: Table S4 — List of shared gene classes upregulated during infection of C. elegans and human neutrophils with S. aureus. (0.10 MB DOC) [file ppat.1000982.s016.doc]

**Supplemental Table 4:** Shared gene classes upregulated by *S. aureus* infection in *C. elegans* and human PMNs.

| **Class** | ***C. elegans*** | ***H. sapiens*** |
| --- | --- | --- |
| Transporter | | *T22F3.11* | Permease, major facilitator superfamily transporter | | --- | --- | | *H11E01.2* | Transporter | | *srsx-34* | Predicted transporter/transmembrane protein | | *pmp-1* | ABC transporter | | *ZK563.2* | Sodium-dependent phosphate transporter | | *T22F3.11* | Permease, major facilitator superfamily transporter | | *aqp-8* | aquaporin | | | *ABCA12* | ATP-binding cassette, sub-family A (ABC1), member 12 | | --- | --- | | *ABCC3* | ATP-binding cassette, sub-family C (CFTR/MRP), member 3 | | *ABCC6* | ATP-binding cassette, sub-family C (CFTR/MRP), member 6 | | *ABCF3* | ATP-binding cassette, sub-family F (GCN20), member 3 | | *ABCG4* | ATP-binding cassette, sub-family G (WHITE), member 4 | | *AQP4* | aquaporin 4 | | *AQP9* | aquaporin 9 | |
| UGT | | *ugt-18* | UDP-glucoronosyl/UDP-glucosyl transferase | | --- | --- | | *ugt-25* | UDP-glucoronosyl/UDP-glucosyl transferase | | | *B3GNT4* | UDP-GlcNAc:betaGal beta-1,3-N-acetylglucosaminyltransferase 4 | | --- | --- | | *B4GALT2* | UDP-Gal:betaGlcNAc beta 1,4- galactosyltransferase, polypeptide 2 | | *B4GALT5* | UDP-Gal:betaGlcNAc beta 1,4- galactosyltransferase, polypeptide 5 | | *UGT1A6* | UDP glucuronosyltransferase 1 family, polypeptide A6 | | *UGT1A8* | UDP glucuronosyltransferase 1 family, polypeptide A8 | | *UGT2A1* | UDP glucuronosyltransferase 2 family, polypeptide A1 | |
| **Cl^-^** channel | | *clh-1* | Chloride channel protein | | --- | --- | | | *CLCA2* | chloride channel, calcium activated, family member 2 | | --- | --- | | *CLCN5* | chloride channel 5 (nephrolithiasis 2, X-linked, Dent disease) | | *CLCN7* | chloride channel 7 | |
| C-type lectin | | *clec-71* | C-type lectin | | --- | --- | | *clec-60* | C-type lectin, von Willebrand factor, type A | | *clec-52* | C-type lectin | | *clec-72* | C-type lectin | | *clec-70* | C-type lectin | | *clec-61* | C-type lectin , von Willebrand factor, type A | | *F08H9.5* | C-type lectin domain, CUB domain | | *R06B10.3* | C-type lectin | | *clec-51* | C-type lectin | | *clec-7* | C-type lectin, CUB domain | | *clec-56* | C-type lectin domain, CUB domain | | *clec-82* | C-type lectin | | *clec-62* | C-type lectin domain, von Willebrand factor type A domain | | *C25B8.4* | Asialoglycoprotein receptor, C-type lectin | | | *CLEC4E* | C-type lectin domain family 4, member E | | --- | --- | | *CLEC5A* | C-type lectin domain family 5, member A | | *M6PR* | mannose-6-phosphate receptor (cation dependent) | |
| **peptidase/**  protease | | *B0285.7* | Puromycin-sensitive aminopeptidase and related aminopeptidases | | --- | --- | | *F19C7.2* | Lysosomal carboxypeptidase | | *cpr-2* | Cysteine protease | | *F21F8.4* | Protease | | *cpr-5* | Thiol protease | | *F21F8.2* | Protease | | *cpr-4* | Cathepsin B-like cysteine proteinase 4 precursor | | | *CPA4* | carboxypeptidase A4 | | --- | --- | | *CTSD* | cathepsin D (lysosomal aspartyl peptidase) | | *CTSE* | cathepsin E | | *CTSG* | cathepsin G | | *DNPEP* | aspartyl aminopeptidase | |
| CYP450 | | *cyp-37B1* | Cytochrome P450 | | --- | --- | | *cyp-34A9* | Cytochrome P450 | | *cyp-34A4* | Cytochrome P450 | | *cyp-32B1* | Cytochrome P450 | | *cyp-25A6* | Cytochrome P450 | | | *CYP1A2* | cytochrome P450, family 1, subfamily A, polypeptide 2 | | --- | --- | | *CYP27B1* | cytochrome P450, family 27, subfamily B, polypeptide 1 | | *CYP2A7* | cytochrome P450, family 2, subfamily A, polypeptide 7 | | *CYP2C18* | cytochrome P450, family 2, subfamily C, polypeptide 18 | | *CYP2C9* | cytochrome P450, family 2, subfamily C, polypeptide 9 | | *CYP7A1* | cytochrome P450, family 7, subfamily A, polypeptide 1 | | *CYP7B1* | cytochrome P450, family 7, subfamily B, polypeptide 1 | |
| EGF-like domain | | *lam-2* | Laminin-type EGF-like domain | | --- | --- | | | *EGFL6* | EGF-like-domain, multiple 6 | | --- | --- | |
| F-box | | *C25H3.10* | Cyclin-like F-box | | --- | --- | | *F36H5.8* | Cyclin-like F-box | | *fbxb-10* | Protein of unknown function DUF38, Cyclin-like F-box | | *C02F5.7* | Leucine-rich repeat, Cyclin-like F-box | | | *FBXO22* | F-box protein 22 | | --- | --- | | *FBXO4* | F-box protein 4 | | *FBXO5* | F-box protein 5 | |
| FGD1 | | *exc-5* |  | | --- | --- | | | *FGD1* | FYVE, RhoGEF and PH domain containing 1 (faciogenital dysplasia) | | --- | --- | |
| LRR | | *C02F5.7* | Leucine-rich repeat, Cyclin-like F-box | | --- | --- | | | *FLRT3* | fibronectin leucine rich transmembrane protein 3 | | --- | --- | | *LGR4* | leucine-rich repeat-containing G protein-coupled receptor 4 | | *LRRC19* | leucine rich repeat containing 19 | | *LRRC50* | leucine rich repeat containing 50 | | *LRRTM4* | leucine rich repeat transmembrane neuronal 4 | |
| FMO | | *fmo-2* | Flavin-containing monooxygenase FMO | | --- | --- | | *fmo-1* | Flavin-containing monooxygenase FMO | | *C46H11.2* | Flavin-containing monooxygenase FMO | | | *FMO3* | flavin containing monooxygenase 3 | | --- | --- | | *FMO4* | flavin containing monooxygenase 4 | |
| GPCR related | | *srh-74* | 7TM chemoreceptor, subfamily 2, Short-chain dehydrogenase/reductase SDR | | --- | --- | | | *GPR109B* | G protein-coupled receptor 109B | | --- | --- | | *GPR116* | G protein-coupled receptor 116 | | *GPR126* | G protein-coupled receptor 126 | | *GPR137* | G protein-coupled receptor 137 | | *GPR175* | G protein-coupled receptor 175 | | *GPR32* | G protein-coupled receptor 32 | | *GPR85* | G protein-coupled receptor 85 | | *OR1D2* | olfactory receptor, family 1, subfamily D, member 2 | | *OR1E1* | olfactory receptor, family 1, subfamily E, member 1 | | *OR2H2* | olfactory receptor, family 2, subfamily H, member 2 | | *OR7E24* | olfactory receptor, family 7, subfamily E, member 24 | | *RGS12* | regulator of G-protein signalling 12 | | *RGS16* | regulator of G-protein signalling 16 | | *RGS7* | regulator of G-protein signalling 7 | |
| GST | | *gst-28* | Glutathione S-transferase | | --- | --- | | | *GSTA4* | glutathione S-transferase A4 | | --- | --- | | *GSTM3* | glutathione S-transferase M3 (brain) | | *GSTO1* | glutathione S-transferase omega 1 | | *GSTT1* | glutathione S-transferase theta 1 | |
| histone | | *hil-1* | Histone H1 like | | --- | --- | | | *HIST1H2AE* | histone 1, H2ae | | --- | --- | | *HIST1H3E* | histone 1, H3e | |
| galectin | | *lec-10* | Galectin, galactose-binding lectin | | --- | --- | | | *LGALS3* | lectin, galactoside-binding, soluble, 3 (galectin 3) | | --- | --- | | *LGALS3BP* | lectin, galactoside-binding, soluble, 3 binding protein | |
| MAPK | | *mpk-2* | MAP kinase | | --- | --- | | | *MAP2K3* | mitogen-activated protein kinase kinase 3 | | --- | --- | | *MAP2K5* | mitogen-activated protein kinase kinase 5 | | *MAP3K9* | mitogen-activated protein kinase kinase kinase 9 | | *MAP4K4* | mitogen-activated protein kinase kinase kinase kinase 4 | | *MAPK6* | mitogen-activated protein kinase 6 | |
