# Supplementary material for: Distinct Pathogenesis and Host Responses during Infection of C. elegans by P. aeruginosa and S. aureus
Source: PLoS Pathog. 2010 Jul 1;6(7):e1000982. doi: 10.1371/journal.ppat.1000982 (PMC2895663; doi:10.1371/journal.ppat.1000982)
Supplement: Table S6 — A. List of C. elegans strains used in this study. B. List of bacterial strains used in this study. (0.06 MB DOC) [file ppat.1000982.s018.doc]

**Table S6.**

**A. *C. elegans* strains.**

| Name | Relevant genotype | Source | Reference |
| --- | --- | --- | --- |
| AU0157 | agEx39[*myo-2::mCherry,clec-60::gfp*] |  | this study |
| BC11374 | *dpy-5*(e907)I; sEx11374 [F53A9.8::*gfp,dpy-5+*] | BC *C. elegans* Gene Expression Consortium |  |
| CF512 | *fer-15*(b26)II; *fem-1*(hc17)IV | CGC | [3] |
| N2 Bristol | wild type | CGC | [8] |
| SD1164 | *pha-1*(e2123)III; Ex [*clec-52::gfp,pha-1+* (pC1)] | S. Kim | [11] |
| AU0279 | agEx97[*myo-2::mCherry,clec-70::gfp*] |  | this study |
| AU0280 | agEx98[*myo-2::mCherry,fmo-2::gfp*] |  | this study |
| AU0281 | agEx99[*myo-2::mCherry,ilys-3::gfp*] |  | this study |
| AU0172 | *tol-1*(nr2033)I, backcrossed 1X |  | [1] |
| AU0282 | agEx100[*myo-2::mCherry, lys-4,5*] |  | this study |
| AU0283 | agEx101[*myo-2::mCherry, clec-60,61*] |  | this study |
| AU0283 | agEx102[*myo-2::mCherry,clec-70,71*] |  | this study |
| MH37 | *mpk-1*(ku1),*unc-32*(e189)III | CGC |  |
| CB189 | *unc-32*(e189)III | CGC |  |
| AU0285 | *bar-1* (ga80)X, backcrossed 3X | CGC | [10] |
| AU0286 | *egl-5* (n486)III, backcrossed 3X | CGC | [2] |
| AU0066 | *pmk-1*(km25)IV, backcrossed 3X | K. Matsumoto | [3] |
| GR1373 | *eri-1*(mg366)IV | G. Ruvkun | [9] |
| AU0287 | agIs38[*myo-2::mCherry, clec-70,71*] |  | this study |

**B. Bacterial strains.**

| Strain | Notes | Reference |
| --- | --- | --- |
| *E. coli* OP50 | *Ura^-^* *Str***^R^** | [8] |
| *P. aeruginosa* PA14 | Pathogenic isolate | [4] |
| *P. aeruginosa gacA* | PA14 *gacA::Gm^R^* | [4] |
| *S. aureus* NCTC8325 | Wild-type strain; *rsbU* mutant | [6] |
| *M. nematophilum* CBX102 | Wild-type strain | [5] |
| *S. aureus* ALC1435 | RN6390 (pALC1420, *Cm*^R^) *sarA* P1 promoter::*gfp*_uv_ | [6] |
| *S. aureus* RN6390 | NCTC8325-4; Prophage-cured derivative of NCTC8325 | [6] |
| *S. aureus* DU5938 | NCTC8325-4 *hla::Em^R^ hlb::Φ42E hlg::Tc^R^* | [7] |
| *S. aureus* DU5720 | NCTC8325-4 *hla::Em^R^ hlb::Φ42E* | [7] |
| *S. aureus* DU5945 | NCTC8325-4 *hla::Em^R^ hlg::Tc^R^* | [7] |
| *S. aureus* DU5946 | NCTC8325-4 *hlb::Φ42E hlg::Tc^R^* | [7] |
| *S. aureus* NCTC8325-4 | Prophage-cured derivative of NCTC8325 | [7] |
| *P. aeruginosa* PA14 - GFP | GFP-expressing strain | [4] |

Bibliography

1. Pujol N, Link EM, Liu LX, Kurz CL, Alloing G, et al. (2001) A reverse genetic analysis of components of the Toll signaling pathway in Caenorhabditis elegans. Curr Biol 11: 809-821.

2. Ferreira HB, Zhang Y, Zhao C, Emmons SW (1999) Patterning of Caenorhabditis elegans posterior structures by the Abdominal-B homolog, egl-5. Developmental Biology 207: 215-228.

3. Troemel ER, Chu SW, Reinke V, Lee SS, Ausubel FM, et al. (2006) p38 MAPK regulates expression of immune response genes and contributes to longevity in C. elegans. PLoS Genet 2: e183.

4. Tan MW, Mahajan-Miklos S, Ausubel FM (1999) Killing of Caenorhabditis elegans by Pseudomonas aeruginosa used to model mammalian bacterial pathogenesis. Proc Natl Acad Sci USA 96: 715-720.

5. Gravato-Nobre MJ, Nicholas HR, Nijland R, O'rourke D, Whittington DE, et al. (2005) Multiple genes affect sensitivity of Caenorhabditis elegans to the bacterial pathogen Microbacterium nematophilum. Genetics 171: 1033-1045.

6. Sifri CD, Begun J, Ausubel FM, Calderwood SB (2003) Caenorhabditis elegans as a model host for Staphylococcus aureus pathogenesis. Infect Immun 71: 2208-2217.

7. Nilsson IM, Hartford O, Foster T, Tarkowski A (1999) Alpha-toxin and gamma-toxin jointly promote Staphylococcus aureus virulence in murine septic arthritis. Infect Immun 67: 1045-1049.

8. Brenner S (1974) The genetics of Caenorhabditis elegans. Genetics 77: 71-94.

9. Kennedy S, Wang D, Ruvkun G (2004) A conserved siRNA-degrading RNase negatively regulates RNA interference in C. elegans. Nature 427: 645-649.

10. Eisenmann DM, Maloof JN, Simske JS, Kenyon C, Kim SK (1998) The beta-catenin homolog BAR-1 and LET-60 Ras coordinately regulate the Hox gene lin-39 during Caenorhabditis elegans vulval development. Development 125: 3667-3680.

11. Pauli F, Liu Y, Kim YA, Chen P-J, Kim SK (2006) Chromosomal clustering and GATA transcriptional regulation of intestine-expressed genes in C. elegans. Development 133: 287-295.
