# Supplementary material for: Distinct Pathogenesis and Host Responses during Infection of C. elegans by P. aeruginosa and S. aureus
Source: PLoS Pathog. 2010 Jul 1;6(7):e1000982. doi: 10.1371/journal.ppat.1000982 (PMC2895663; doi:10.1371/journal.ppat.1000982)
Supplement: Table S7 — Over-represented GO annotations among gene expression changes during S. aureus or P. aeruginosa infection for 8 h. “Output is in the form of “Rank, N, M, X, LOD, P, P-adj, GO Attribute”, where Rank: position in the attribute list ranked by significance of association with query; N: number of genes in the most surprising subquery with this attribute; M: size of most surprising sub query; X: number of genes overall with this attribute; LOD: the logarithm (base 10) of the odds ratio; positive values indicate over-representation; P: single hypothesis one-sided P-value of the association between attribute and query (based on Fisher's Exact Test); P-adj: adjusted P-value: fraction (as a %) of 1000 null-hypothesis simulations having attributes with this single-hypothesis P value or smaller.” From FuncAssociate (http://llama.med.harvard.edu/cgi/func/funcassociate). (0.06 MB DOC) [file ppat.1000982.s019.doc]

**Table S7.** Over-represented GO annotations among gene expression changes during *S. aureus* or *P. aeruginosa* infection for 8 h.

***S. aureus* Up over-represented attributes**

| **Rank** | **N** | **M** | **X** | **LOD** | **P** | **P-adj** | **GO Attribute** |
| --- | --- | --- | --- | --- | --- | --- | --- |
| 1 | 15 | 178 | 269 | 0.630 | 1.2e-05 | 0.048 | 0005529: sugar binding |

***S. aureus* Down over-represented attributes**

| **Rank** | **N** | **M** | **X** | **LOD** | **P** | **P-adj** | **GO Attribute** |
| --- | --- | --- | --- | --- | --- | --- | --- |
| 1 | 47 | 569 | 160 | 0.959 | 4.4e‐25 | <0.001 | 0042302: structural constituent of cuticle |
| 2 | 47 | 513 | 194 | 0.896 | 4.5e‐23 | <0.001 | 0006817: phosphate transport |
| 3 | 47 | 513 | 216 | 0.834 | 5.8e‐21 | <0.001 | 0015698: inorganic anion transport |
| 4 | 47 | 513 | 226 | 0.809 | 4.2e‐20 | <0.001 | 0006820: anion transport |
| 5 | 56 | 461 | 536 | 0.508 | 5.1e‐12 | <0.001 | 0005198: structural molecule activity |
| 6 | 4 | 76 | 13 | 1.893 | 1e‐06 | 0.004 | 0005604: basement membrane |
| 7 | 5 | 498 | 8 | 1.566 | 6e‐06 | 0.025 | 0005319: lipid transporter activity/lipophorin |

***P. aeruginosa* Down over-represented attributes**

| **Rank** | **N** | **M** | **X** | **LOD** | **P** | **P‐adj** | **GO Attribute** |
| --- | --- | --- | --- | --- | --- | --- | --- |
| 1 | 6 | 592 | 7 | 1.931 | 8.8e‐08 | 0.001 | 0004372: glycine hydroxymethyltransferase activity |
| 2 | 4 | 187 | 5 | 2.291 | 2.7e‐07 | 0.003 | 0016742: hydroxymethyl‐, formyl‐ and related transferase activity |
| 3 | 26 | 324 | 339 | 0.513 | 1.3e‐06 | 0.011 | 0040011: locomotion |
| 4 | 5 | 203 | 13 | 1.590 | 1.4e‐06 | 0.013 | 0005604: basement membrane |
| 5 | 3 | 187 | 3 | 2.657 | 3.6e‐06 | 0.026 | 0003937: IMP cyclohydrolase activity/inosinicase |
| 6 | 3 | 187 | 3 | 2.657 | 3.6e‐06 | 0.026 | 0004643: phosphoribosyl-aminoimidazole carboxamide formyltransferase activity |
| 7 | 5 | 592 | 7 | 1.636 | 5.2e‐06 | 0.034 | 0006544: glycine metabolism |
