# Supplementary material for: Distinct Pathogenesis and Host Responses during Infection of C. elegans by P. aeruginosa and S. aureus
Source: PLoS Pathog. 2010 Jul 1;6(7):e1000982. doi: 10.1371/journal.ppat.1000982 (PMC2895663; doi:10.1371/journal.ppat.1000982)
Supplement: Text S1 — Supporting text. (0.02 MB DOC) [file ppat.1000982.s020.doc]

**Supplementary Text:**

To determine the physiological roles of the host response, we used two complementary methods to study the over-representation of gene ontology (GO) classes in the *C. elegans* host response to *S. aureus* (see Materials and Methods for description). These analyses revealed upregulation of detoxifying and antimicrobial responses and downregulation of growth-related metabolic pathways and structural components (Table S7).

First, significance of representation analysis revealed that the most significantly induced class contains sugar-binding proteins including C-type lectins (CTLs, N=15, *p*=1.2E-5). The most significantly repressed GO classes contain genes encoding structural constituents of the cuticle (*e.g.* collagens; N=47, *p*=4.4E-25), phosphate and inorganic anion transport (N=47, *p*=4.5E-25 and *p=*5.8E-21), basement membranes (N=4, *p*=1E-6), and lipid transporters (N=5, *p*=6E-6) (Table S7).

Second, using NextBio, an alternative method to analyze significantly over-represented genes according to previously defined biologically-relevant groups (biogroups, [91]), we found among the induced genes an over-representation of putative detoxification factors (monooxygenases), lysozymes, ER proteins, heme binding proteins, and sugar binding proteins (Table S2a). Among over-represented repressed biogroups were collagens, wounding response factors and extracellular matrix components, and enzymes involved in aromatic compound metabolism, anion transport, alcohol metabolism, lipid biosynthesis, and precursor metabolite generation (Table S2a).
